# 2*H*-1,4-Benzoxazin-3(4*H*)-one linked 1,2,3-triazole derivatives and their study on inducing DNA damage in tumor cells

**DOI:** 10.3389/fphar.2025.1564090

**Published:** 2025-08-15

**Authors:** Xixi Hou, Yajie Guo, Xi Wang, En Gao, Jianxue Yang

**Affiliations:** ^1^ The First Affiliated Hospital, and College of Clinical Medicine of Henan University of Science and Technology, Luoyang, China; ^2^ Department of Emergency, The Eighth Affiliated Hospital, Sun Yat-Sen University, Shenzhen, China; ^3^ School of Chemistry and Chemical Engineering, Henan Normal University, Xinxiang, China; ^4^ State Key Laboratory of Quality Research in Chinese Medicine/Macau Institute for Applied Research in Medicine and Health, Macau University of Science and Technology, Macau, China

**Keywords:** 2H-1, 4-benzoxazin-3(4H)-one, 1,2,3-triazole, anti-tumor DNA damage, apoptosis

## Abstract

This study explores the anticancer potential of rigid planar compounds capable of intercalating into tumor cell DNA, thereby inducing DNA damage and subsequent cell death. A novel series of compounds (**c1–c20**) were synthesized using 2*H*-1,4-benzoxazin-3(4*H*)-one as the planar core structure, with 1,2,3-triazole groups introduced at the 7-position. Biological evaluation across multiple human tumor cell lines revealed that several c-series compounds exhibited notable inhibitory activity against Huh-7 liver cancer cells, with IC_50_ values of 28.48 μM (**c5**), 32.60 μM (**c14**), 31.87 μM (**c16**), and 19.05 μM (**c18**). Mechanistic studies indicated that these compounds effectively induced DNA damage, as evidenced by the upregulation of γ-H2AX, and triggered apoptosis via increased caspase-7 expression. Moreover, enhanced LC3 expression and autophagosome formation suggested the activation of autophagy pathways. The observed biological activities are likely attributed to the rigid planar configuration of the molecules, which facilitates DNA intercalation. Collectively, these results highlight the potential of these synthesized compounds as promising lead candidates for the development of novel therapeutic agents against liver cancer. Importantly, acute toxicity studies in mice demonstrated that these compounds possess favorable safety profiles, further supporting their potential for future preclinical development.

## 1 Introduction

Cancer remains a major global health challenge, driving the urgent need for effective anti-tumor therapeutics. Among various strategies, targeting tumor cell DNA to induce damage has garnered significant attention due to its distinct mechanism and therapeutic promise (Macan et al., 2019; Pokhodylo et al., 2024). As the genetic blueprint of the cell, DNA plays a central role in maintaining genomic stability and supporting cell survival. Disrupting DNA integrity in tumor cells can effectively inhibit proliferation and trigger apoptosis, thereby exerting anticancer effects (Ueda et al., 2019; Juretschke and Beli, 2021). Conventional chemotherapeutic agents such as cisplatin, cyclophosphamide, and paclitaxel primarily act by binding to DNA or disrupting its replication. However, these agents often suffer from poor selectivity and cause substantial toxicity in normal tissues (Eliopoulos et al., 2016; Nagata et al., 2018). As a result, there is an increasing focus on developing DNA-targeting drugs with enhanced selectivity and reduced systemic toxicity. Recent advances in targeted molecular therapy have enabled more precise approaches to inducing DNA damage in cancer cells. One promising strategy involves the design of small molecules that intercalate into DNA (Rodriguez-Rocha et al., 2011), thereby activating DNA damage response pathways, impairing tumor cell differentiation, or inducing cell death through apoptosis and autophagy (Yao et al., 2021; Zhou et al., 2023).

**FIGURE 1:**
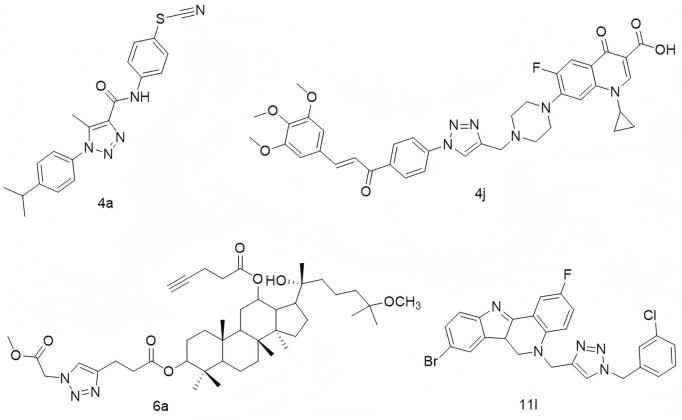
Structures of compound **4a**, compound **4j**, compound **6a**, and compound **11l**.

1,2,3-Triazoles constitute a prominent class of five-membered heterocyclic compounds characterized by a rigid, planar structure, a π–π conjugated system, and a high dipole moment (Guan et al., 2024; Mao et al., 2020; Cidade, 2021; Zhou et al., 2023). These structural features enable them to engage in diverse non-covalent interactions—including hydrophobic forces, van der Waals forces, and hydrogen bonding—with a wide array of enzymes and receptors. Consequently, 1,2,3-triazoles exhibit broad-spectrum pharmacological activities, such as anti-tumor, antiviral, anti-inflammatory, and antibacterial effects (Xu et al., 2019; Begam et al., 2022; Hou et al., 2024; Liu et al., 2024). Of particular interest is their ability to induce DNA damage in tumor cells, either through direct intercalation into DNA or by modulating associated signaling pathways, highlighting their potential as scaffolds for the development of DNA-damaging anticancer agents. For instance, Stoika’s group developed N-(4-thiocyanatophenyl)-1*H*-1,2,3-triazole-4-carboxamides, among which compound **4a** (Figure 1) was found to reduce mitochondrial membrane potential and induce DNA damage in Jurkat cells, despite not directly intercalating into DNA (Pokhodylo et al., 2022). Similarly, Abbas and colleagues synthesized a series of 1,2,3-triazole-linked ciprofloxacin-chalcone hybrids. Compound **4j** (Figure 1) exhibited potent cytotoxicity against HCT116 colon cancer cells (IC_50_ = 2.53 μM), comparable to doxorubicin (IC_50_ = 1.22 μM). Mechanistic studies revealed that **4j** inhibited topoisomerases I and II, disrupted tubulin polymerization, elevated γ-H2AX expression, and arrested cells at the G2/M phase, likely via activation of the ATR/CHK1/Cdc25C pathway (Mohammed et al., 2022). Zhao’s group employed click chemistry to couple a 1,2,3-triazole unit with the 25-OCH_3_-PPD scaffold, yielding compounds capable of inducing apoptosis and DNA damage; among them, compound **6a** (Figure 1) showed strong inhibitory activity against A549 lung cancer cells (Han et al., 2024). Likewise, Tummatorn’s team designed hybrid molecules combining 8-bromo-2-fluoro-isocryptolepine with a triazole moiety. Compound **11l** effectively suppressed A549 cell proliferation (IC_50_ = 3.01 μM) and induced G2/M phase arrest via p53 upregulation, indicating DNA damage induction (Figure 1).

**FIGURE 2:**
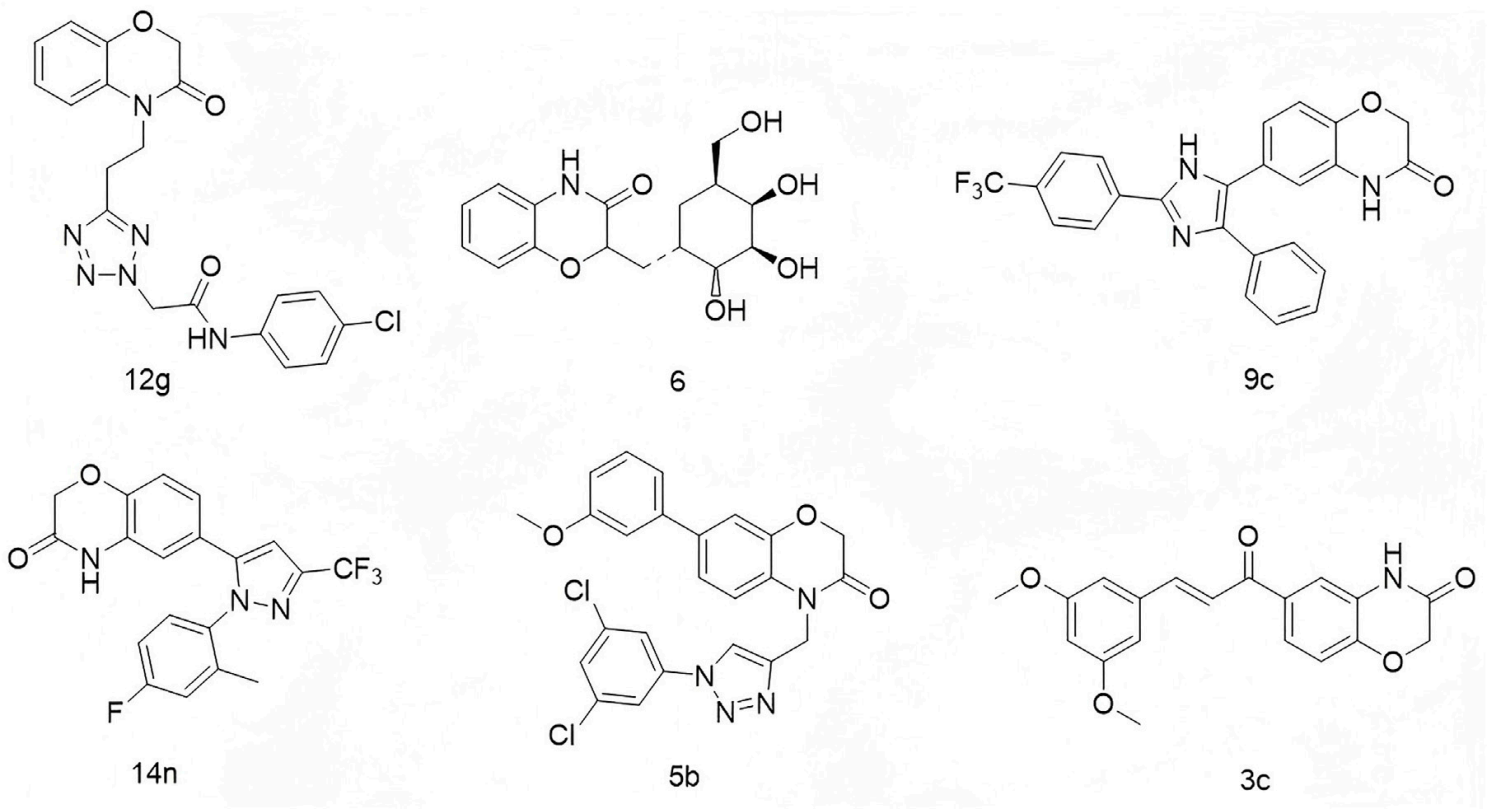
Structures of compound **12g**, compound **6**, compound **9c**, compound **14n**, compound **5b**, and compound **3c**.

2*H*-1,4-Benzoxazin-3(4*H*)-one is another rigid planar heterocycle that has attracted attention for its promising biological activities and low toxicity profile, making it a valuable scaffold in drug discovery and development (Bollu et al., 2017; Yan et al., 2019). For example, Thirukovela’s group developed 2*H*-1,4-benzoxazin-3(4*H*)-one–amide hybrids, among which compound **12g** (Figure 2) exhibited potent EGFR inhibitory activity (IC_50_ = 0.46 μM) and superior efficacy against breast cancer cell lines compared to erlotinib (Sagam et al., 2024). Feng et al. synthesized compound **6**, a derivative of 2*H*-1,4-benzoxazin-3(4*H*)-one, which demonstrated antibacterial, antifungal, and potential antidepressant effects (Feng et al., 2010). Manojit’s group reported compound **9c** (Figure 2), which significantly reduced the viability of A549, DLD-1, and MV4-11 cancer cells, again outperforming erlotinib (Feng et al., 2010). Fukumoto’s team designed compound **14n** (Figure 2), a highly selective mineralocorticoid receptor antagonist with an IC_50_ of 41 nM and excellent selectivity over related steroid hormone receptors (Hasui et al., 2011). In another study, Reddy’s group synthesized a series of 1,2,3-triazole-containing 2*H*-1,4-benzoxazin-3(4*H*)-one derivatives. Among them, compound **5b** (Figure 2) displayed cytotoxicity against MCF-7 and HeLa cells, with IC_50_ values of 17.08 μg/mL and 15.38 μg/mL, respectively (Nagavelli et al., 2016). Zhao’s group further expanded the scaffold by developing compound **3c** (Figure 2), which showed potent inhibition of A549 cell proliferation (IC_50_ = 3.29 μM), accompanied by increased LC3 expression, autophagy induction, G1 phase arrest, and nuclear condensation (Zhou et al., 2014).

Given the rigid planar structures and widespread use of both 1,2,3-triazole and 2*H*-1,4-benzoxazin-3(4*H*)-one in anti-tumor drug development, we propose to modify the 2*H*-1,4-benzoxazin-3(4*H*)-one scaffold with 1,2,3-triazole groups in our ongoing efforts to discover novel lead compounds. This strategy aims to develop compounds capable of inducing DNA damage in tumor cells, contributing to the advancement of anticancer therapeutics.

## 2 Chemistry

**SCHEME 1:**
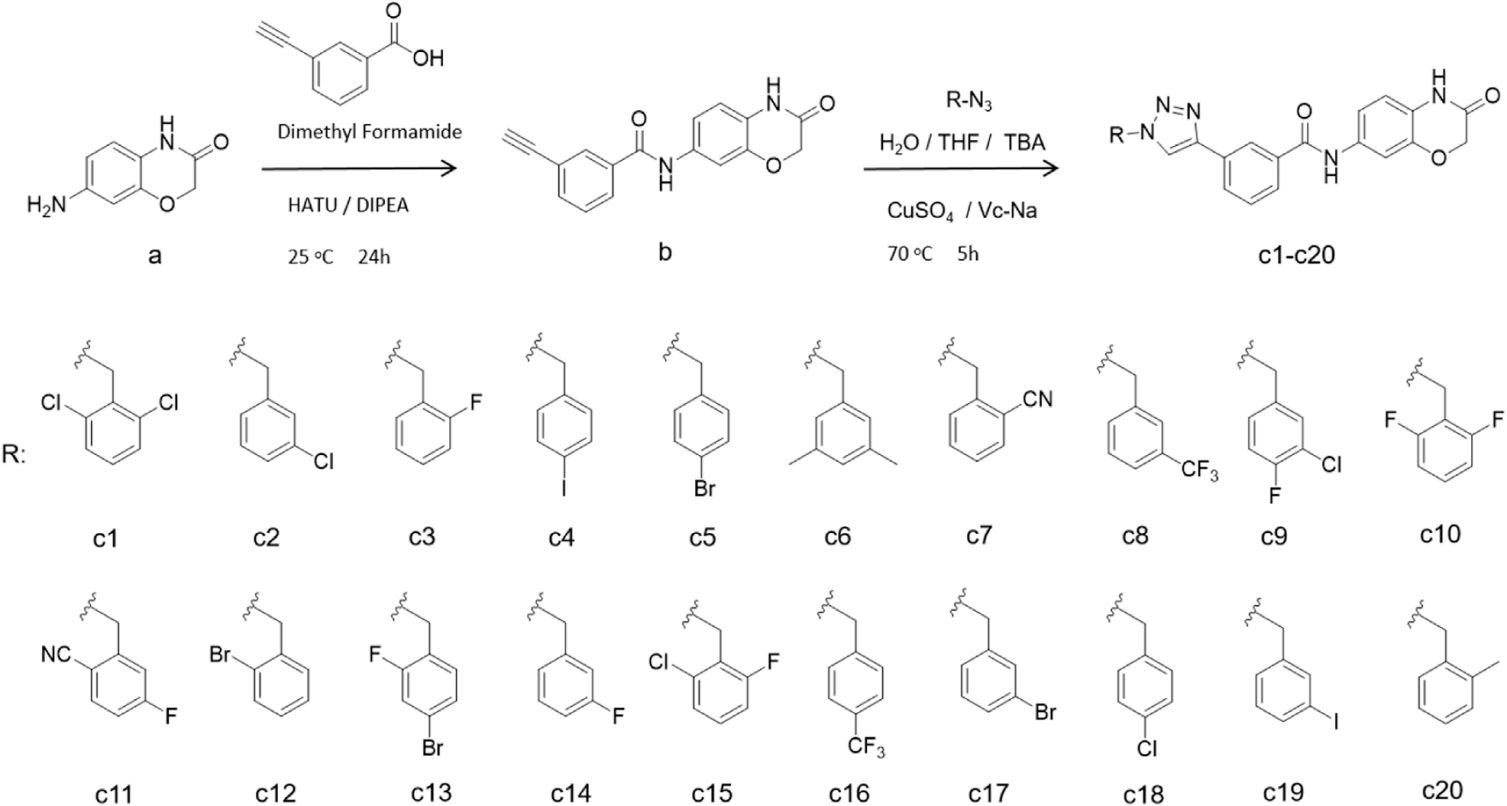
The reaction routes to compounds **c1**–**c20**.

In this synthesis route, 7-amino-2*H*-benzo[*b*][1,4]oxazin-3(4*H*)-one (compound **a**) was condensed with 3-ethynylbenzoic acid using HATU and DIPEA, yielding terminal alkyne compounds **b** (Hou et al., 2024). These compounds were then reacted with various azide compounds, resulting in the formation of 20 novel target compounds (**c1–c20**) (Hou et al., 2025), as shown in Scheme 1 and Table 1. The structures of the target compound were confirmed through ^1^H and ^13^C nuclear magnetic resonance (^1^H NMR and ^13^C NMR) spectroscopy.

**TABLE 1:** Cell viability of compounds **c1**–**c20**.

No.	Huh-7	MCF-7	A549	L02
**c1**	83.49 ± 0.78	109.73 ± 3.84	97.38 ± 3.35	84.09 ± 1.20
**c2**	17.54 ± 1.15	102.76 ± 1.63	95.59 ± 2.98	102.75 ± 2.36
**c3**	12.06 ± 0.58	108.28 ± 0.68	92.22 ± 3.87	101.30 ± 3.04
**c4**	21.36 ± 4.91	95.86 ± 2.14	94.01 ± 1.88	115.24 ± 0.81
**c5**	15.07 ± 0.43	106.90 ± 2.85	90.80 ± 5.32	100.67 ± 3.35
**c6**	25.56 ± 1.32	84.85 ± 3.50	81.60 ± 5.09	99.71 ± 2.50
**c7**	89.06 ± 3.02	89.21 ± 3.16	87.71 ± 6.40	100.78 ± 3.14
**c8**	12.97 ± 0.72	100.57 ± 4.50	86.06 ± 5.46	103.37 ± 3.10
**c9**	18.81 ± 0.56	109.80 ± 2.58	86.22 ± 4.40	102.77 ± 3.98
**c10**	24.94 ± 0.39	81.63 ± 1.46	61.08 ± 3.58	68.46 ± 2.04
**c11**	98.01 ± 1.22	92.96 ± 0.64	85.44 ± 2.84	110.62 ± 2.90
**c12**	86.09 ± 1.69	87.15 ± 0.55	87.71 ± 3.88	104.82 ± 2.71
**c13**	17.74 ± 1.58	98.90 ± 2.03	78.32 ± 2.72	115.24 ± 1.35
**c14**	18.05 ± 0.76	105.56 ± 1.48	77.47 ± 3.10	115.78 ± 1.18
**c15**	75.84 ± 2.97	84.57 ± 1.32	86.51 ± 2.25	113.68 ± 1.08
**c16**	17.67 ± 1.27	101.63 ± 0.92	76.81 ± 4.31	117.08 ± 1.41
**c17**	19.24 ± 1.88	90.41 ± 0.51	71.31 ± 2.90	111.66 ± 0.92
**c18**	17.09 ± 0.43	103.15 ± 1.83	89.76 ± 2.89	117.21 ± 1.27
**c19**	28.49 ± 3.64	97.24 ± 2.06	80.51 ± 3.81	103.37 ± 0.62
**c20**	23.64 ± 0.25	112.14 ± 4.59	97.85 ± 0.92	119.07 ± 0.57

## 3 Results and discussion

### 3.1 Cell viability of novel synthetic compounds

To assess the cytotoxic potential of the newly synthesized compounds **c1–c20**, a panel of human tumor cell lines—including Huh-7 (hepatocellular carcinoma), MCF-7 (breast cancer), and A549 (lung cancer)—was treated with each compound at a concentration of 50 μM for 72 h. Cell viability and cytotoxicity were evaluated using the CCK-8 assay. As summarized in Table 1, most compounds exhibited notable anti-proliferative activity against Huh-7 cells, while showing comparatively weaker effects on MCF-7 and A549 cells. Exceptions included **c1**, **c7**, **c11**, **c12**, and **c15**, which displayed limited activity against Huh-7 cells. To assess the selectivity, the compounds were further tested on L02 normal liver cells, revealing minimal cytotoxicity, as cell viability remained largely unaffected. The half-maximal inhibitory concentrations (IC_50_) of the most active compounds against Huh-7 cells were determined using a dose–response analysis over 72 h, with IC_50_ values for **c5**, **c14**, **c16**, and **c18** calculated as 28.48 μM, 32.60 μM, 31.87 μM, and 19.05 μM, respectively (Table 2). Structure–activity relationship (SAR) analysis revealed a potential correlation between molecular substitution patterns and cytotoxic efficacy. Specifically, inactive compounds such as **c1**, **c3**, **c7**, **c10**, **c11**, **c12**, **c13**, and **c15** featured substituents at the ortho-position of the triazole moiety, whereas the more active compounds—**c5**, **c14**, **c16**, and **c18**—lacked ortho-substituents. These findings suggest that steric hindrance at the ortho-position may negatively impact the compounds’ anti-tumor activity.

**TABLE 2:** IC_50_ values of some of the new compounds.

Compd no.	Huh-7, IC_50_ (μM)	Compd no.	Huh-7, IC_50_ (μM)
	72 h		72 h
**c2**	33.40 ± 0.80	**c13**	>50
**c3**	43.41 ± 0.08	**c14**	32.60 ± 1.23
**c4**	33.21 ± 0.11	**c16**	31.87 ± 0.04
**c5**	28.48 ± 0.29	**c17**	35.05 ± 1.84
**c6**	>50	**c18**	19.05 ± 0.12
**c8**	38.48 ± 0.24	**c19**	36.19 ± 0.09
**c9**	43.13 ± 2.43	**c20**	45.87 ± 0.50
**c10**	>50	**5-fluorouracil**	21.02 ± 6.37

### 3.2 Cell apoptosis of novel synthetic compounds

**FIGURE 3:**
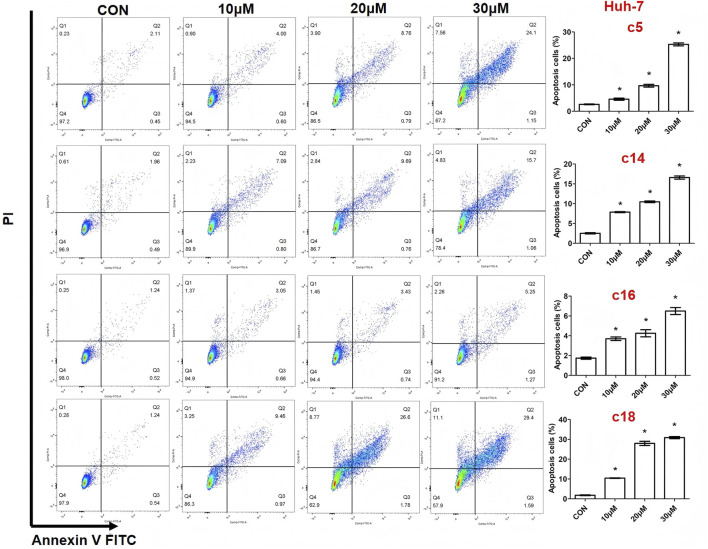
Compounds **c5**, **c14**, **c16**, and **c18** induced cell apoptosis. Huh-7 cells were exposed to different concentrations of compounds **c5**, **c14**, **c16**, and **c18** (10 μM, 20 μM, or 30 μM) for 72 h. Cells were collected and stained with Annexin V-FITC and PI, and apoptotic cells were measured by flow cytometry. Data were presented as means ± SEM, *P < 0.05.

Apoptosis, a programmed form of cell death, was investigated to understand the molecular mechanisms behind the anti-proliferative effects of the novel compounds. Huh-7 cells were treated with various concentrations (10 μM, 20 μM, or 30 μM) of compounds **c5**, **c14**, **c16**, and **c18** for 72 h. The cells were then stained with Annexin V-FITC and propidium iodide (PI) and analyzed by flow cytometry to determine the proportion of apoptotic cells. The analysis showed significant, dose-dependent increases in apoptosis (Figure 3).

### 3.3 2*H*-1,4-Benzoxazin-3(4*H*)-one linked 1,2,3-triazole derivatives affected cell growth

**FIGURE 4:**
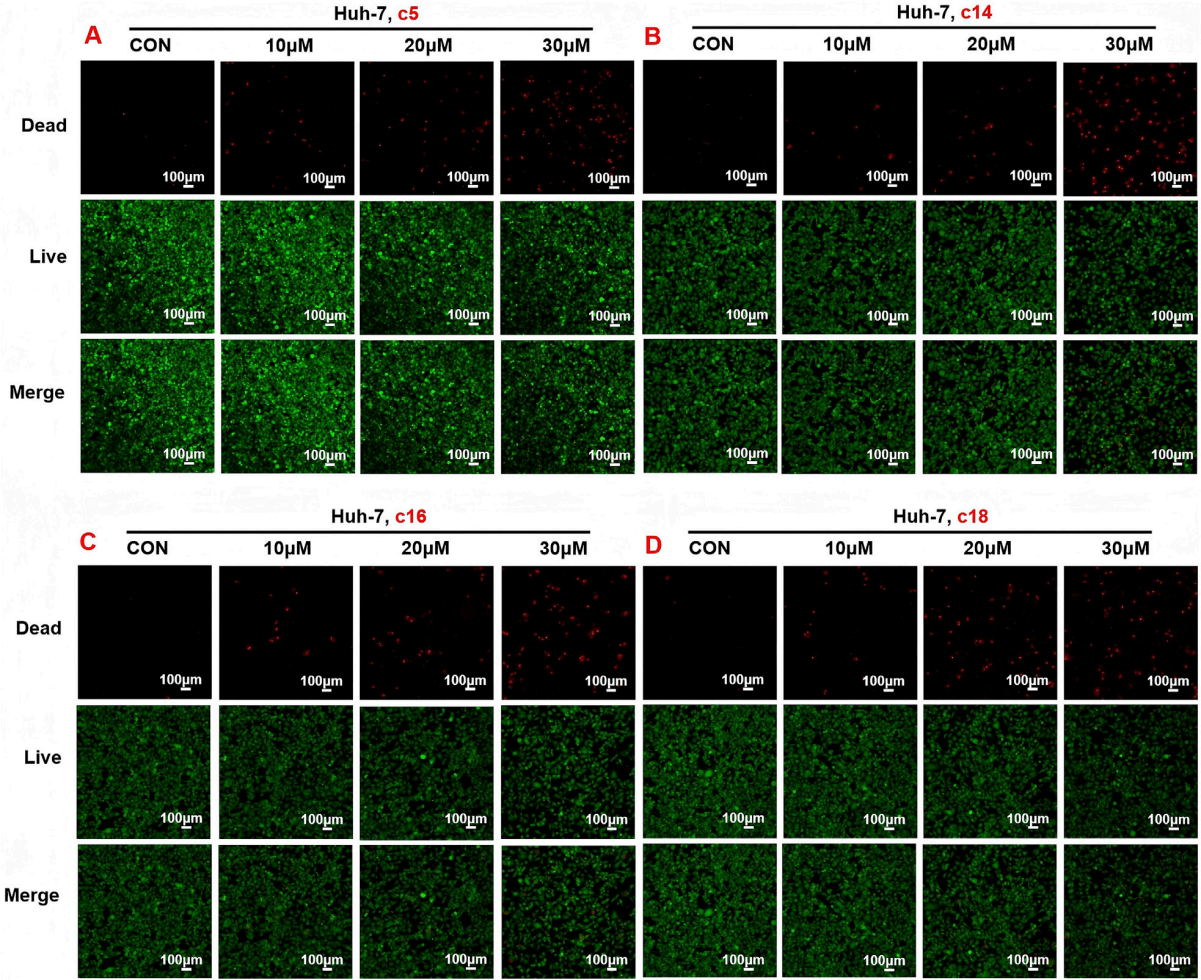
Compounds **c5**, **c14**, **c16**, and **c18** increased dead cells and decreased living cells. **(A–D)** Huh-7 cells were treated with different concentrations of compounds **c5**, **c14**, **c16**, and **c18** (10 μM, 20 μM, or 30 μM) for 24 h. Living and dead cells were stained with Calcein AM and PI, respectively, and then observed by confocal microscopy. Data were presented as means ± SEM; *P < 0.05.

To evaluate the anti-proliferative effects of 2*H*-1,4-benzoxazin-3(4*H*)-one linked 1,2,3-triazole derivatives, live/dead cell staining was performed using Calcein AM and PI on Huh-7 cells. The cells were treated with different concentrations (10 μM, 20 μM, or 30 μM) of compounds **c5**, **c14**, **c16**, and **c18** for 24 h. Living cells were stained green by Calcein AM, while dead cells were stained red by PI. The results showed a dose-dependent increase in dead cells, indicating effective inhibition of cell proliferation (Figures 4A–D).

### 3.4 Effects of 2*H*-1,4-benzoxazin-3(4*H*)-one linked 1,2,3-triazole derivatives on the gene expression of the cell growth process

**FIGURE 5:**
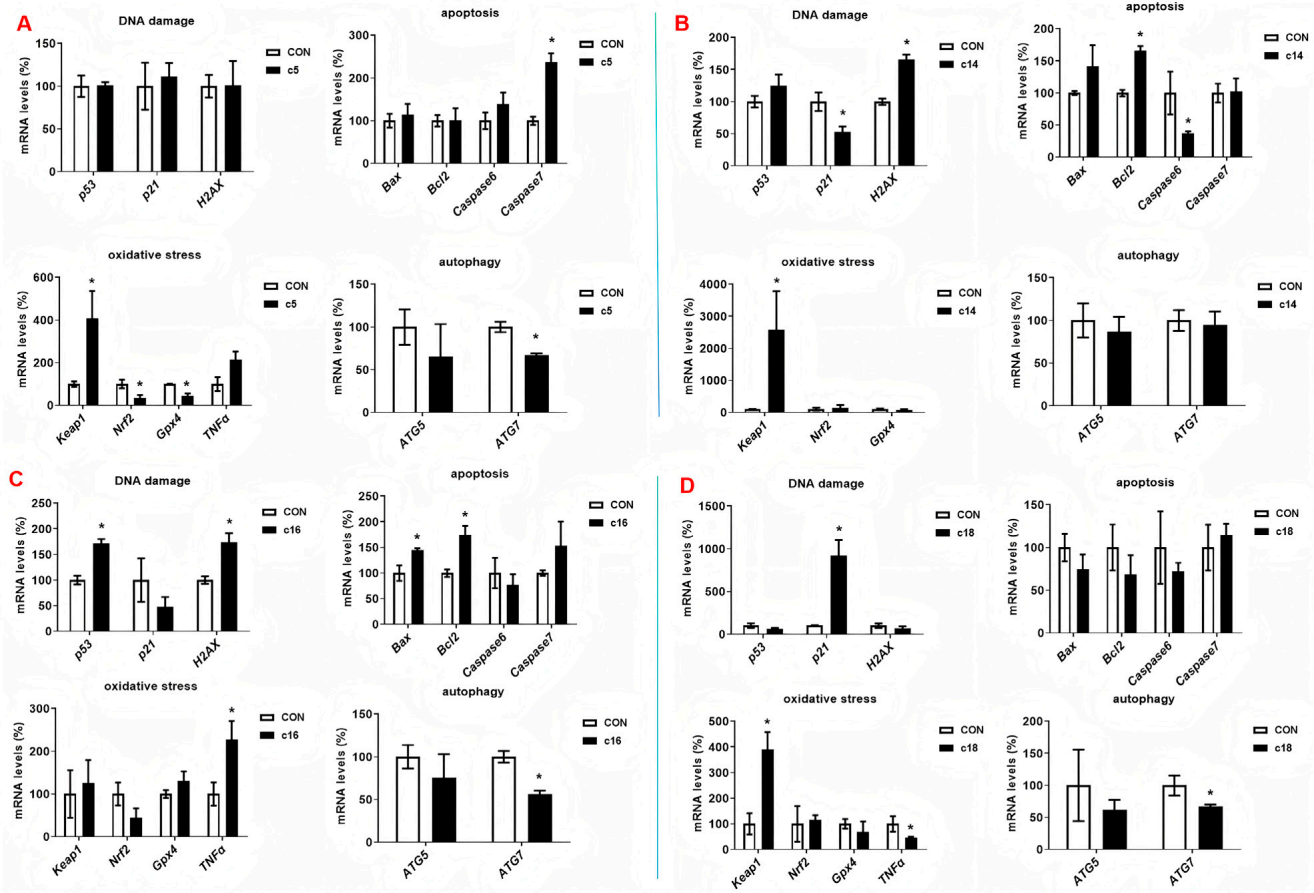
Compounds **c5**, **c14**, **c16**, and **c18** increased the gene expression. **(A–D)** Huh-7 cells were treated with different concentrations of compounds **c5**, **c14**, **c16**, and **c18** (20 or 30 μM) for 72 h. Gene expressions were detected by RT-PCR. Data were presented as means ± SEM; *P < 0.05.

To investigate the molecular mechanisms underlying the anti-tumor activity of 2*H*-1,4-benzoxazin-3(4*H*)-one linked 1,2,3-triazole derivatives, we examined the expression of key genes involved in cell growth and death, including those related to DNA damage, oxidative stress, apoptosis, and autophagy. Huh-7 cells were treated with compounds **c5** (30 μM), **c14** (30 μM), **c16** (30 μM), and **c18** (20 μM) for 72 h, followed by RT-PCR analysis. After **c5** treatment, DNA damage-related genes (*p53*, *p21*, and *H2AX*) showed no significant changes. However, the oxidative stress-related genes *Keap1* and *TNF-α* were significantly upregulated, while *Nrf2* and *Gpx4* were either downregulated or unchanged. *Caspase-7*, an apoptosis-related gene, was notably increased, consistent with earlier findings. The autophagy-related gene *ATG7* was downregulated, while *Atg5* expression remained unchanged (Figure 5A). In **c14**-treated cells, *H2AX* expression was significantly upregulated, although *p53* and *p21* were unchanged or downregulated. *Keap1* expression was also significantly increased, as was the apoptosis regulator *Bcl2*, whereas *Bax*, *caspase-6*, and *caspase-7* showed no significant changes. No differences were observed in autophagy-related gene expression (Figure 5B). In cells treated with **c16**, both *p53* and *H2AX* were markedly upregulated, as was *TNF-α*, a marker of oxidative stress. Consistently, *Bax* and *Bcl2* expressions were elevated, while *ATG7* was downregulated (Figure 5C). For **c18**-treated cells, *p21* expression was dramatically upregulated, along with *Keap1*. However, there were no changes in apoptosis-related gene expression, and *ATG7* was downregulated (Figure 5D).

### 3.5 2*H*-1,4-Benzoxazin-3(4*H*)-one linked 1,2,3-triazole derivatives induced DNA damage and autophagy

**FIGURE 6:**
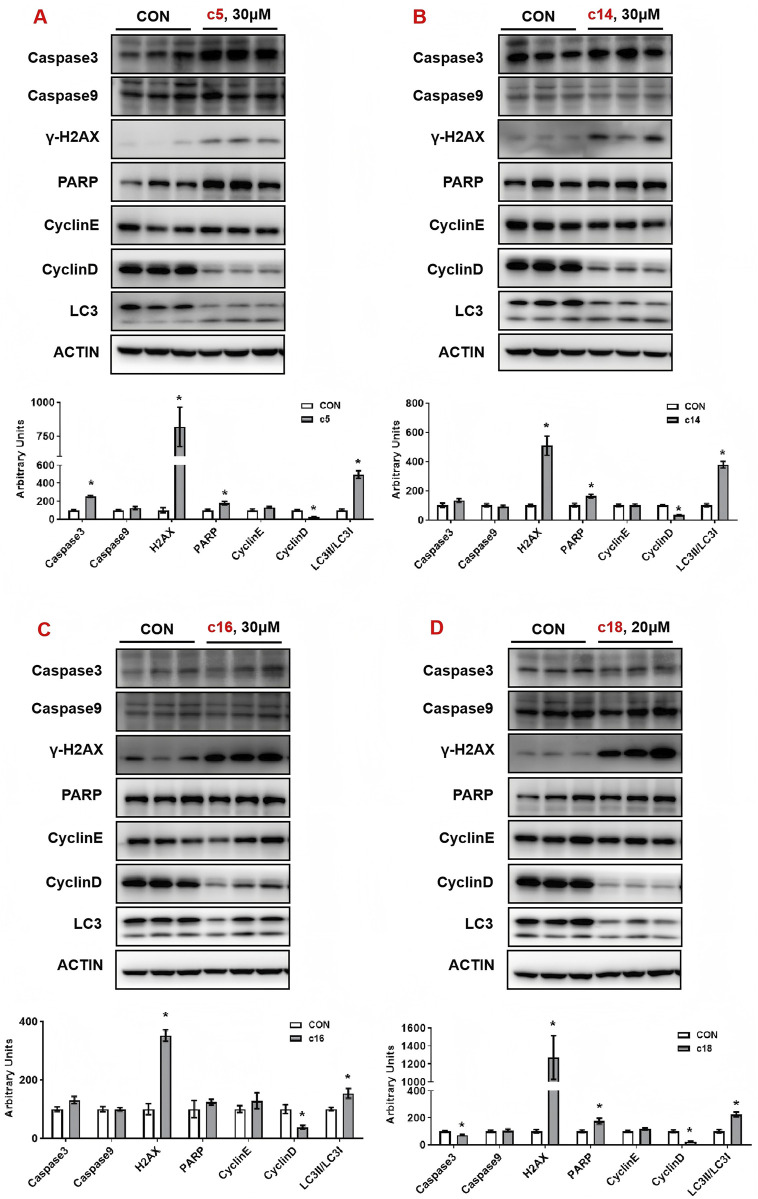
Compounds **c5**, **c14**, **c16**, and **c18** affected DNA damage and autophagy. **(A–D)** Huh-7 cells were treated with different concentrations of compounds **c5**, **c14**, **c16**, and **c18** (20 or 30 μM) for 24 h. Protein expressions were analyzed by Western blot. Top: Western blot; bottom: quantitative measurements relative to ACTIN. Data were presented as means ± SEM; *P < 0.05.

To further examine the regulatory effects of 2*H*-1,4-benzoxazin-3(4*H*)-one linked 1,2,3-triazole derivatives on protein expression, Huh-7 cells were treated with compounds **c5**, **c14**, **c16**, and **c18** for 72 h, after which whole-cell protein lysates were collected for analysis. In **c5**-treated cells, the apoptosis marker *caspase-3* was upregulated, along with *H2AX* and *PARP*, indicating DNA damage. *LC3*, a key autophagy marker, was significantly induced, while *cyclin D*, a critical regulator of the cell cycle, was downregulated. Similar effects were observed in cells treated with **c14**, **c16**, and **c18**, with upregulation of *H2AX*, *PARP*, and *LC3*, and downregulation of *CyclinD* (Figures 6A–D).

### 3.6 2*H*-1,4-Benzoxazin-3(4*H*)-one linked 1,2,3-triazole derivatives stimulated DNA damage of tumor cells

**FIGURE 7:**
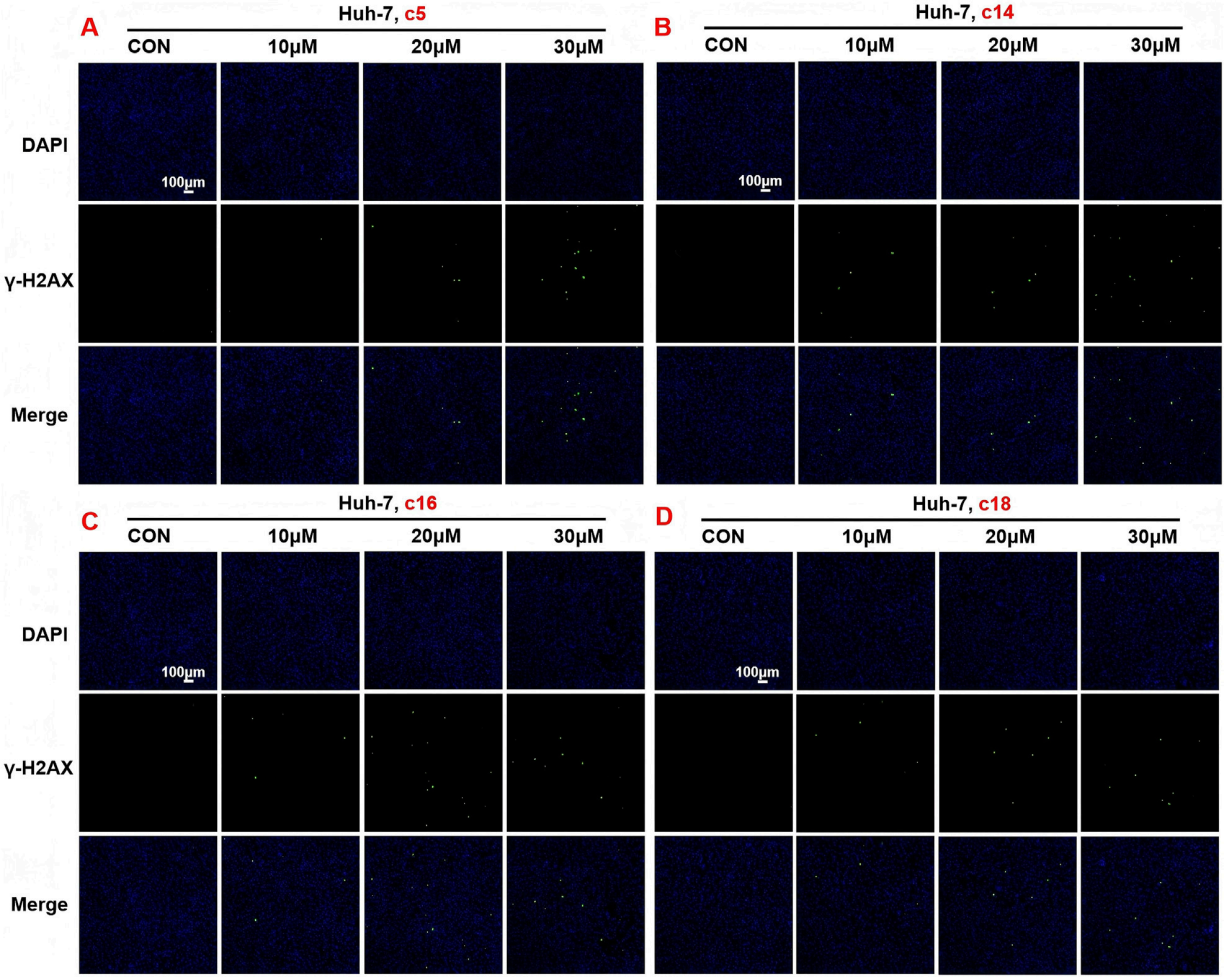
Compounds **c5**, **c14**, **c16**, and **c18** affected DNA damage. **(A–D)** DNA damage detection shown by γ-H2AX staining in Huh-7 cells treated with compounds **c5**, **c14**, **c16**, and **c18** (10 μM, 20 μM, or 30 μM).

In order to further verify the effects of 2*H*-1,4-benzoxazin-3(4*H*)-one linked 1,2,3-triazole derivatives on DNA damage, Huh-7 cells were treated with different concentrations (10 μM, 20 μM, or 30 μM) of compounds **c5**, **c14**, **c16**, and **c18**. After 24 h of treatment, cells were detected with the DNA damage assay kit and observed under a fluorescence microscope. Consistent with the protein expression data, results showed that 2*H*-1,4-benzoxazin-3(4*H*)-one linked 1,2,3-triazole derivatives could significantly induce cell DNA damage, and this may contribute to cell death (Figures 7A–D).

### 3.7 Effects of 2*H*-1,4-benzoxazin-3(4*H*)-one linked 1,2,3-triazole derivatives on autophagy

**FIGURE 8:**
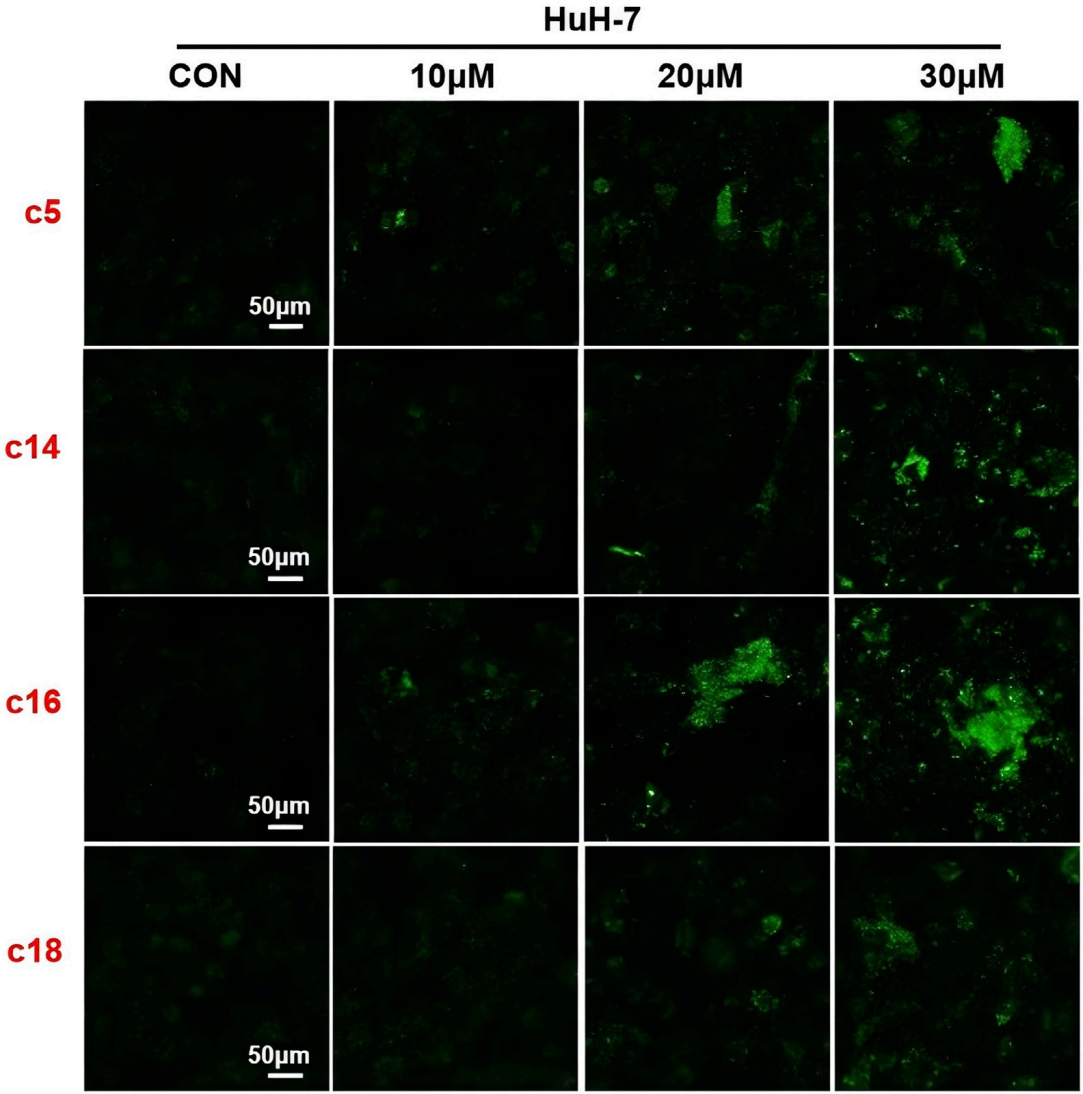
Compounds **c5**, **c14**, **c16**, and **c18** affected autophagy. Autophagy staining in Huh-7 cells treated with compounds **c5**, **c14**, **c16**, and **c18** (10 μM, 20 μM, or 30 μM).

To further evaluate the role of 2*H*-1,4-benzoxazin-3(4*H*)-one linked 1,2,3-triazole derivatives in regulating autophagy, Huh-7 cells were treated with different doses (10 μM, 20 μM, or 30 μM) of compounds **c5**, **c14**, **c16**, and **c18** for 24 h, and the autophagosome was detected by the MDC (monodansylcadaverine) probe. MDC could specifically label autophagosomes, which will show as green, and we can directly observe them using a fluorescence microscope. The results showed that after treatment with compounds **c5**, **c14**, **c16**, and **c18**, autophagosomes in cells increased, especially at doses lower than 30 μM (Figure 8), which was consistent with the increased protein expression of LC3. This indicated that 2*H*-1,4-benzoxazin-3(4*H*)-one linked 1,2,3-triazole derivatives could induce cell autophagy progression.

### 3.8 Identification of the binding mode of compound c18 with DNA in human topoisomerase II beta

**FIGURE 9:**
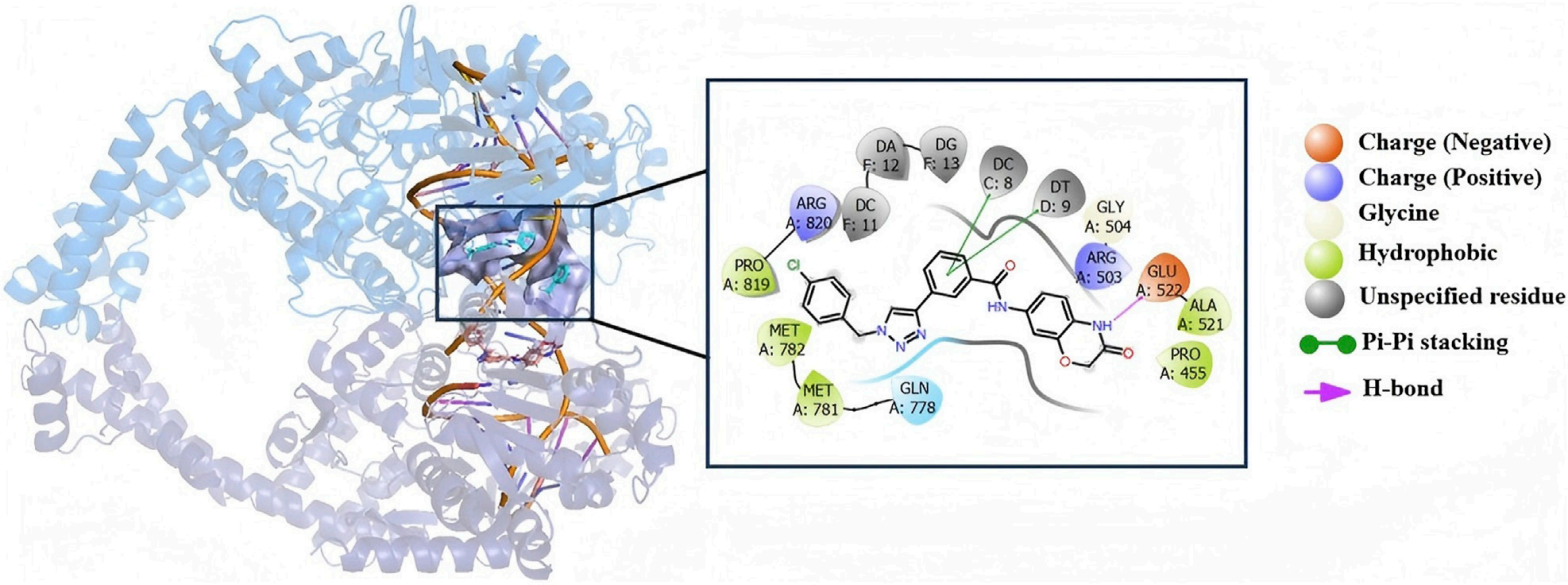
The binding models and interaction profiles between compound **c18** and DNA within human topoisomerase II.

Molecular docking was performed to analyze the binding interactions of compound **c18** with DNA in the human topoisomerase II beta complex. As shown in Figure 9, the benzene ring of compound **c18** forms two π–π stacking interactions with DC C:8 and DT D:9, respectively, while its 2-oxazolidinone moiety establishes a hydrogen bond with Glu522. These interactions imply that compound **c18** may exert its biological effects, at least in part, by targeting the topoisomerase II–DNA complex.

### 3.9 HE staining of organs after c18 treatment

**FIGURE 10:**
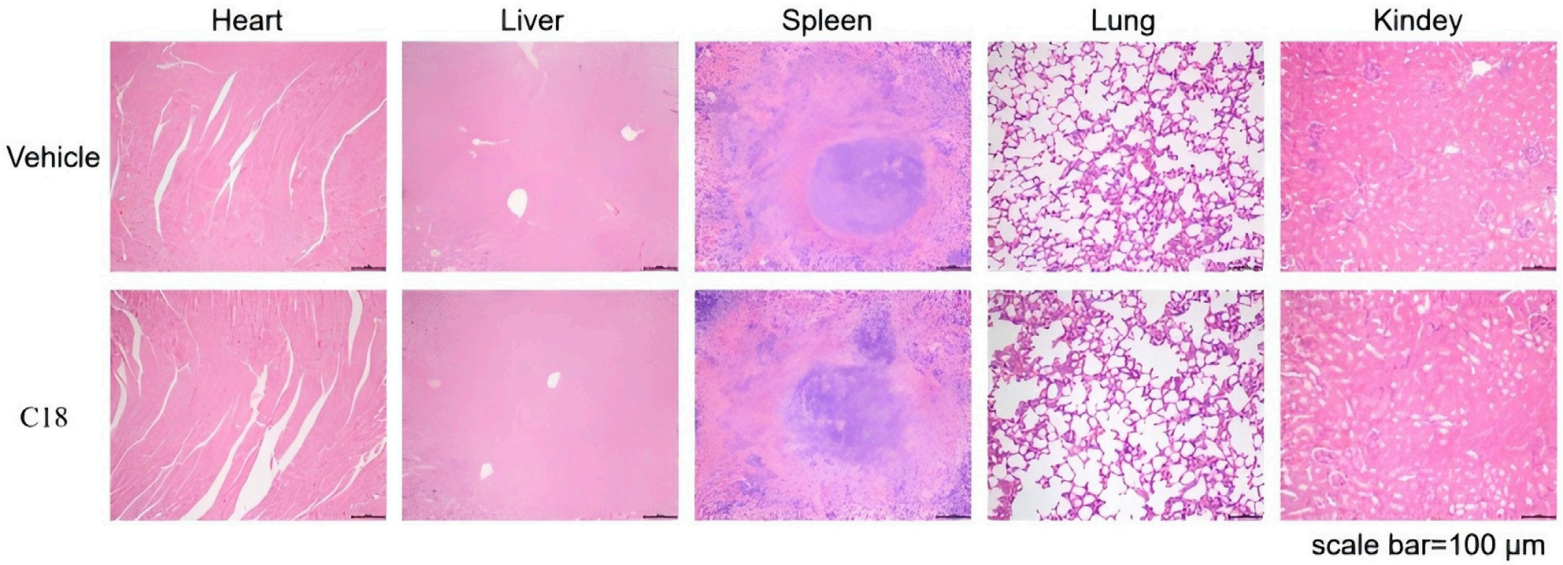
H&E staining was performed on various organs of mice treated with compound **c18**.

We assessed the safety of compound **c18** using an acute toxicity test in KM mice. Male mice were divided into a control group and a **c18**-treated group, with the latter receiving 500 mg/kg of compound **c18** via gavage for 14 consecutive days, while the control group received an equivalent volume of solvent. To evaluate the safety of compound **c18**, major organs—including the heart, liver, spleen, lungs, and kidneys—were collected from treated KM mice and subjected to hematoxylin and eosin (HE) staining. As shown in Figure 10, HE staining revealed no significant histopathological changes in the **c18**-treated group compared to the control group, indicating that compound **c18** did not cause observable tissue damage. These results suggest that compound **c18** possesses a favorable safety profile at the tested dose.

## 4 Conclusion

In this study, we explored the anti-tumor effects of 2*H*-1,4-benzoxazin-3(4*H*)-one linked 1,2,3-triazole derivatives, focusing on their potential to induce DNA damage and regulate apoptosis and autophagy in human hepatocellular carcinoma (Huh-7) cells. The compounds exhibited significant anti-proliferative activity, particularly against Huh-7 cells, with minimal toxicity observed in normal liver L02 cells. The IC_50_ values for compounds **c5**, **c14**, **c16**, and **c18** suggest that these derivatives hold promise as therapeutic agents for liver cancer treatment. A key finding of this study is the role of the rigid molecular structure of these compounds in their ability to induce DNA damage in tumor cells. The 2*H*-1,4-benzoxazin-3(4*H*)-one linked 1,2,3-triazole derivatives, characterized by their rigid planar structures, efficiently promote DNA damage, as evidenced by the upregulation of DNA damage markers such as H2AX and the significant increase in DNA fragmentation. This rigid structure likely contributes to their ability to interact with DNA or related cellular machinery, enhancing the induction of DNA damage, which in turn triggers apoptotic pathways in the cancer cells. In addition to DNA damage, the compounds also induced apoptosis in a dose-dependent manner, with flow cytometry analysis confirming the increase in apoptotic cells. The anti-proliferative effects were further supported by live/dead cell staining, which revealed a dose-dependent increase in dead cells. Moreover, the compounds significantly upregulated genes related to oxidative stress, apoptosis (such as caspase-7), and autophagy (such as LC3), demonstrating their multi-faceted mechanisms of action. These findings suggest that the rigid, planar structure of the 2*H*-1,4-benzoxazin-3(4*H*)-one linked 1,2,3-triazole derivatives is a crucial factor in their ability to induce DNA damage and activate apoptosis and autophagy pathways in tumor cells. Given their promising efficacy in targeting liver cancer cells and their relatively low toxicity to normal cells, these compounds represent a promising class of anticancer agents. Further studies are needed to elucidate the precise molecular mechanisms and to evaluate the therapeutic potential of these compounds in broader cancer models.

## 5 Experimental

### 5.1 Materials and chemistry

The 2H-1,4-benzoxazin-3(4H)-one-linked 1,2,3-triazole derivatives used in the manuscript were synthesized previously (Hou et al., 2025). Dimethyl sulfoxide (DMSO) was obtained from Sigma-Aldrich (St. Louis, Missouri, United States). Dulbecco’s modified Eagle medium (DMEM), RPMI 1640 Medium, Fetal bovine serum (FBS), and penicillin/streptomycin were purchased from Gibco (Grand Island, NY, United States). The Enhanced Cell Counting Kit-8, Calcein/PI Live/Dead Viability Assay kit, DNA Damage Detection kit, and Autophagy Staining Assay kit were obtained from Beyotime Biotechnology (Shanghai, China). The Annexin V-FITC/propidium iodide (PI) staining kits were provided by BD Biosciences (Franklin Lake, New Jersey, United States).

#### 5.1.1 General synthetic procedure for compounds c1–c20

Compound **a** (0.02 mol), 3-ethynylbenzoic acid (0.03 mol), HATU (0.03 mol), DIPEA (0.06 mol), and DMF were added to the reaction flask at 20 °C–25 °C and stirred for 24 h. The reaction was monitored by TLC. After 24 h, the solution turned light brown. DMF was removed under reduced pressure, and the residue was extracted with dichloromethane (150 mL × 3). The organic extracts were combined and washed with saturated sodium chloride (150 mL × 2) to pH 7, yielding a viscous brownish–yellow liquid upon vacuum distillation. Methanol was added dropwise under ultrasonic vibration, resulting in precipitation. The solid was filtered and dried, and compound **b** was obtained (Hou et al., 2025).

In a reaction flask, compound **b** (3 mmol), substituted azide (3.6 mmol), TERT-butanol (70 mL), water (70 mL), tetrahydrofuran (70 mL), anhydrous copper sulfate (0.6 mmol), and sodium ascorbate (1 mmol) were added, and the mixture was stirred and refluxed at 70 °C–80 °C for 5 h. After completion, the reaction was extracted with dichloromethane (100 mL × 3), combined, and washed with saturated sodium chloride (100 mL × 2). The organic phase was further washed with brine (100 mL × 2), dried over sodium sulfate, and concentrated under reduced pressure to obtain the crude product. Recrystallization from ethyl acetate yielded the desired compound, which was pure enough for further characterization and anti-tumor studies (Hou et al., 2025).

The spectroscopic characterization of compounds **c1–c20** is provided as follows.

3-(1-(2,6-dichlorobenzyl)-1*H*-1,2,3-triazol-4-yl)-*N*-(3-oxo-3,4-dihydro-2*H*-benzo[*b*][1,4]oxazin-7-yl)benzamide (compound **c1)**: White solid, HR-MS(ESI): Calcd. C24H18Cl2N5O3 [M + H]^+^ *m/z*: 494.0787, found: 494.0775. ^1^H NMR (400 MHz, DMSO-d_6_): 10.67 (s, 1H), 10.27 (s, 1H), 8.66 (s, 1H), 8.38 (s, 1H), 8.07 (d, *J* = 8.0 Hz, 1H), 7.87 (d, *J* = 8.0 Hz, 1H), 7.62–7.56 (m, 3H), 7.52–7.45 (m, 2H), 7.38–7.35 (m, 1H), 6.88 (d, *J* = 8.0 Hz, 1H), 5.88 (s, 2H), 4.57 (s, 2H). ^13^C NMR (100 MHz, DMSO-d_6_): 165.6, 164.9, 146.0, 143.5, 136.5, 136.1, 135.0, 132.1, 131.2, 130.6, 129.5, 129.4, 129.3, 128.6, 127.5, 124.9, 123.6, 122.5, 116.0, 114.8, 109.1, 67.3, 49.3.

3-(1-(3-chlorobenzyl)-1*H*-1,2,3-triazol-4-yl)-*N*-(3-oxo-3,4-dihydro-2*H*-benzo[*b*][1,4]oxazin-7-yl)benzamide (compound **c2)**: White solid, ^1^H NMR (400 MHz, DMSO-d_6_): 10.66 (s, 1H), 10.28 (s, 1H), 8.77 (s, 1H), 8.39 (s, 1H), 8.06 (d, *J* = 4.0 Hz, 1H), 7.88 (d, *J* = 8.0 Hz, 1H), 7.60 (t, *J* = 8.0 Hz, 1H), 7.50–7.48 (m, 2H), 7.44–7.33 (m, 4H), 6.88 (d, *J* = 8.0 Hz, 1H), 5.71 (s, 2H), 4.58 (s, 2H).^13^C NMR (100 MHz, DMSO-d_6_): 165.6, 164.9, 146.6, 143.5, 138.7, 136.1, 135.0, 133.8, 131.2, 129.5, 128.7, 128.5, 128.3, 127.5, 127.1, 124.8, 123.6, 122.6, 116.0, 114.8, 109.1, 67.3, 52.8, 49.0. HR-MS(ESI): Calcd. C24H19ClN5O3 [M + H]^+^ *m/z*: 460.1176, found: 460.1162.

3-(1-(2-fluorobenzyl)-1*H*-1,2,3-triazol-4-yl)-*N*-(3-oxo-3,4-dihydro-2*H*-benzo[*b*][1,4]oxazin-7-yl)benzamide (compound **c3)**: White solid, ^1^H NMR (400 MHz, DMSO-d_6_): 10.67 (s, 1H), 10.27 (s, 1H), 8.72 (s, 1H), 8.39 (s, 1H), 8.06 (d, *J* = 4.0 Hz, 1H), 7.88 (d, *J* = 8.0 Hz, 1H), 7.59 (t, *J* = 8.0 Hz, 1H), 7.50–7.26 (m, 6H), 6.88 (d, *J* = 8.0 Hz, 1H), 5.74 (s, 2H), 4.58 (s, 2H).^13^C NMR (100 MHz, DMSO-d_6_): 165.6, 164.9, 161.8, 159.4, 146.5, 143.5, 136.1, 135.0, 131.3, 129.5, 128.5, 127.5, 125.4, 124.8, 123.6, 123.2, 123.0, 122.6, 116.2, 116.0, 114.9, 109.1, 67.3, 47.6. HR-MS(ESI): Calcd. C24H19FN5O3 [M + H]^+^ *m/z*: 444.1472, found: 444.1463.

3-(1-(4-iodobenzyl)-1*H*-1,2,3-triazol-4-yl)-*N*-(3-oxo-3,4-dihydro-2*H*-benzo[*b*][1,4]oxazin-7-yl)benzamide (compound **c4)**: White solid, ^1^H NMR (400 MHz, DMSO-d_6_): 10.70 (s, 1H), 10.30 (s, 1H), 8.73 (s, 1H), 8.37 (s, 1H), 8.05 (d, *J* = 4.0 Hz, 1H), 7.88 (d, *J* = 8.0 Hz, 1H), 7.78 (d, *J* = 8.0 Hz, 2H), 7.60 (t, *J* = 8.0 Hz, 1H), 7.49 (s, 1H), 7.26 (dd, *J*_*1*_ = 4.0 Hz, *J*_*2*_ = 4.0 Hz, 1H), 7.18 (d, *J* = 4.0 Hz, 2H), 6.87 (d, *J* = 8.0 Hz, 1H), 5.65 (s, 2H), 4.58 (s, 2H).^13^C NMR (100 MHz, DMSO-d_6_): 165.5, 165.0, 146.6, 143.5, 138.0, 136.1, 136.1, 135.0, 131.2, 130.7, 129.5, 128.5, 127.5, 124.8, 123.6, 122.5, 114.8, 109.1, 95.0, 67.2, 52.9. HR-MS(ESI): Calcd. C24H19IN5O3 [M + H]^+^ *m/z*: 552.0533, found: 552.0515.

3-(1-(4-bromobenzyl)-1*H*-1,2,3-triazol-4-yl)-*N*-(3-oxo-3,4-dihydro-2*H*-benzo[*b*][1,4]oxazin-7-yl)benzamide (compound **c5)**: White solid, ^1^H NMR (400 MHz, DMSO-d_6_): 10.67 (s, 1H), 10.28 (s, 1H), 8.71 (s, 1H), 8.36 (s, 1H), 8.04 (d, *J* = 8.0 Hz, 1H), 7.88 (d, *J* = 8.0 Hz, 1H), 7.60 (d, *J* = 8.0 Hz, 2H), 7.48 (s, 1H), 7.37–7.33 (m, 3H), 6.88 (d, J = 8.0 Hz, 1H), 5.66 (s, 2H), 4.57 (s, 2H). ^13^C NMR (100 MHz, DMSO-d_6_): 165.6, 165.0, 146.6, 143.5, 136.0, 135.7, 134.9, 132.2, 131.2, 130.7, 129.5, 128.5, 127.5, 124.8, 123.6, 122.5, 121.9, 116.0, 114.9, 109.2, 67.2, 52.8. HR-MS(ESI): Calcd. C24H19BrN5O3 [M + H]^+^ *m/z*: 504.0671, found: 504.0665.

3-(1-(3,5-dimethylbenzyl)-1*H*-1,2,3-triazol-4-yl)-*N*-(3-oxo-3,4-dihydro-2*H*-benzo[*b*][1,4]oxazin-7-yl)benzamide (compound **c6)**: White solid, ^1^H NMR (400 MHz, DMSO-d_6_): 10.67 (s, 1H), 10.28 (s, 1H), 8.71 (s, 1H), 8.39 (s, 1H), 8.06 (d, J = 8.0 Hz, 1H), 7.88 (d, J = 8.0 Hz, 1H), 7.60 (t, J = 4.0 Hz, 2H), 7.50 (s, 1H), 7.38 (dd, J_1_ = 4.0 Hz, J_2_ = 4.0 Hz, 1H), 6.99 (s, 3H), 6.88 (d, J = 8.0 Hz, 1H), 5.58 (s, 2H), 4.58 (s, 2H), 2.26 (s, 6H).^13^C NMR (100 MHz, DMSO-d_6_): 165.6, 164.9, 146.5, 143.5, 138.4, 136.1, 136.1, 135.0, 131.3, 130.0, 129.5, 128.5, 127.4, 124.8, 123.6, 122.4, 116.0, 114.9, 109.1, 67.3, 53.6, 21.3. HR-MS(ESI): Calcd. C26H24N5O3 [M + H]^+^ *m/z*: 454.1879, found: 454.1867.

3-(1-(2-cyanobenzyl)-1*H*-1,2,3-triazol-4-yl)-*N*-(3-oxo-3,4-dihydro-2*H*-benzo[*b*][1,4]oxazin-7-yl)benzamide (compound **c7)**: White solid, ^1^H NMR (400 MHz, DMSO-d_6_): 10.67 (s, 1H), 10.29 (s, 1H), 8.75 (s, 1H), 8.38 (s, 1H), 8.06 (d, J = 8.0 Hz, 1H), 7.94 (d, J = 8.0 Hz, 1H), 7.89 (d, J = 8.0 Hz, 1H), 7.76 (t, J = 8.0 Hz, 1H), 7.63–7.59 (m, 2H), 7.50–7.48 (m, 2H), 7.36 (dd, J_1_ = 4.0 Hz, J_2_ = 4.0 Hz, 1H), 6.88 (d, J = 8.0 Hz, 1H), 5.90 (s, 2H), 4.58 (s, 2H). ^13^C NMR (100 MHz, DMSO-d_6_): 165.6, 165.0, 146.5, 143.5, 138.9, 136.1, 135.0, 134.3, 133.9, 131.1, 130.1, 129.8, 129.5, 128.6, 127.5, 124.9, 123.6, 122.9, 117.4, 116.0, 114.9, 111.7, 109.2, 67.2, 51.8. HR-MS(ESI): Calcd. C25H19N6O3 [M + H]^+^ *m/z*: 451.1519, found: 451.1504.

*N*-(3-oxo-3,4-dihydro-2*H*-benzo[*b*][1,4]oxazin-7-yl)-3-(1-(3-(trifluoromethyl)benzyl)-1*H*-1,2,3-triazol-4-yl)benzamide (compound **c8)**: White solid, ^1^H NMR (400 MHz, DMSO-d_6_): 10.62 (s, 1H), 10.24 (s, 1H), 8.70 (s, 1H), 8.31 (s, 1H), 7.99 (d, J = 8.0 Hz, 1H), 7.82 (d, J = 8.0 Hz, 1H), 7.71–7.66 (m, 2H), 7.60–7.53 (m, 3H), 7.42 (s, 1H), 7.29 (d, J = 8.0 Hz, 1H), 6.84 (d, J = 8.0 Hz, 1H), 5.74 (s, 2H), 4.58 (s, 2H). ^13^C NMR (100 MHz, DMSO-d_6_): 165.7, 165.1, 146.6, 143.5, 137.6, 136.0, 134.9, 132.6, 131.1, 130.5, 130.1, 129.8, 129.6, 128.6, 127.5, 125.8, 125.5, 125.5, 125.0, 125.0, 124.8, 123.6, 123.0, 122.6, 116.0, 115.0, 109.2, 67.2, 52.8. HR-MS(ESI): Calcd. C25H19F5N5O3 [M + H]^+^ *m/z*: 494.1440, found: 494.1433.

3-(1-(3-chloro-4-fluorobenzyl)-1*H*-1,2,3-triazol-4-yl)-*N*-(3-oxo-3,4-dihydro-2*H*-benzo[*b*][1,4]oxazin-7-yl)benzamide (compound **c9)**: White solid, ^1^H NMR (400 MHz, DMSO-d_6_): 10.68 (s, 1H), 10.29 (s, 1H), 8.75 (s, 1H), 8.38 (s, 1H), 8.06 (d, J = 4.0 Hz, 1H), 7.89 (d, J = 8.0 Hz, 1H), 7.69–7.59 (m, 2H), 7.50–7.36 (m, 4H), 6.89 (d, J = 8.0 Hz, 1H), 5.70 (s, 2H), 4.58 (s, 2H). ^13^C NMR (100 MHz, DMSO-d_6_): 165.6, 165.0, 158.7, 146.6, 143.5, 136.1, 135.0, 134.1, 134.1, 131.2, 131.0, 130.9, 129.7, 129.6, 129.5, 129.5, 129.5, 128.5, 127.5, 124.8, 123.6, 122.5, 117.9, 117.7, 116.0, 114.9, 109.2, 67.2, 52.6, 52.2. HR-MS(ESI): Calcd. C24H18ClFN5O3 [M + H]^+^ *m/z*: 478.1082, found: 478.1073.

3-(1-(2,6-difluorobenzyl)-1*H*-1,2,3-triazol-4-yl)-*N*-(3-oxo-3,4-dihydro-2*H*-benzo[*b*][1,4]oxazin-7-yl)benzamide (compound **c10)**: White solid, ^1^H NMR (400 MHz, DMSO-d_6_): 10.66 (s, 1H), 10.26 (s, 1H), 8.71 (s, 1H), 8.38 (s, 1H), 8.06 (d, J = 4.0 Hz, 1H), 7.87 (d, J = 8.0 Hz, 1H), 7.61–7.49 (m, 3H), 7.38 (d, J = 4.0 Hz, 1H), 7.21 (t, J = 8.0 Hz, 2H), 6.88 (d, J = 8.0 Hz, 1H), 5.74 (s, 2H), 4.58 (s, 2H). ^13^C NMR (100 MHz, DMSO-d_6_): 165.6, 164.9, 146.4, 143.5, 136.1, 132.2, 131.1, 129.4, 128.5, 127.5, 124.9, 123.6, 122.5, 116.0, 114.9, 112.5, 112.3, 109.1, 67.3. HR-MS(ESI): Calcd. C24H18F2N5O3 [M + H]^+^ *m/z*: 462.1378, found: 462.1370.

3-(1-(2-cyano-5-fluorobenzyl)-1*H*-1,2,3-triazol-4-yl)-*N*-(3-oxo-3,4-dihydro-2*H*-benzo[*b*][1,4]oxazin-7-yl)benzamide (compound **c11)**: White solid, ^1^H NMR (400 MHz, DMSO-d_6_): 10.66 (s, 1H), 10.29 (s, 1H), 8.77 (s, 1H), 8.38 (s, 1H), 8.07–8.04 (m, 1H), 7.89 (d, J = 8.0 Hz, 1H), 7.61 (t, J = 8.0 Hz, 1H), 7.49–7.35 (m, 4H), 6.88 (d, J = 8.0 Hz, 1H), 5.90 (s, 2H), 4.58 (s, 2H). ^13^C NMR (100 MHz, DMSO-d_6_): 166.2, 165.6, 164.9, 163.7, 146.5, 143.5, 142.4, 142.3, 137.0, 136.9, 136.1, 135.0, 131.1, 129.5, 128.6, 127.6, 124.9, 123.6, 123.0, 118.0, 117.7, 117.5, 117.2, 116.7, 116.0, 114.9, 109.1, 108.5, 108.4, 67.3, 51.5. HR-MS(ESI): Calcd. C25H18FN6O3 [M + H]^+^ *m/z*: 469.1424, found: 469.1410.

3-(1-(2-bromobenzyl)-1*H*-1,2,3-triazol-4-yl)-*N*-(3-oxo-3,4-dihydro-2*H*-benzo[*b*][1,4]oxazin-7-yl)benzamide (compound **c12)**: White solid, ^1^H NMR (400 MHz, DMSO-d_6_): 10.67 (s, 1H), 10.28 (s, 1H), 8.69 (s, 1H), 8.39 (s, 1H), 8.06 (d, J = 4.0 Hz, 1H), 7.88 (d, J = 4.0 Hz, 1H), 7.72 (d, J = 8.0 Hz, 1H), 7.60 (t, J = 8.0 Hz, 1H), 7.49–7.43 (m, 2H), 7.38–7.27 (m, 3H), 6.88 (d, J = 8.0 Hz, 1H), 5.77 (s, 2H), 4.58 (s, 2H). ^13^C NMR (100 MHz, DMSO-d_6_): 165.6, 164.9, 146.4, 143.5, 136.1, 135.1, 135.0, 133.4, 131.2, 131.1, 130.9, 129.5, 128.8, 128.5, 127.5, 123.6, 123.4, 122.8, 116.0, 114.9, 109.1, 67.3, 53.7. HR-MS(ESI): Calcd. C24H19BrN5O3 [M + H]^+^ *m/z*: 504.0671, found: 504.0666.

3-(1-(4-bromo-2-fluorobenzyl)-1*H*-1,2,3-triazol-4-yl)-*N*-(3-oxo-3,4-dihydro-2*H*-benzo[*b*][1,4]oxazin-7-yl)benzamide (compound **c13)**: White solid, ^1^H NMR (400 MHz, DMSO-d_6_): 10.67 (s, 1H), 10.27 (s, 1H), 8.71 (s, 1H), 8.38 (s, 1H), 8.06 (d, J = 4.0 Hz, 1H), 7.88 (d, J = 8.0 Hz, 1H), 7.63–7.36 (m, 8H), 6.88 (d, J = 8.0 Hz, 1H), 5.72 (s, 2H), 4.58 (s, 2H). ^13^C NMR (100 MHz, DMSO-d_6_): 165.6, 164.9, 146.5, 143.5, 136.1, 135.0, 133.0, 132.9, 132.8, 131.2, 129.5, 128.6, 128.5, 128.3, 127.5, 124.8, 123.6, 122.8, 122.6, 119.7, 119.5, 116.0, 114.9, 109.1, 67.3, 47.7, 47.2. HR-MS(ESI): Calcd. C24H18BrFN5O3 [M + H]^+^ *m/z*: 522.0577, found: 522.0572.

3-(1-(3-fluorobenzyl)-1*H*-1,2,3-triazol-4-yl)-*N*-(3-oxo-3,4-dihydro-2*H*-benzo[*b*][1,4]oxazin-7-yl)benzamide (compound **c14)**: White solid, ^1^H NMR (400 MHz, DMSO-d_6_): 10.67 (s, 1H), 10.28 (s, 1H), 8.76 (s, 1H), 8.39 (s, 1H), 8.06 (d, J = 8.0 Hz, 1H), 7.88 (d, J = 8.0 Hz, 1H), 7.60 (d, J = 8.0 Hz, 1H), 7.50–7.45 (m, 2H), 7.38 (dd, J1 = 4.0 Hz, J2 = 4.0 Hz, 1H), 7.25–7.17 (m, 3H), 6.88 (d, J = 8.0 Hz, 1H), 5.72 (s, 2H), 4.58 (s, 2H). ^13^C NMR (100 MHz, DMSO-d_6_): 165.6, 164.9, 163.8, 161.4, 146.6, 143.5, 139.0, 138.9, 135.0, 131.4, 131.3, 131.2, 129.5, 128.5, 127.5, 124.8, 124.5, 124.5, 123.6, 122.6, 116.0, 115.4, 115.2, 114.9, 109.1, 67.3, 52.9. HR-MS(ESI): Calcd. C24H19FN5O3 [M + H]^+^ *m/z*: 444.1472, found: 444.1463.

3-(1-(2-chloro-6-fluorobenzyl)-1*H*-1,2,3-triazol-4-yl)-*N*-(3-oxo-3,4-dihydro-2*H*-benzo[*b*][1,4]oxazin-7-yl)benzamide (compound **c15)**: White solid, ^1^H NMR (400 MHz, DMSO-d_6_): 10.66 (s, 1H), 10.26 (s, 1H), 8.69 (s, 1H), 8.37 (s, 1H), 8.06 (d, J = 8.0 Hz, 1H), 7.87 (d, J = 8.0 Hz, 1H), 7.61–7.35 (m, 5H), 6.88 (d, J = 8.0 Hz, 1H), 5.79 (s, 2H), 4.57 (s, 2H). ^13^C NMR (100 MHz, DMSO-d_6_): 165.6, 164.9, 146.2, 143.5, 136.1, 135.0, 132.4, 132.3, 131.1, 129.4, 128.6, 127.5, 126.4, 124.9, 123.6, 122.6, 116.0, 115.6, 115.3, 114.9, 109.1, 67.2, 45.1. HR-MS(ESI): Calcd. C24H18ClFN5O3 [M + H]^+^ *m/z*: 478.1082, found: 478.1069.

*N*-(3-oxo-3,4-dihydro-2*H*-benzo[*b*][1,4]oxazin-7-yl)-3-(1-(4-(trifluoromethyl)benzyl)-1*H*-1,2,3-triazol-4-yl)benzamide (compound **c16)**: White solid, ^1^H NMR (400 MHz, DMSO-d_6_): 10.67 (s, 1H), 10.28 (s, 1H), 8.78 (s, 1H), 8.39 (s, 1H), 8.06 (d, J = 8.0 Hz, 1H), 7.89 (d, J = 8.0 Hz, 1H), 7.79–7.76 (m, 3H), 7.61–7.59 (m, 4H), 7.50 (s, 1H), 7.37 (d, J = 8.0 Hz, 1H), 5.82 (s, 2H), 4.58 (s, 2H). ^13^C NMR (100 MHz, DMSO-d_6_): 165.5, 164.9, 146.7, 143.5, 141.0, 136.1, 135.0, 131.2, 129.5, 129.4, 129.4, 129.1, 128.5, 127.5, 126.2, 126.2, 126.0, 126.0, 124.8, 123.6, 122.8, 116.0, 114.9, 109.1, 67.3, 53.2, 52.9. HR-MS(ESI): Calcd. C25H19F3N5O3 [M + H]^+^ *m/z*: 494.1440, found: 494.1418.

3-(1-(3-bromobenzyl)-1*H*-1,2,3-triazol-4-yl)-*N*-(3-oxo-3,4-dihydro-2*H*-benzo[*b*][1,4]oxazin-7-yl)benzamide (compound **c17)**: White solid, ^1^H NMR (400 MHz, DMSO-d_6_): 10.67 (s, 1H), 10.28 (s, 1H), 8.76 (s, 1H), 8.39 (s, 1H), 8.07–8.04 (m, 1H), 7.90–7.87 (m, 1H), 7.62–7.56 (m, 3H), 7.50 (s, 4H), 7.38–7.37 (m, 3H), 6.88 (d, J = 8.0 Hz, 1H), 5.70 (s, 2H), 4.58 (s, 2H). ^13^C NMR (100 MHz, DMSO-d_6_): 165.6, 164.9, 146.6, 143.5, 138.9, 136.1, 135.0, 131.6, 131.5, 131.2, 129.5, 128.5, 127.5, 127.5, 124.8, 123.6, 122.67, 122.3, 116.0, 114.8, 109.1, 67.3, 52.7, 49.0. HR-MS(ESI): Calcd. C24H19BrN5O3 [M + H]^+^ *m/z*: 504.0671, found: 504.0657.

3-(1-(4-chlorobenzyl)-1*H*-1,2,3-triazol-4-yl)-*N*-(3-oxo-3,4-dihydro-2*H*-benzo[*b*][1,4]oxazin-7-yl)benzamide (compound **c18)**: White solid, ^1^H NMR (400 MHz, DMSO-d_6_): 10.67 (s, 1H), 10.27 (s, 1H), 8.73 (s, 1H), 8.38 (s, 1H), 8.05 (d, J = 8.0 Hz, 1H), 7.88 (d, J = 8.0 Hz, 1H), 7.60 (t, J = 8.0 Hz, 1H), 7.49–7.46 (m, 3H), 7.42–7.35 (m, 3H), 6.88 (d, J = 8.0 Hz, 1H), 5.69 (s, 2H), 4.58 (s, 2H). ^13^C NMR (100 MHz, DMSO-d_6_): 165.6, 164.9, 146.6, 143.5, 136.1, 135.3, 135.0, 133.4, 131.2, 130.4, 129.5, 129.3, 128.5, 127.5, 124.8, 123.6, 122.5, 116.0, 114.9, 109.1, 67.3, 52.8. HR-MS(ESI): Calcd. C24H19ClN5O3 [M + H]^+^ *m/z*: 460.1176, found: 460.1152.

3-(1-(3-iodobenzyl)-1*H*-1,2,3-triazol-4-yl)-*N*-(3-oxo-3,4-dihydro-2*H*-benzo[*b*][1,4]oxazin-7-yl)benzamide (compound **c19)**: White solid, ^1^H NMR (400 MHz, DMSO-d_6_): 10.67 (s, 1H), 10.28 (s, 1H), 8.76 (s, 1H), 8.38 (s, 1H), 8.05 (d, J = 8.0 Hz, 1H), 7.88 (d, J = 8.0 Hz, 1H), 7.79 (s, 1H), 7.41 (d, J = 8.0 Hz, 1H), 7.60 (t, J = 8.0 Hz, 1H), 7.50 (s, 1H), 7.38 (t, J = 4.0 Hz, 2H), 7.21 (t, J = 4.0 Hz, 2H), 6.88 (d, J = 8.0 Hz, 1H), 5.66 (s, 2H), 4.58 (s, 2H). ^13^C NMR (100 MHz, DMSO-d_6_): 165.6, 164.9, 146.6, 143.5, 138.8, 137.4, 137.0, 136.1, 135.0, 131.4, 131.2, 129.5, 128.5, 127.9, 127.5, 124.8, 123.6, 122.6, 116.0, 114.8, 109.1, 95.5, 67.3, 52.6. HR-MS(ESI): Calcd. C24H19IN5O3 [M + H]^+^ *m/z*: 552.0533, found: 552.0517.

3-(1-(2-methylbenzyl)-1*H*-1,2,3-triazol-4-yl)-*N*-(3-oxo-3,4-dihydro-2*H*-benzo[*b*][1,4]oxazin-7-yl)benzamide (compound **c20)**: White solid, ^1^H NMR (400 MHz, DMSO-d_6_): 10.66 (s, 1H), 10.26 (s, 1H), 8.63 (s, 1H), 8.37 (s, 1H), 8.06 (d, J = 4.0 Hz, 1H), 7.86 (d, J = 8.0 Hz, 1H), 7.58 (t, J = 8.0 Hz, 1H), 7.48 (s, 1H), 7.35 (d, J = 8.0 Hz, 1H), 7.27–7.16 (m, 4H), 6.87 (d, J = 8.0 Hz, 1H), 5.68 (s, 2H), 4.57 (s, 2H). ^13^C NMR (100 MHz, DMSO-d_6_): 165.6, 164.9, 146.4, 143.5, 136.8, 136.1, 135.0, 134.4, 131.3, 130.9, 130.9, 129.4, 129.2, 128.8, 128.5, 127.4, 126.8, 124.8, 123.6, 122.4, 116.0, 114.8, 109.1, 67.3, 51.7, 19.1. HR-MS(ESI): Calcd. C25H22N5O3 [M + H]^+^ *m/z*: 440.1723, found: 440.1714.

### 5.2 Biological study

#### 5.2.1 Cell culture

Human tumor cell lines A549 (lung cancer), Huh7 (hepatocellular carcinoma), MCF-7 (breast cancer), HCT-116 (colon cancer), and SKOV3 (ovarian cancer) were all obtained from ATCC. Cells were cultured in DMEM or RPMI 1640 medium containing 10% FBS and 1% penicillin/streptomycin at 37 °C in a 5% CO_2_-humidified atmosphere.

#### 5.2.2 CCK-8 assay and IC_50_ measurement

Cells were cultured in DMEM or RPMI 1640 medium with 10% FBS and 1% penicillin/streptomycin at 37 °C and 5% CO_2_. A CCK-8 assay was performed with several human cancer cell lines, including A549, Huh-7, MCF-7, HCT-116, and SKOV3, according to the instructions. Cells were plated at a density of 1,000–3,000 cells per well in a 96-well plate. After 24-h incubation, cells were treated with 40 μM compounds for 48 h or 50 μM compounds for 72 h. Cell viability was measured by adding 10 μL per well of CCK-8 reagent, which was dissolved in the cell culture medium, for 1 hour. Absorbance was taken at 450 nm using a microplate reader. The IC_50_ values of the compounds were further determined. Cells were seeded in a 96-well plate at a density of 1 × 10^3^ cells/well. After adhering overnight, cells treated with five concentrations (10 μM, 20 μM, 40 µM, or 80 μM) were used to draw the dose–response curve, and IC_50_ values were calculated by GraphPad Prism software. The CCK-8 assay was performed in triplicate, and each experiment was repeated at least three times. The results were expressed as mean ± SEM, and P < 0.05 was considered statistically significant.

#### 5.2.3 Live/dead viability assay

A live and dead viability assay was performed using the Calcein/PI Live/Dead Viability Assay Kit. Cells were plated at a density of 2 × 10^3^ cells per well in a 96-well plate to adhere overnight. Then, compounds were added with different concentrations for 24 h. The medium was replaced with cell culture medium containing Calcein AM and propidium iodide for 30 min at 37 °C in the dark. Cells were then observed and photographed by confocal microscopy.

#### 5.2.4 Apoptosis quantification

Cells were plated at a density of 3 × 10^5^ cells per well in a 6-well plate. Different concentrations of compounds were added to cells for 72 h. Then, cells were collected and washed with PBS. Annexin V-FITC and propidium iodide were then added to stained cells for 20 min. Apoptotic cells were quantified by a flow cytometer, and raw data were further analyzed by Flow Jo software v10. Values were expressed as mean ± SEM. Statistical significance was assessed using one-way ANOVA followed by the Student–Newman–Keuls test. P < 0.05 was considered statistically significant. Data were visualized using GraphPad Prism.

#### 5.2.5 Cellular RT-PCR level measurement

Huh-7 cells were seeded in a 12-well plate and treated with compounds for 72 h. Total RNA was extracted using TRIzol reagent (Ambion, United States), and RT-PCR was performed using PowerUp™ SYBR™ Green Master Mix (ABI, United States). Gene expression was normalized to GAPDH. Primers were obtained from Aiji Biotech (Guangzhou, China) and are listed in Table 3. Values were expressed as mean ± SEM. Statistical significance was assessed using a two-tailed Student’s t-test. P < 0.05 was considered statistically significant.

**TABLE 3:** List of oligonucleotide primer pairs used in RT-PCR analysis.

Gene	Forward primer (5′–3′)	Reverse primer (5′–3′)
P53	CCT​CAG​CAT​CTT​ATC​CGA​GTG​G	TGG​ATG​GTG​GTA​CAG​TCA​GAG​C
P21	AGG​TGG​ACC​TGG​AGA​CTC​TCA​G	TCC​TCT​TGG​AGA​AGA​TCA​GCC​G
H2AX	CGG​CAG​TGC​TGG​AGT​ACC​TCA	AGC​TCC​TCG​TCG​TTG​CGG​ATG
Bax	TCA​GGA​TGC​GTC​CAC​CAA​GAA​G	TGT​GTC​CAC​GGC​GGC​AAT​CAT​C
Bcl2	ATC​GCC​CTG​TGG​ATG​ACT​GAG​T	GCC​AGG​AGA​AAT​CAA​ACA​GAG​GC
Caspase3	GGA​AGC​GAA​TCA​ATG​GAC​TCT​GG	GCA​TCG​ACA​TCT​GTA​CCA​GAC​C
Caspase6	AGG​TGG​ATG​CAG​CCT​CCG​TTT​A	ATG​AGC​CGT​TCA​CAG​TTT​CCC​G
Caspase7	CGG​AAC​AGA​CAA​AGA​TGC​CGA​G	AGG​CGG​CAT​TTG​TAT​GGT​CCT​C
Keap1	CAA​CTT​CGC​TGA​GCA​GAT​TGG​C	TGA​TGA​GGG​TCA​CCA​GTT​GGC​A
Nrf2	AGG​TTG​CCC​ACA​TTC​CCA​AA	ACG​TAG​CCG​AAG​AAA​CCT​CA
Gpx4	TTGGTCGGCTGGACGAGG	GGGACGCGCACATGGT
Tnfa	ACT​GAA​AGC​ATG​ATC​CGG​GAC​G	AGC​AGG​CAG​AAG​AGC​GTG​GTG​G
ATG5	GGA​TGG​GAT​TGC​AAA​ATG​ACA​GA	TCC​TAG​TGT​GTG​CAA​CTG​TCC
ATG7	GGC​CAA​TAA​GAT​GGG​TCT​GA	GCT​TTT​GTC​CAC​TGC​TCC​TC

#### 5.2.6 Western blot analysis

Antibodies used in Western blot were as follows: caspase3, caspase9, LC3, γ-H2AX, PARP, cyclin D, cyclin E, β-catenin (all from Cell Signaling Technology, United States), and β-actin (Sigma, United States). Cells were treated with compounds (0 μM, 5 μM, 10 μM, 20 μM, or 30 μM) for 72 h, and the whole-cell proteins were collected using RIPA buffer containing protease/phosphatase inhibitor cocktail. The protein expressions were measured and analyzed using Western blot. Values were expressed as mean ± SEM. Statistical significance was assessed using a two-tailed Student’s t-test. P < 0.05 was considered statistically significant. Data were visualized using GraphPad Prism.

#### 5.2.7 DNA damage staining

Huh-7 cells with a density of 2 × 10^3^ cells/well were seeded in 96-well plates. Different doses (0 μM, 5 μM, 10 μM, 20 μM, or 30 μM) of the compounds were added to cells for 24 h. A DNA Damage Detection Kit was used to dye the cells. The cell nucleus was stained blue, and DNA-damaged cells were stained green. The cells were observed and photographed by a fluorescent microscope.

#### 5.2.8 Autophagy staining assay

Huh-7 cells were seeded in a 96-well plate at a density of 2 × 10^3^ cells/well. Cells were treated with compounds at the doses of 0 μM, 5 μM, 10 μM, 20 μM, or 30 μM. After 24 h of treatment, the medium was removed, and cells were stained with monodansylcadaverine for 1 h in the dark at 37 °C. The cells were photographed with confocal microscopy.

#### 5.2.9 Molecular docking

The X-ray crystal structure of human topoisomerase II beta (PDB ID: 4G0U) (Wu et al., 2013) was retrieved from the Protein Data Bank (https://www.rcsb.org), featuring a resolution of 2.70 Å. The Protein Preparation Wizard in Maestro was employed to prepare the initial structure of human topoisomerase II beta. The chemical structure of compound **1** was optimized using LigPrep, which performed functions such as 2D to 3D conversion, charge assignment, group orientation adjustment, chirality correction, etc. Subsequently, the OPLS4 force field was applied to obtain stable and low-energy conformations. The docking region was defined using a grid box automatically generated by the Receptor Grid Generation tool, based on the binding pocket in DNA (Richard et al., 2004). Finally, the compound **c18** was docked into the prepared grid of the protein targets to identify the binding model of compound **c18** with the DNA using the standard precision (SP) docking mode of Glide.

#### 5.2.10 Statistical analyses

Data were presented as means ± SEM and visualized using Graph Prim 7.0. A two-tailed Student’s t-test or one-way ANOVA followed by a Student–Newman–Keuls (SNK) test was used to assess significant differences. *P* < 0.05 was considered statistically significant.

## Data Availability

The datasets presented in this study can be found in online repositories. The names of the repository/repositories and accession number(s) can be found in the article/Supplementary Material.
