# Supplementary material for: 2H-1,4-Benzoxazin-3(4H)-one linked 1,2,3-triazole derivatives and their study on inducing DNA damage in tumor cells
Source: Front Pharmacol. 2025 Aug 15;16:1564090. doi: 10.3389/fphar.2025.1564090 (PMC12394750; doi:10.3389/fphar.2025.1564090)

2H-1,4-Benzoxazin-3(4H)-one Linked 1,2,3-Triazole Derivatives and Their Study on Inducing DNA Damage in Tumor Cells

Xixi Hou^a^, Yajie Guo^b*^, Xi Wang^c^, En Gao^c*^, Jianxue Yang^a*^

^a^The First Affiliated Hospital, and College of Clinical Medicine of Henan University of Science and Technology, Luoyang, 471003, P. R. China

^b^Department of Emergency, The Eighth Affiliated Hospital, Sun Yat-Sen University, Shenzhen 518033, P. R. China

^c^School of Chemistry and Chemical Engineering, Henan Normal University, Xinxiang, 453007, China.

^d^State Key Laboratory of Quality Research in Chinese Medicine/Macau Institute for Applied Research in Medicine and Health, Macau University of Science and Technology, Macau, China

*Corresponding authors:

En Gao, School of Chemistry and Chemical Engineering, Henan Normal University, Xinxiang, China. E-mail: [ge_163@126.com](mailto:ge_163@126.com)

**Jianxue Yang**, The First Affiliated Hospital, and College of Clinical Medicine of Henan University of Science and Technology, Henan 453007, P. R. China. E-mail: Docyjx@126.com

Compound c1: HR-MS(ESI): Calcd. C24H18Cl2N5O3 [M+H]^+^ *m/z*: 494.0787, found: 494.0775. ^1^H NMR(400MHz, DMSO-d_6_): 10.67 (s, 1H), 10.27 (s, 1H), 8.66 (s, 1H), 8.38 (s, 1H), 8.07 (d, J=8.0Hz, 1H), 7.87 (d, J=8.0Hz, 1H), 7.62-7.56 (m, 3H), 7.52-7.45 (m, 2H), 7.38-7.35 (m, 1H), 6.88 (d, J=8.0Hz, 1H), 5.88 (s, 2H), 4.57 (s, 2H). ^13^C NMR(100MHz, DMSO-d_6_): 165.66, 164.96, 146.09, 143.50, 136.53, 136.18, 135.04, 132.18, 131.20, 130.63, 129.50, 129.43, 129.35, 128.60, 127.51, 124.93, 123.62, 122.56, 116.00, 114.89, 109.17, 67.30, 49.38

Compound c2: HR-MS(ESI): Calcd. C24H19ClN5O3 [M+H]^+^ *m/z*: 460.1176, found: 460.1162. ^1^H NMR(400MHz, DMSO-d_6_): 10.66 (s, 1H), 10.28 (s, 1H), 8.77 (s, 1H), 8.39 (s, 1H), 8.06 (d, J=4.0Hz, 1H), 7.88 (d, J=8.0Hz, 1H), 7.60 (t, J1=8.0Hz, J2=8.0Hz, 1H), 7.50-7.48 (m, 2H), 7.44-7.33 (m, 4H), 6.88 (d, J=8.0Hz, 1H), 5.71 (s, 2H), 4.58 (s, 2H).^13^C NMR(100MHz, DMSO-d_6_): 165.60, 164.97, 146.65, 143.52, 138.75, 136.16, 135.03, 133.84, 131.25, 129.52, 128.70, 128.54, 128.38, 127.54, 127.19, 124.88, 123.63, 122.67, 116.01, 114.89, 109.18, 67.31, 52.83, 49.07

Compound c3: HR-MS(ESI): Calcd. C24H19FN5O3 [M+H]^+^ *m/z*: 444.1472, found: 444.1463. ^1^H NMR(400MHz, DMSO-d_6_): 10.67 (s, 1H), 10.27 (s, 1H), 8.72 (s, 1H), 8.39 (s, 1H), 8.06 (d, J=4.0Hz, 1H), 7.88 (d, J=8.0Hz, 1H), 7.59 (t, J1=8.0Hz, J2=8.0Hz, 1H), 7.50-7.26 (m, 6H), 6.88 (d, J=8.0Hz, 1H), 5.74 (s, 2H), 4.58 (s, 2H).^13^C NMR(100MHz, DMSO-d_6_): 165.60, 164.97, 161.86, 159.41, 146.50, 143.51, 136.15, 135.03, 131.30, 129.50, 128.56, 127.54, 125.40, 124.87, 123.63, 123.24, 123.09, 122.60, 116.26, 116.05, 114.90, 109.19, 67.31, 47.69

Compound c4: HR-MS(ESI): Calcd. C24H19IN5O3 [M+H]^+^ *m/z*: 552.0533, found: 552.0515. ^1^H NMR(400MHz, DMSO-d_6_): 10.70 (s, 1H), 10.30 (s, 1H), 8.73 (s, 1H), 8.37 (s, 1H), 8.05 (d, J=4.0Hz, 1H), 7.88 (d, J=8.0Hz, 1H), 7.78 (d, J=8.0Hz, 2H), 7.60 (t, J1=8.0Hz, J2=8.0Hz, 1H), 7.49 (s, 1H), 7.26 (dd, J1=4.0Hz, J2=4.0Hz, 1H), 7.18 (d, J=4.0Hz, 2H), 6.87 (d, J=8.0Hz, 1H), 5.65 (s, 2H), 4.58 (s, 2H).^13^C NMR(100MHz, DMSO-d_6_): 165.59, 165.01, 146.60, 143.50, 138.09, 136.15, 136.12, 135.01, 131.25, 130.74, 129.54, 128.52, 127.53, 124.81, 123.61, 122.59, 114.87, 109.15, 95.04, 67.28, 52.99

Compound c5: HR-MS(ESI): Calcd. C24H19BrN5O3 [M+H]^+^ *m/z*: 504.0671, found: 504.0665. ^1^H NMR(400MHz, DMSO-d_6_): 10.67 (s, 1H), 10.28 (s, 1H), 8.71 (s, 1H), 8.36 (s, 1H), 8.04 (d, J=8.0Hz, 1H), 7.88 (d, J=8.0Hz, 1H), 7.60 (d, J=8.0Hz, 2H), 7.48 (s, 1H), 7.37-7.33 (m, 3H), 6.88 (d, J=8.0Hz, 1H), 5.66 (s, 2H), 4.57 (s, 2H).^13^C NMR(100MHz, DMSO-d_6_): 165.67, 165.04, 146.62, 143.52, 136.08, 135.73, 134.98, 132.23, 131.22, 130.71, 129.58, 128.57, 127.53, 124.83, 123.61, 122.56, 121.99, 116.05, 114.95, 109.22, 67.28, 52.87

Compound c6: HR-MS(ESI): Calcd. C26H24N5O3 [M+H]^+^ *m/z*: 454.1879, found: 454.1867. ^1^H NMR(400MHz, DMSO-d_6_): 10.67 (s, 1H), 10.28 (s, 1H), 8.71 (s, 1H), 8.39 (s, 1H), 8.06 (d, J=8.0Hz, 1H), 7.88 (d, J=8.0Hz, 1H), 7.60 (t, J=4.0Hz, 2H), 7.50 (s, 1H), 7.38 (dd, J1=4.0Hz, J2=4.0Hz, 1H), 6.99 (s, 3H), 6.88 (d, J=8.0Hz, 1H), 5.58 (s, 2H), 4.58 (s, 2H), 2.26 (s, 6H).^13^C NMR(100MHz, DMSO-d_6_): 165.61, 164.97, 146.54, 143.51, 138.43, 136.18, 136.12, 135.03, 131.37, 130.07, 129.50, 128.51, 127.47, 124.83, 123.63, 122.44, 116.01, 114.90, 109.19, 67.31, 53.61, 21.30

Compound c7: HR-MS(ESI): Calcd. C25H19N6O3 [M+H]^+^ *m/z*: 451.1519, found: 451.1504. ^1^H NMR(400MHz, DMSO-d_6_): 10.67 (s, 1H), 10.29 (s, 1H), 8.75 (s, 1H), 8.38 (s, 1H), 8.06 (d, J=8.0Hz, 1H), 7.94 (d, J=8.0Hz, 1H), 7.89 (d, J=8.0Hz, 1H), 7.76 (t, J=8.0Hz, 1H), 7.63-7.59 (m, 2H), 7.50-7.48 (m, 2H), 7.36 (dd, J1=4.0Hz, J2=4.0Hz, 1H), 6.88 (d, J=8.0Hz, 1H), 5.90 (s, 2H), 4.58 (s, 2H). ^13^C NMR(100MHz, DMSO-d_6_): 165.64, 165.01, 146.54, 143.52, 138.99, 136.15, 135.01, 134.37, 133.92, 131.16, 130.10, 129.80, 129.56, 128.61, 127.59, 124.90, 123.63, 122.98, 117.43, 116.03, 114.93, 111.78, 109.21, 67.29, 51.83.

Compound c8: HR-MS(ESI): Calcd. C25H19F5N5O3 [M+H]^+^ *m/z*: 494.1440, found: 494.1433 ^1^H NMR(400MHz, DMSO-d_6_): 10.62 (s, 1H), 10.24 (s, 1H), 8.70 (s, 1H), 8.31 (s, 1H), 7.99 (d, J=8.0Hz, 1H), 7.82 (d, J=8.0Hz, 1H), 7.71-7.66 (m, 2H), 7.60-7.53 (m, 3H), 7.42 (s, 1H), 7.29 (d, J=8.0Hz, 1H), 6.84 (d, J=8.0Hz, 1H), 5.74 (s, 2H), 4.58 (s, 2H). ^13^C NMR(100MHz, DMSO-d_6_): 165.75, 165.11, 146.67, 143.52, 137.65, 136.05, 134.95, 132.62, 131.13, 130.52, 130.13, 129.81, 129.63, 128.62, 127.57, 125.80, 125.55, 125.51, 125.09, 125.05, 124.84, 123.60, 123.09, 122.69, 116.09, 115.00, 109.25, 67.25, 52.89.

Compound c9: HR-MS(ESI): Calcd. C24H18ClFN5O3 [M+H]^+^ *m/z*: 478.1082, found: 478.1073. ^1^H NMR(400MHz, DMSO-d_6_): 10.68 (s, 1H), 10.29 (s, 1H), 8.75 (s, 1H), 8.38 (s, 1H), 8.06 (d, J=4.0Hz, 1H), 7.89 (d, J=8.0Hz, 1H), 7.69-7.59 (m, 2H), 7.50-7.36 (m, 4H), 6.89 (d, J=8.0Hz, 1H), 5.70 (s, 2H), 4.58 (s, 2H). ^13^C NMR(100MHz, DMSO-d_6_): 165.65, 165.01, 158.77, 146.65, 143.52, 136.12, 135.01, 134.14, 134.10, 131.21, 131.03, 130.95, 129.71, 129.64, 129.59, 129.54, 129.51, 128.56, 127.54, 124.87, 123.62, 122.57, 117.92, 117.71, 116.03, 114.92, 109.20, 67.29, 52.60, 52.25.

Compound c10: HR-MS(ESI): Calcd. C24H18F2N5O3 [M+H]^+^ *m/z*: 462.1378, found: 462.1370. ^1^H NMR(400MHz, DMSO-d_6_): 10.66 (s, 1H), 10.26 (s, 1H), 8.71 (s, 1H), 8.38 (s, 1H), 8.06 (d, J=4.0Hz, 1H), 7.87 (d, J=8.0Hz, 1H), 7.61-7.49 (m, 3H), 7.38 (d, J=4.0Hz, 1H), 7.21 (t, J=8.0Hz, 2H), 6.88 (d, J=8.0Hz, 1H), 5.74 (s, 2H), 4.58 (s, 2H). ^13^C NMR(100MHz, DMSO-d_6_): 165.63, 164.97, 146.40, 143.51, 136.16, 132.27, 131.18, 129.47, 128.59, 127.55, 124.90, 123.63, 122.54, 116.01, 114.90, 112.58, 112.34, 109.19, 67.30.

Compound c11: HR-MS(ESI): Calcd. C25H18FN6O3 [M+H]^+^ *m/z*: 469.1424, found: 469.1410. ^1^H NMR(400MHz, DMSO-d_6_): 10.66 (s, 1H), 10.29 (s, 1H), 8.77 (s, 1H), 8.38 (s, 1H), 8.07-8.04 (m, 1H), 7.89 (d, J=8.0Hz, 1H), 7.61 (t, J=8.0Hz, 1H), 7.49-7.35 (m, 4H), 6.88 (d, J=8.0Hz, 1H), 5.90 (s, 2H), 4.58 (s, 2H). ^13^C NMR(100MHz, DMSO-d_6_): 166.26, 165.61, 164.98, 163.72, 146.57, 143.51, 142.47, 142.38, 137.01, 136.91, 136.18, 135.02, 131.13, 129.54, 128.61, 127.60, 124.95, 123.63, 123.06, 118.03, 117.79, 117.50, 117.27, 116.74, 116.02, 114.90, 109.18, 108.51, 108.48, 67.30, 51.50.

Compound c12: HR-MS(ESI): Calcd. C24H19BrN5O3 [M+H]^+^ *m/z*: 504.0671, found: 504.0666. ^1^H NMR(400MHz, DMSO-d_6_): 10.67 (s, 1H), 10.28 (s, 1H), 8.69 (s, 1H), 8.39 (s, 1H), 8.06 (d, J=4.0Hz, 1H), 7.88 (d, J=4.0Hz, 1H), 7.72 (d, J=8.0Hz, 1H), 7.60 (t, J=8.0Hz, 1H), 7.49-7.43 (m, 2H), 7.38-7.27 (m, 3H), 6.88 (d, J=8.0Hz, 1H), 5.77 (s, 2H), 4.58 (s, 2H). ^13^C NMR(100MHz, DMSO-d_6_): 165.62, 164.98, 146.40, 143.51, 136.15, 135.15, 135.02, 133.45, 131.26, 131.13, 130.99, 129.52, 128.83, 128.59, 127.54, 123.63, 123.45, 122.86, 116.01, 114.90, 109.19, 67.30, 53.71.

Compound c13: HR-MS(ESI): Calcd. C24H18BrFN5O3 [M+H]^+^ *m/z*: 522.0577, found: 522.0572. ^1^H NMR(400MHz, DMSO-d_6_): 10.67 (s, 1H), 10.27 (s, 1H), 8.71 (s, 1H), 8.38 (s, 1H), 8.06 (d, J=4.0Hz, 1H), 7.88 (d, J=8.0Hz, 1H), 7.63-7.36 (m, 8H), 6.88 (d, J=8.0Hz, 1H), 5.72 (s, 2H), 4.58 (s, 2H). ^13^C NMR(100MHz, DMSO-d_6_): 165.60, 164.97, 146.53, 143.51, 136.16, 135.03, 133.04, 132.91, 132.87, 131.21, 129.50, 128.61, 128.51, 128.38, 127.55, 124.88, 123.63, 122.82, 122.63, 119.75, 119.51, 116.01, 114.90, 109.19, 67.31, 47.77, 47.28.

Compound c14: HR-MS(ESI): Calcd. C24H19FN5O3 [M+H]^+^ *m/z*: 444.1472, found: 444.1463. ^1^H NMR(400MHz, DMSO-d_6_): 10.67 (s, 1H), 10.28 (s, 1H), 8.76 (s, 1H), 8.39 (s, 1H), 8.06 (d, J=8.0Hz, 1H), 7.88 (d, J=8.0Hz, 1H), 7.60 (d, J=8.0Hz, 1H), 7.50-7.45 (m, 2H), 7.38 (dd, J1=4.0Hz, J2=4.0Hz, 1H), 7.25-7.17 (m, 3H), 6.88 (d, J=8.0Hz, 1H), 5.72 (s, 2H), 4.58 (s, 2H). ^13^C NMR(100MHz, DMSO-d_6_): 165.61, 164.98, 163.89, 161.46, 146.65, 143.52, 139.06, 138.98, 135.03, 131.44, 131.36, 131.28, 129.51, 128.55, 127.53, 124.88, 124.56, 124.53, 123.63, 122.66, 116.01, 115.47, 115.26, 114.90, 109.18, 67.31, 52.92.

Compound c15: HR-MS(ESI): Calcd. C24H18ClFN5O3 [M+H]^+^ *m/z*: 478.1082, found: 478.1069. ^1^H NMR(400MHz, DMSO-d_6_): 10.66 (s, 1H), 10.26 (s, 1H), 8.69 (s, 1H), 8.37 (s, 1H), 8.06 (d, J=8.0Hz, 1H), 7.87 (d, J=8.0Hz, 1H), 7.61-7.35 (m, 5H), 6.88 (d, J=8.0Hz, 1H), 5.79 (s, 2H), 4.57 (s, 2H). ^13^C NMR(100MHz, DMSO-d_6_): 165.66, 164.99, 146.21, 143.51, 136.15, 135.02, 132.44, 132.33, 131.18, 129.48, 128.61, 127.52, 126.41, 124.90, 123.62, 122.63, 116.01, 115.61, 115.39, 114.90, 109.18, 67.29, 45.17.

Compound c16:HR-MS(ESI): Calcd. C25H19F3N5O3 [M+H]^+^ *m/z*: 494.1440, found: 494.1418. ^1^H NMR(400MHz, DMSO-d_6_): 10.67 (s, 1H), 10.28 (s, 1H), 8.78 (s, 1H), 8.39 (s, 1H), 8.06 (d, J=8.0Hz, 1H), 7.89 (d, J=8.0Hz, 1H), 7.79-7.76 (m, 3H), 7.61-7.59 (m, 4H), 7.50 (s, 1H), 7.37 (d, J=8.0Hz, 1H), 5.82 (s, 2H), 4.58 (s, 2H). ^13^C NMR(100MHz, DMSO-d_6_): 165.58, 164.97, 146.70, 143.51, 141.04, 136.15, 135.03, 131.25, 129.52, 129.43, 129.40, 129.14, 128.55, 127.55, 126.24, 126.20, 126.06, 126.02, 124.88, 123.64, 122.81, 116.01, 114.90, 109.18, 67.30, 53.29, 52.94.

Compound c17: HR-MS(ESI): Calcd. C24H19BrN5O3 [M+H]^+^ *m/z*: 504.0671, found: 504.0657. ^1^H NMR(400MHz, DMSO-d_6_): 10.67 (s, 1H), 10.28 (s, 1H), 8.76 (s, 1H), 8.39 (s, 1H), 8.07-8.04 (m, 1H), 7.90-7.87 (m, 1H), 7.62-7.56 (m, 3H), 7.50 (s, 4H), 7.38-7.37 (m, 3H), 6.88 (d, J=8.0Hz, 1H), 5.70 (s, 2H), 4.58 (s, 2H). ^13^C NMR(100MHz, DMSO-d_6_): 165.61, 164.98, 146.64, 143.51, 138.98, 136.15, 135.03, 131.61, 131.52, 131.24, 129.53, 128.55, 127.58, 127.54, 124.87, 123.63, 122.67, 122.38, 116.01, 114.89, 109.18, 67.30, 52.76, 49.07.

Compound c18: HR-MS(ESI): Calcd. C24H19ClN5O3 [M+H]^+^ *m/z*: 460.1176, found: 460.1152. ^1^H NMR(400MHz, DMSO-d_6_): 10.67 (s, 1H), 10.27 (s, 1H), 8.73 (s, 1H), 8.38 (s, 1H), 8.05 (d, J=8.0Hz, 1H), 7.88 (d, J=8.0Hz, 1H), 7.60 (t, J=8.0Hz, 1H), 7.49-7.46 (m, 3H), 7.42-7.35 (m, 3H), 6.88 (d, J=8.0Hz, 1H), 5.69 (s, 2H), 4.58 (s, 2H). ^13^C NMR(100MHz, DMSO-d_6_): 165.60, 164.98, 146.63, 143.51, 136.14, 135.35, 135.02, 133.44, 131.28, 130.40, 129.52, 129.30, 128.53, 127.52, 124.85, 123.63, 122.56, 116.01, 114.90, 109.19, 67.30, 52.80.

Compound c19: HR-MS(ESI): Calcd. C24H19IN5O3 [M+H]^+^ *m/z*: 552.0533, found: 552.0517. ^1^H NMR(400MHz, DMSO-d_6_): 10.67 (s, 1H), 10.28 (s, 1H), 8.76 (s, 1H), 8.38 (s, 1H), 8.05 (d, J=8.0Hz, 1H), 7.88 (d, J=8.0Hz, 1H), 7.79 (s, 1H), 7.41 (d, J=8.0Hz, 1H), 7.60 (t, J=8.0Hz, 1H), 7.50 (s, 1H), 7.38 (t, J=4.0Hz, 2H), 7.21 (t, J=4.0Hz, 2H), 6.88 (d, J=8.0Hz, 1H), 5.66 (s, 2H), 4.58 (s, 2H). ^13^C NMR(100MHz, DMSO-d_6_): 165.60, 164.97, 146.63, 143.51, 138.86, 137.45, 137.03, 136.15, 135.03, 131.47, 131.27, 129.53, 128.55, 127.94, 127.55, 124.87, 123.63, 122.64, 116.01, 114.89, 109.18, 95.54, 67.31, 52.68.

Compound c20: HR-MS(ESI): Calcd. C25H22N5O3 [M+H]^+^ *m/z*: 440.1723, found: 440.1714. ^1^H NMR(400MHz, DMSO-d_6_): 10.66 (s, 1H), 10.26 (s, 1H), 8.63 (s, 1H), 8.37 (s, 1H), 8.06 (d, J=4.0Hz, 1H), 7.86 (d, J=8.0Hz, 1H), 7.58 (t, J=8.0Hz, 1H), 7.48 (s, 1H), 7.35 (d, J=8.0Hz, 1H), 7.27-7.16 (m, 4H), 6.87 (d, J=8.0Hz, 1H), 5.68 (s, 2H), 4.57 (s, 2H). ^13^C NMR(100MHz, DMSO-d_6_): 165.62, 164.97, 146.46, 143.51, 136.87, 136.14, 135.03, 134.44, 131.34, 130.97, 130.96, 129.49, 129.28, 128.89, 128.53, 127.48, 126.81, 124.83, 123.63, 122.49, 116.00, 114.89, 109.17, 67.30, 51.74, 19.15.

c1


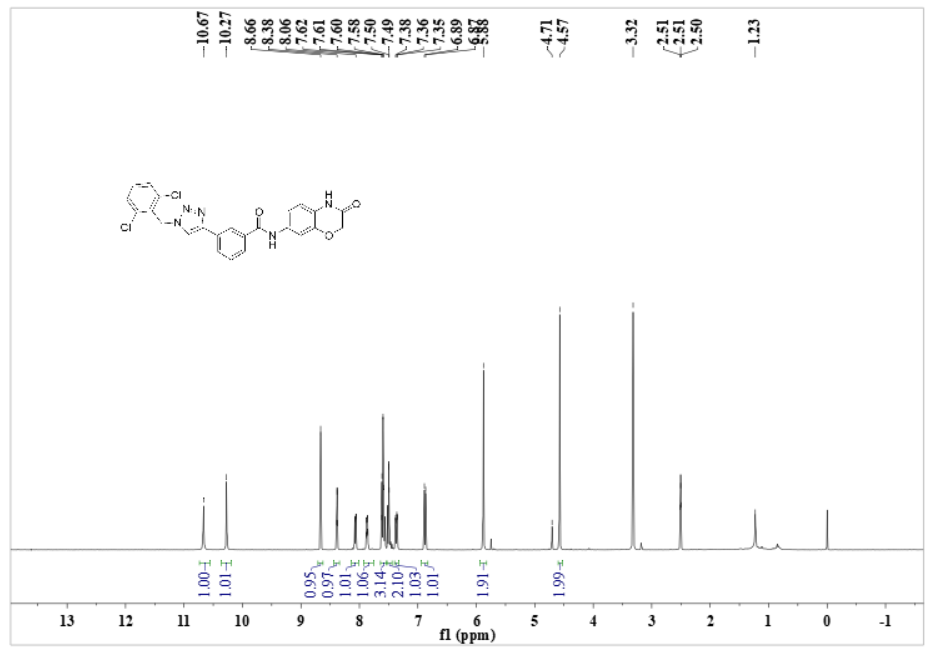


c2


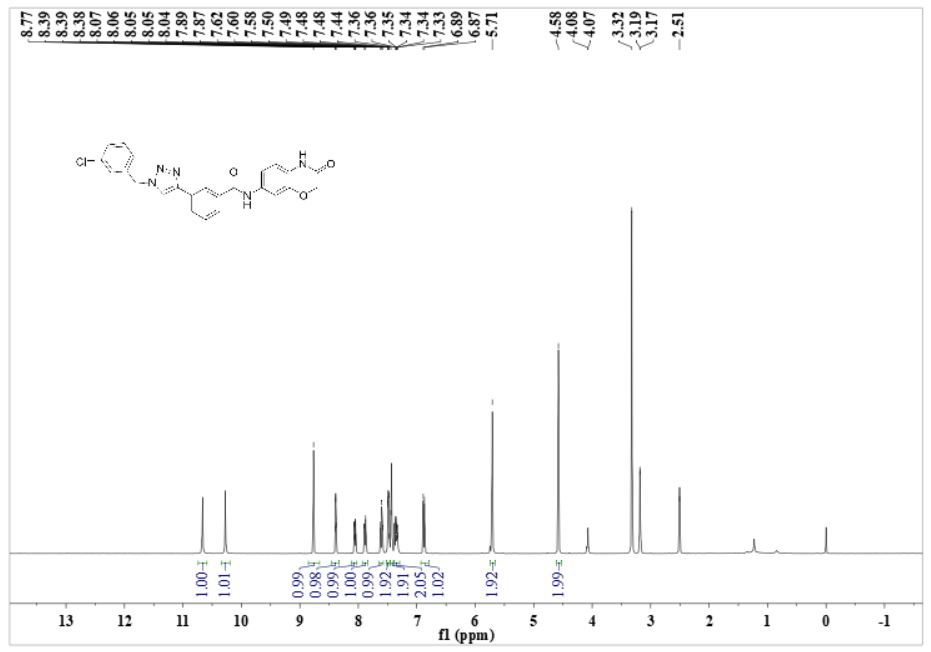


c3


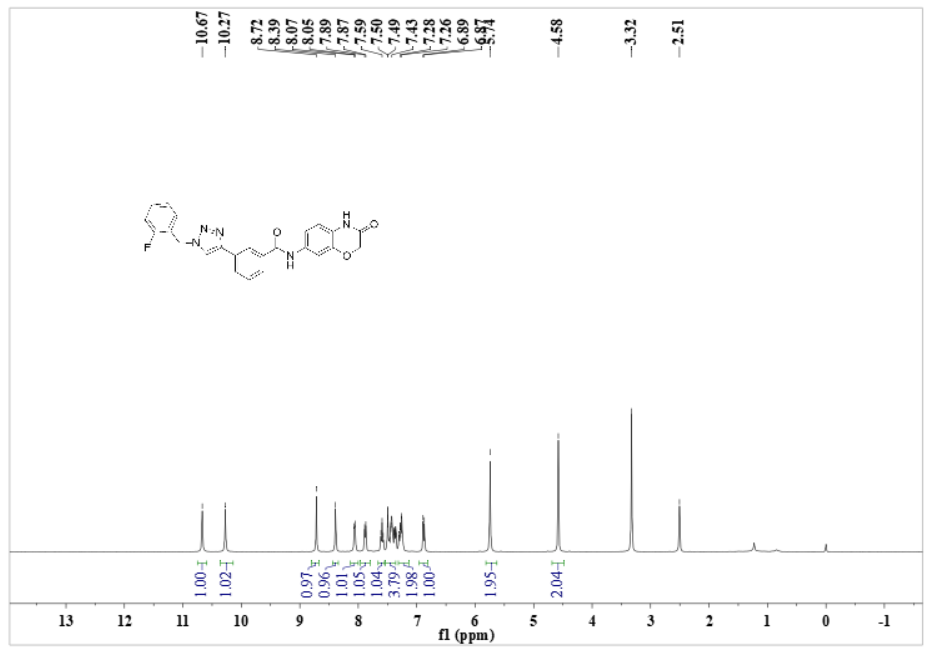


c4


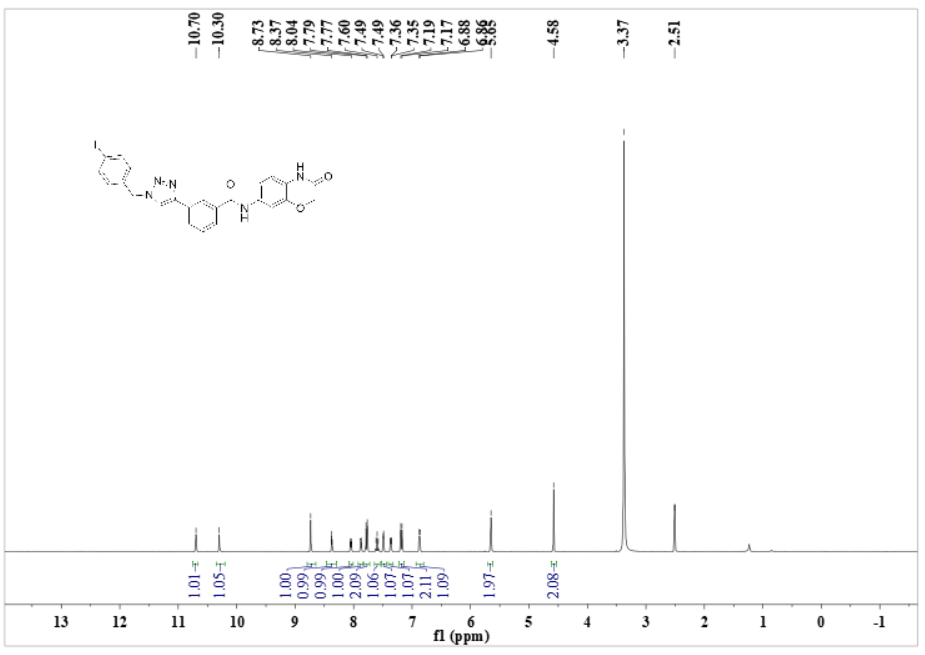


c5


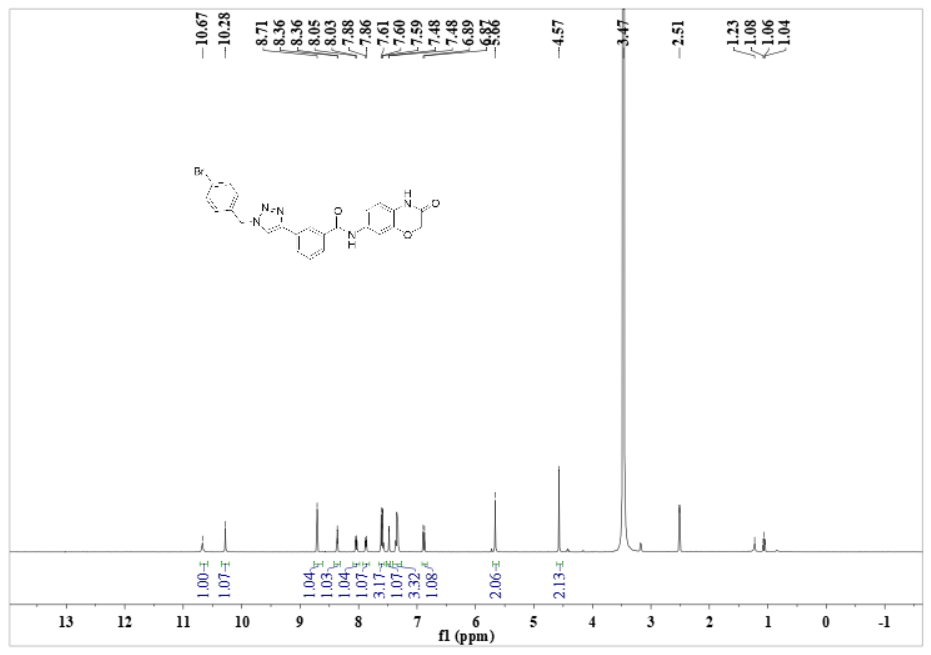


c6


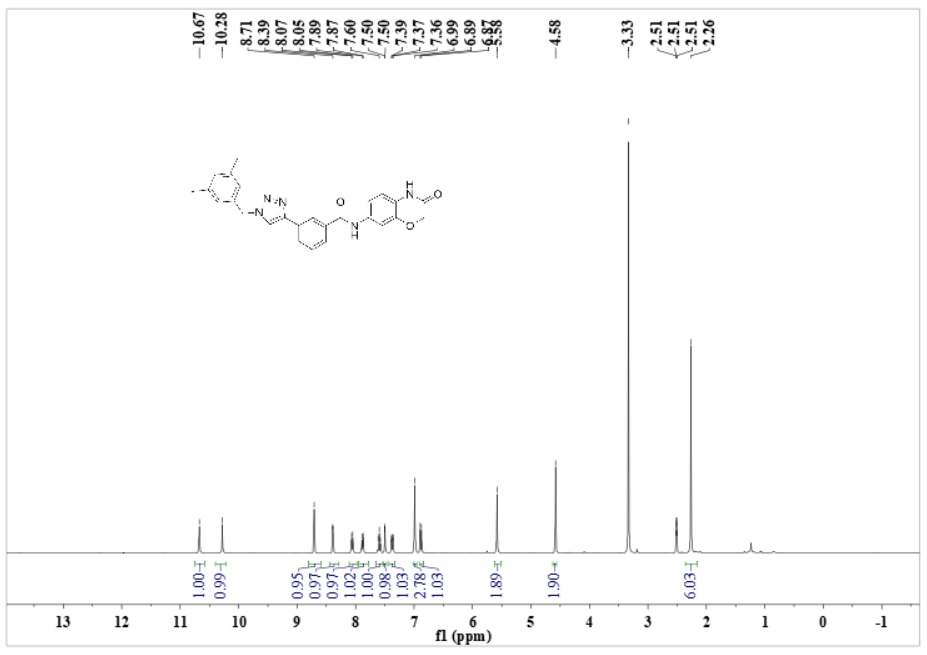


c7


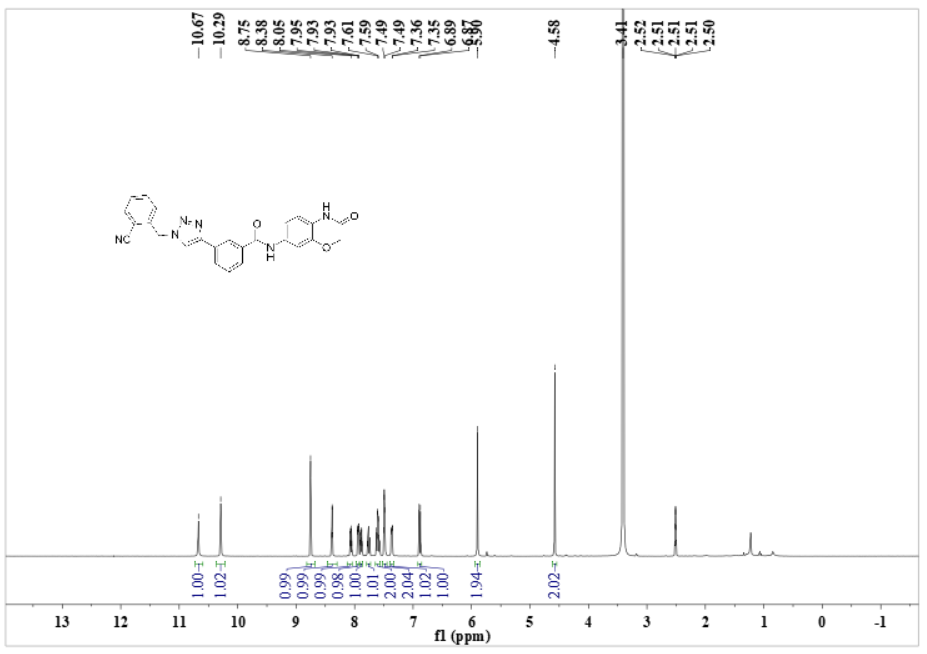


c8


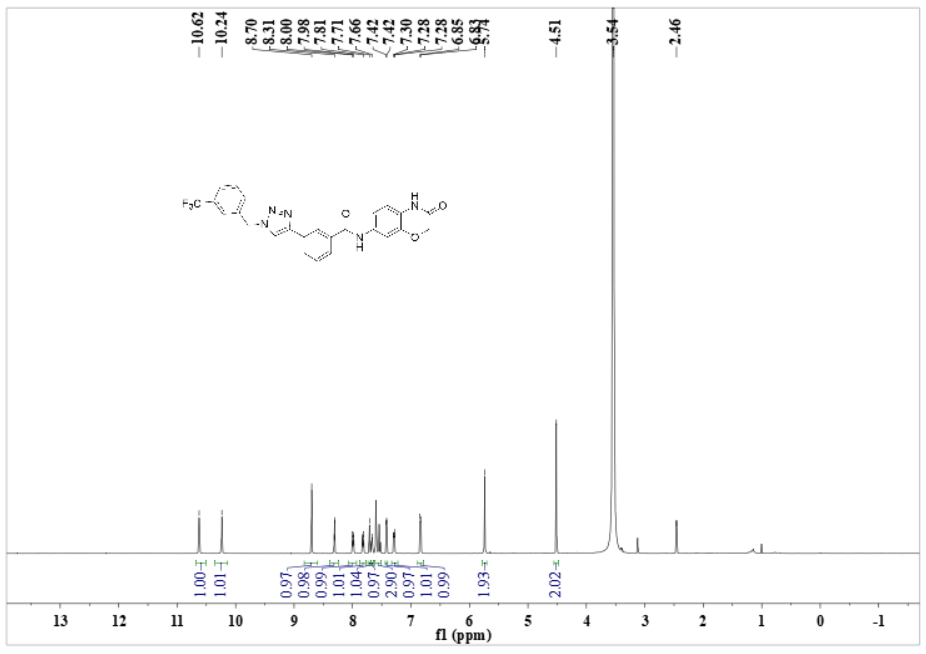


c9


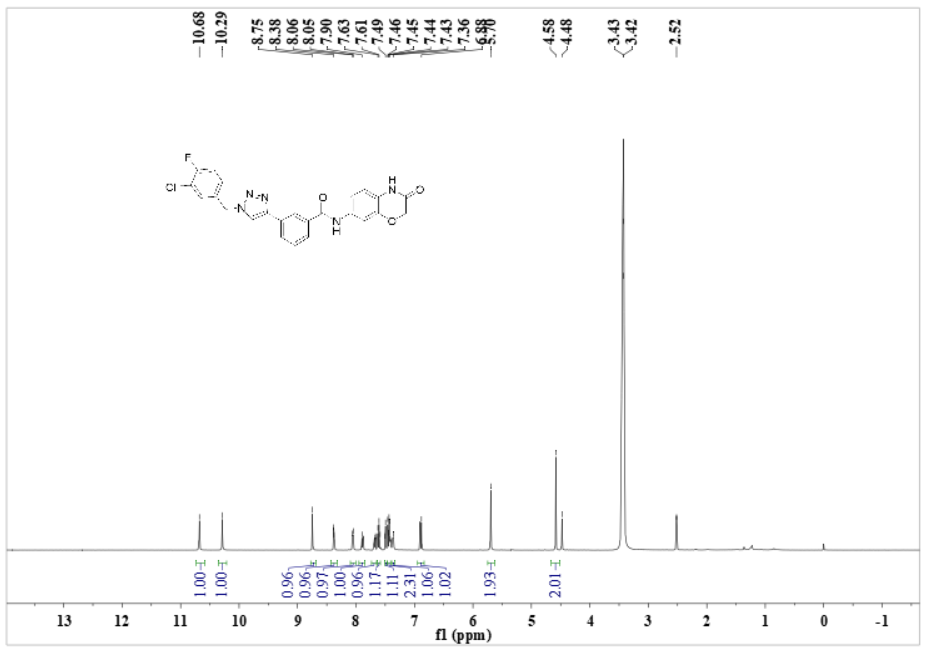


c10


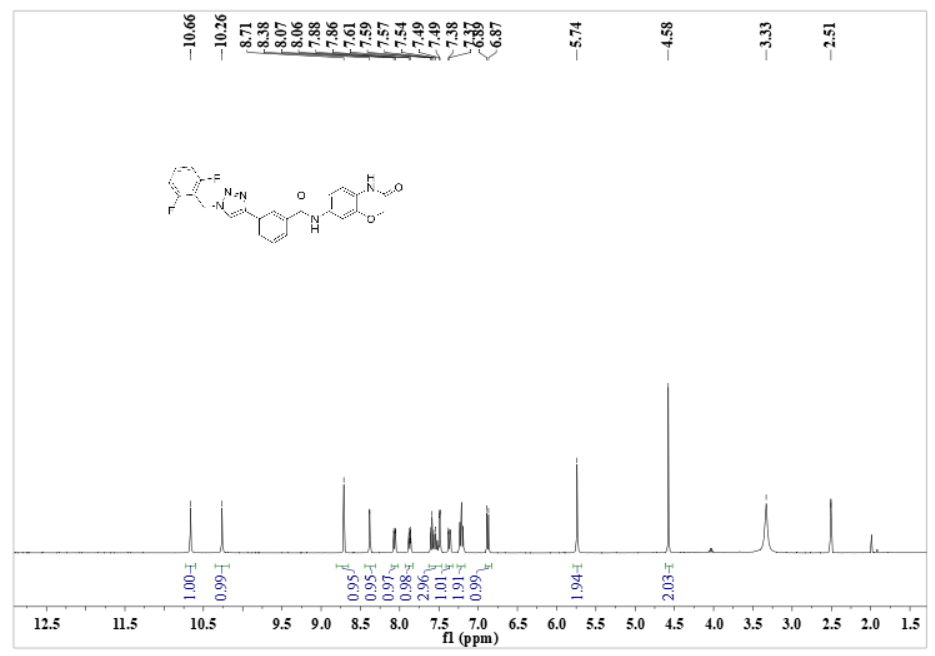


c11


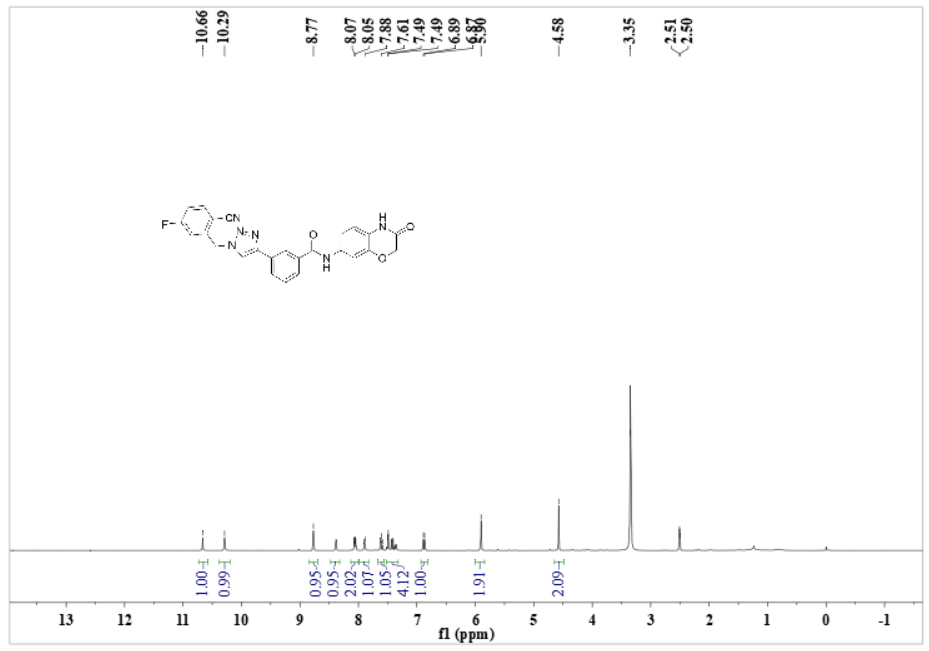


c12


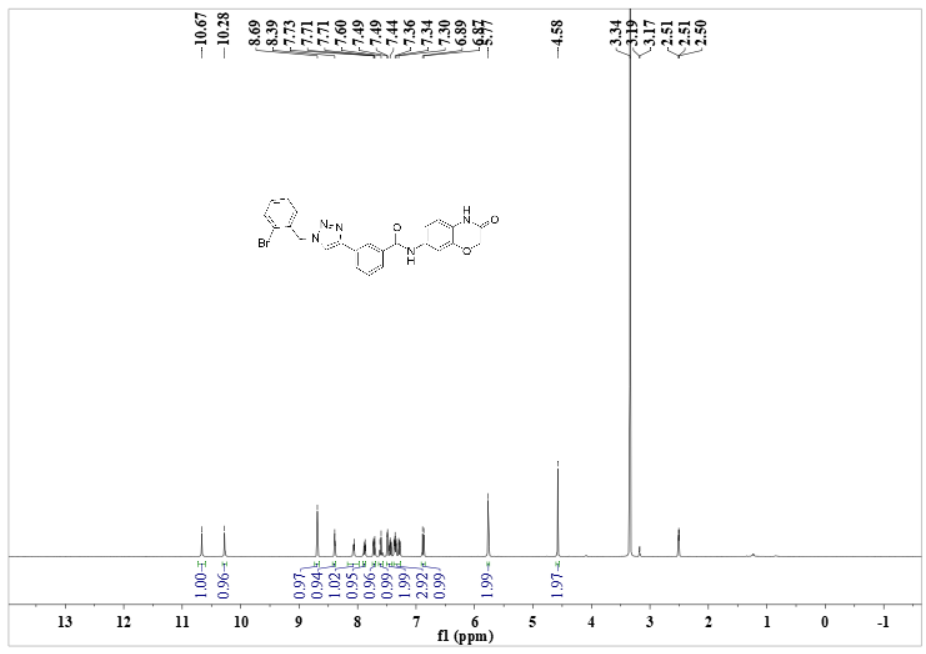


c13


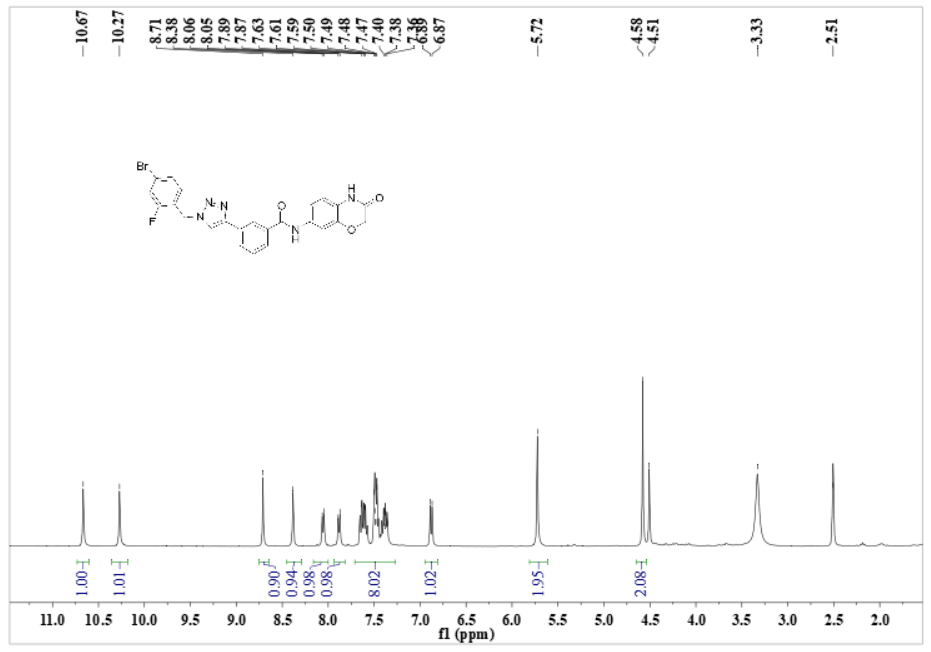


c14


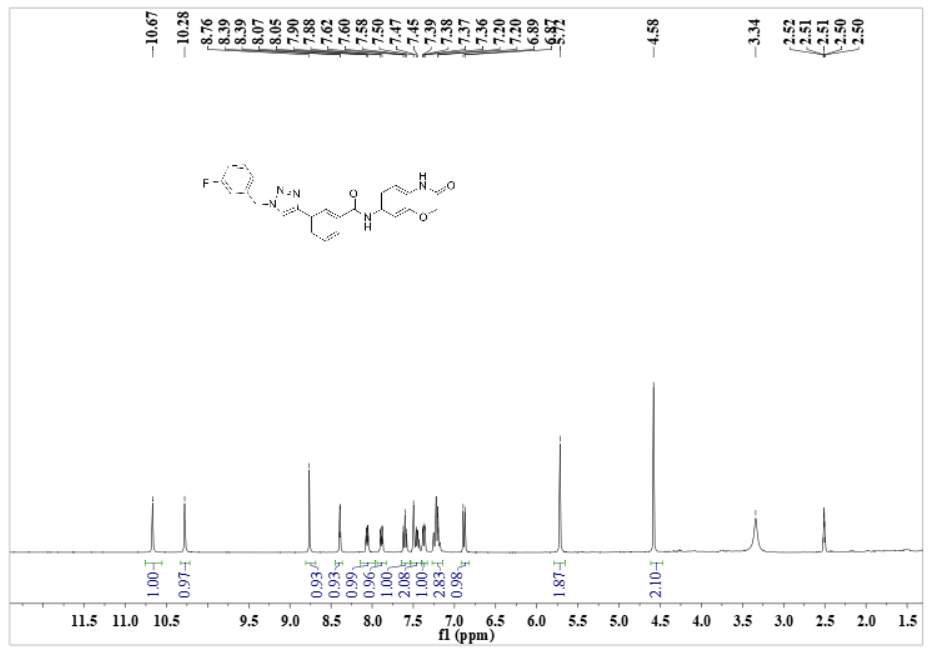


c15


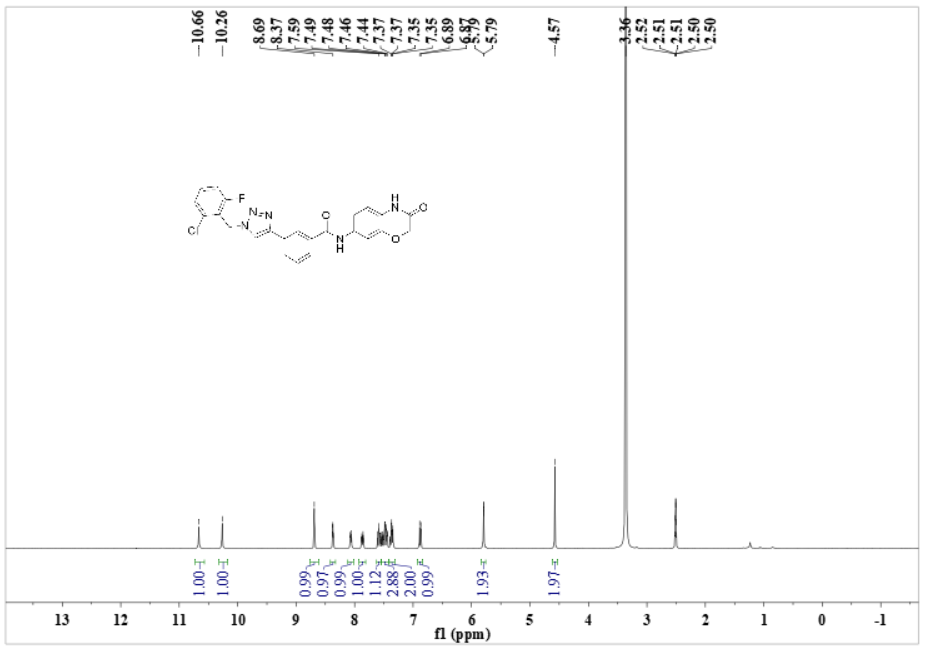


c16


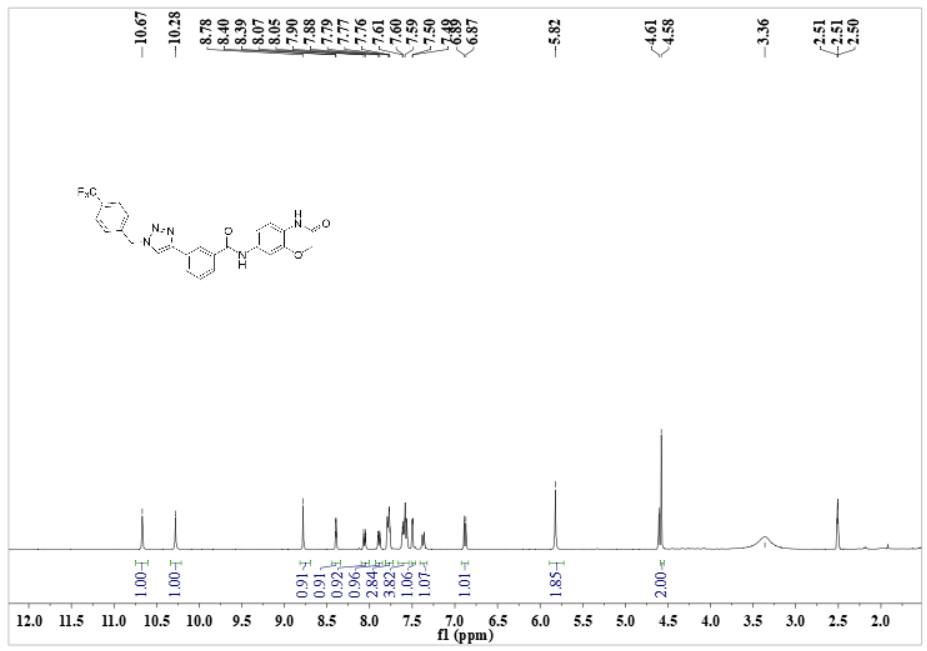


c17


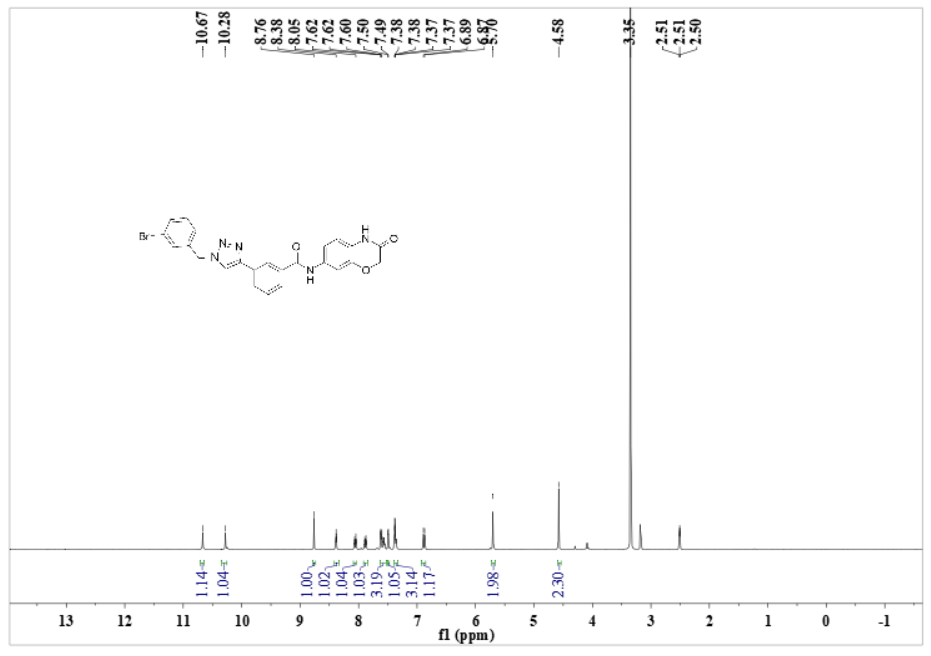


c18


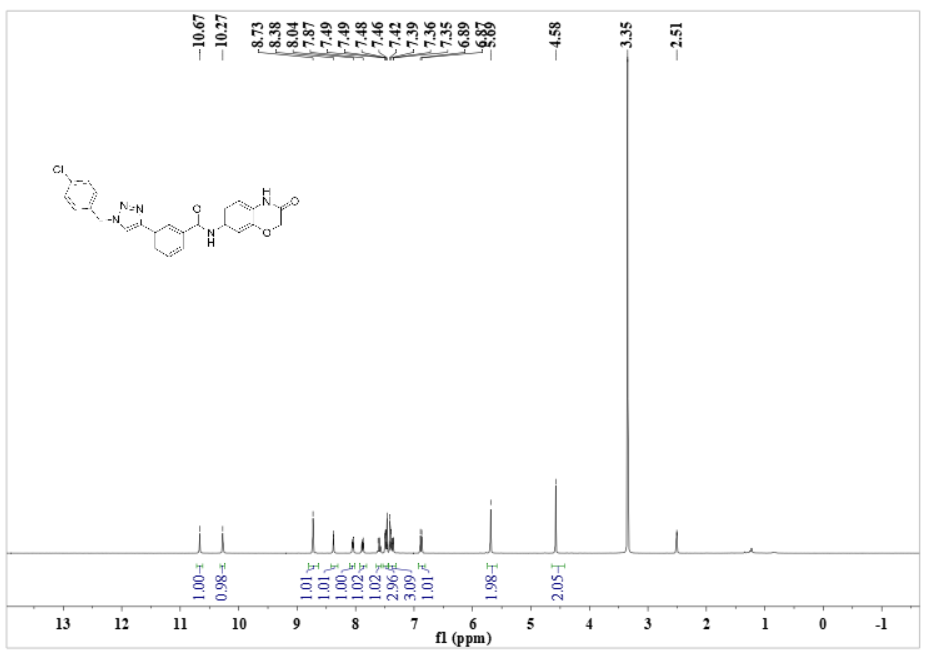


c19


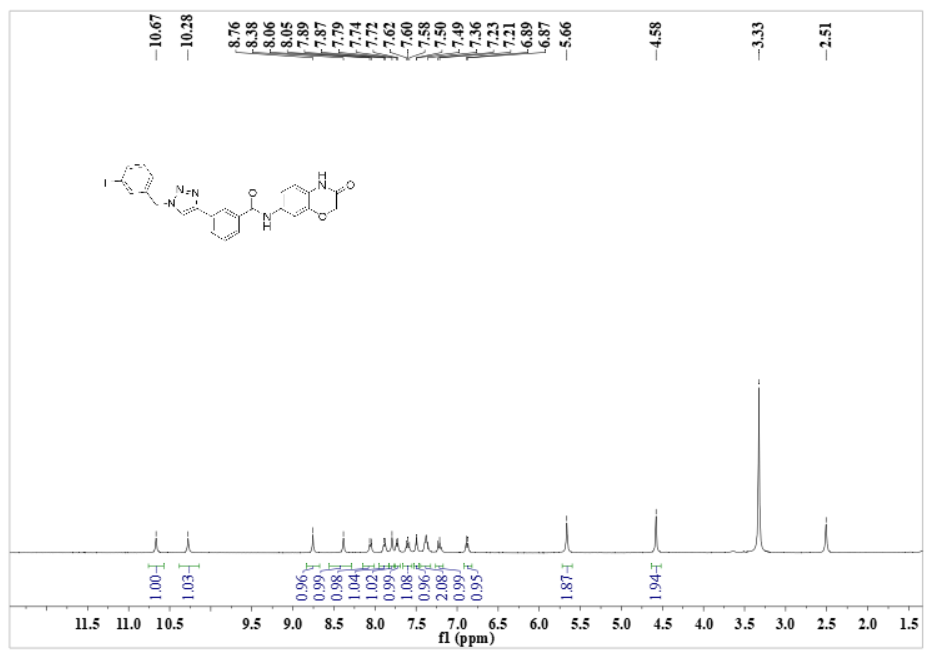


c20


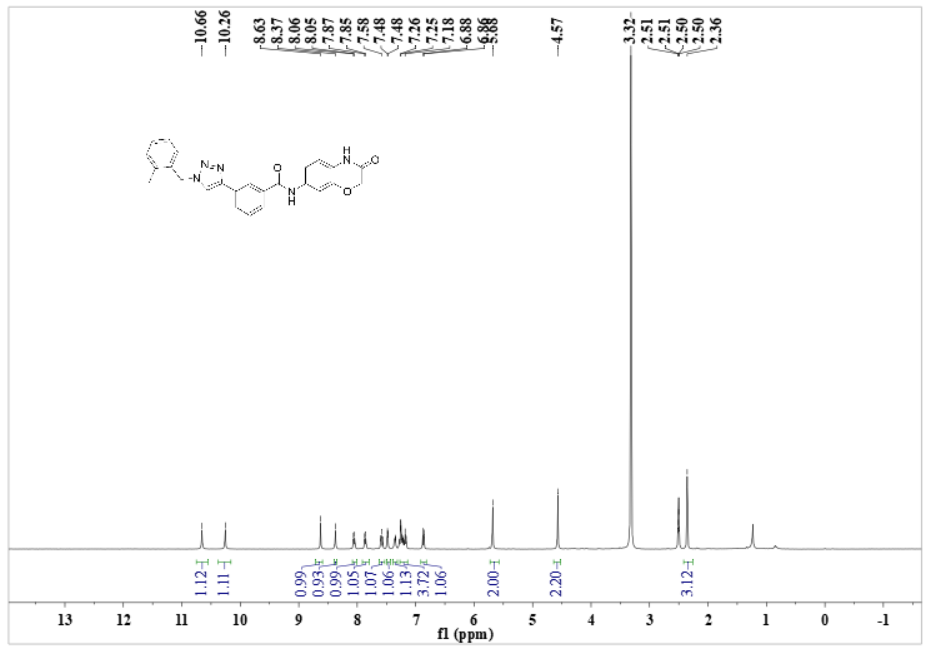

Supplement: Supplementary file 1 [file Supplementaryfile1.docx]
