# Supplementary material for: 2H-1,4-Benzoxazin-3(4H)-one linked 1,2,3-triazole derivatives and their study on inducing DNA damage in tumor cells
Source: Front Pharmacol. 2025 Aug 15;16:1564090. doi: 10.3389/fphar.2025.1564090 (PMC12394750; doi:10.3389/fphar.2025.1564090)
Supplement: Supplementary file 2 [file Supplementaryfile2.pptx]

## Slide 1
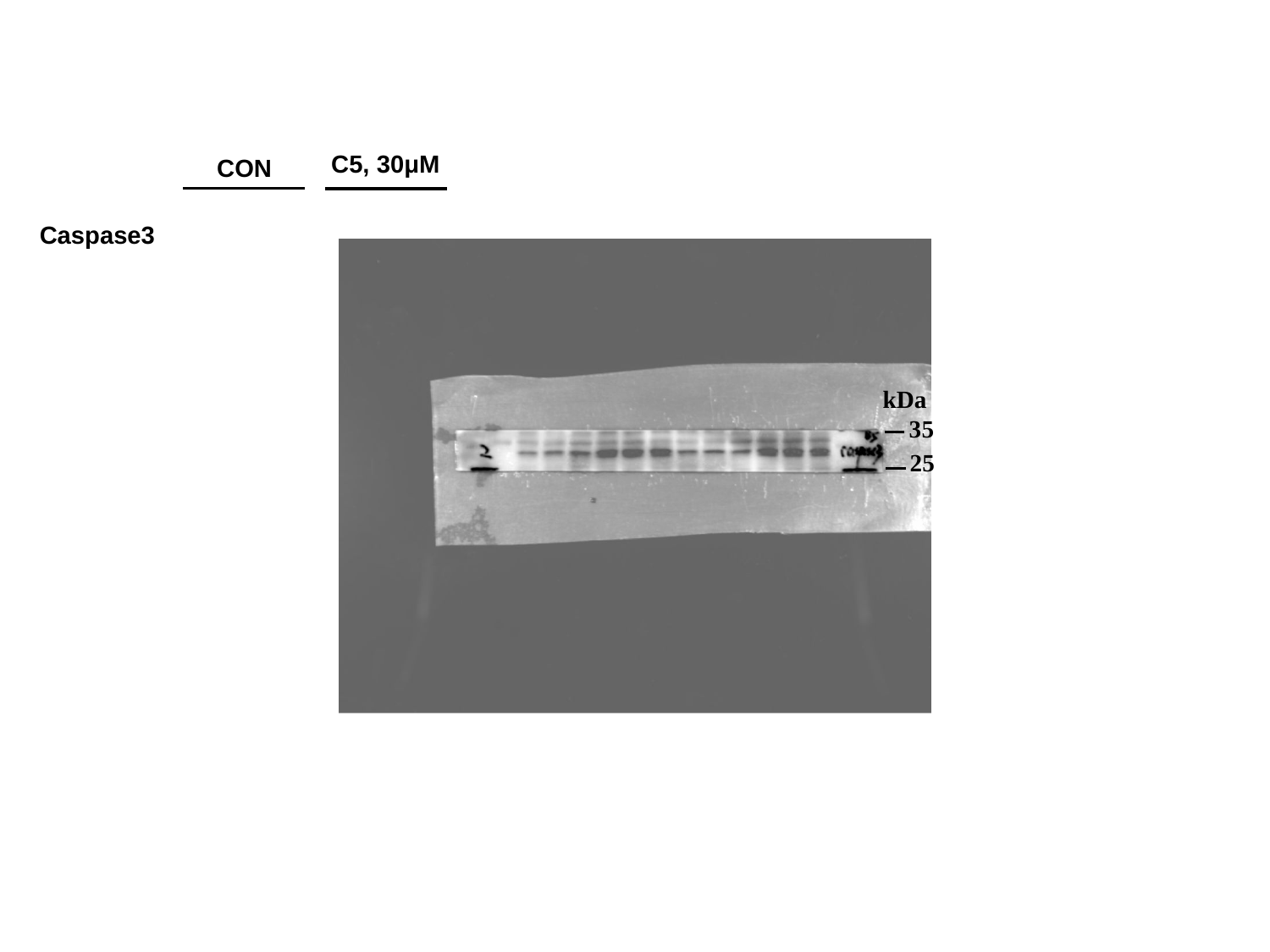

C5, 30μM
CON
Caspase3
kDa
35
25

## Slide 2
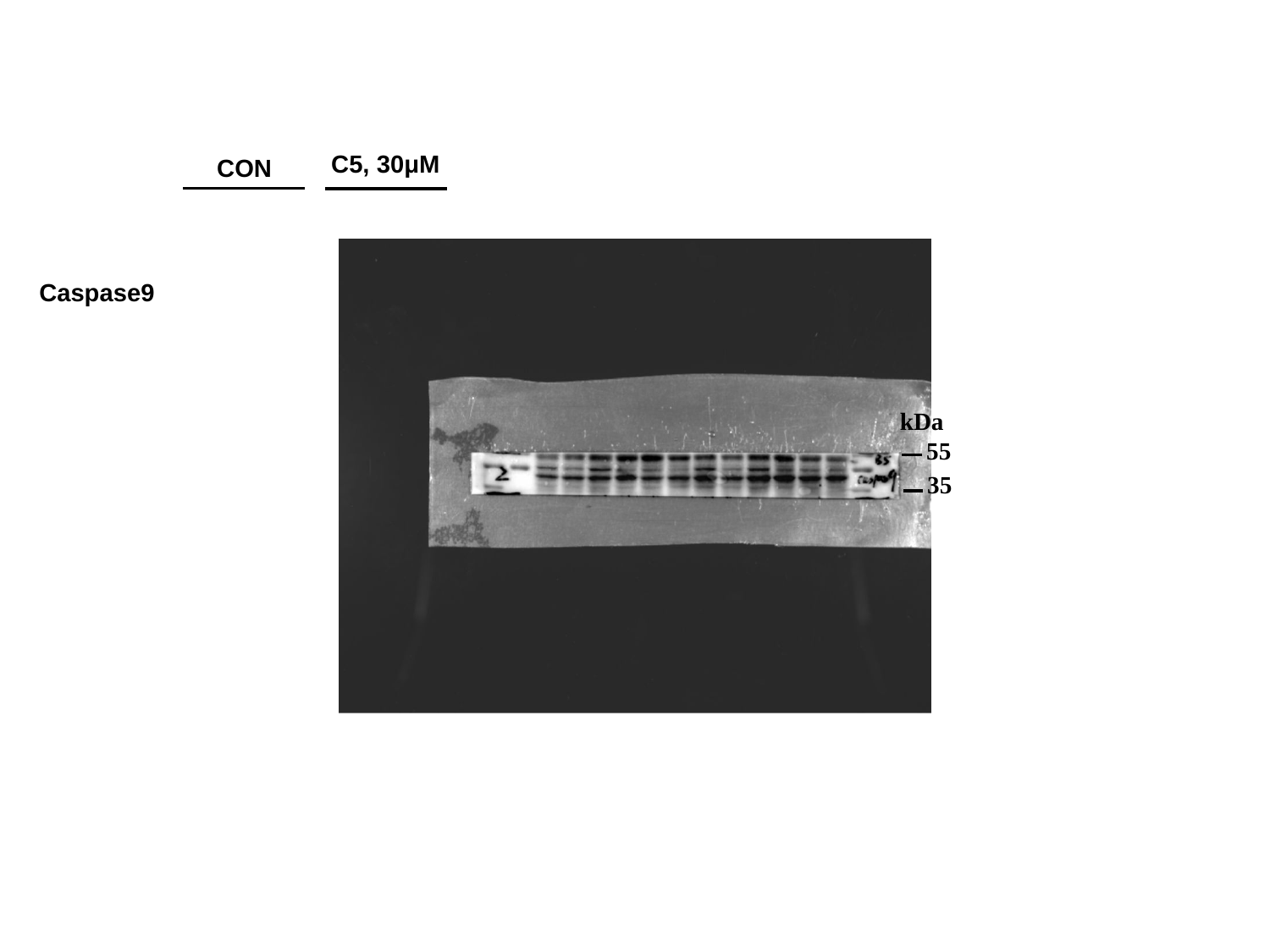

C5, 30μM
CON
Caspase9
kDa
55
35

## Slide 3
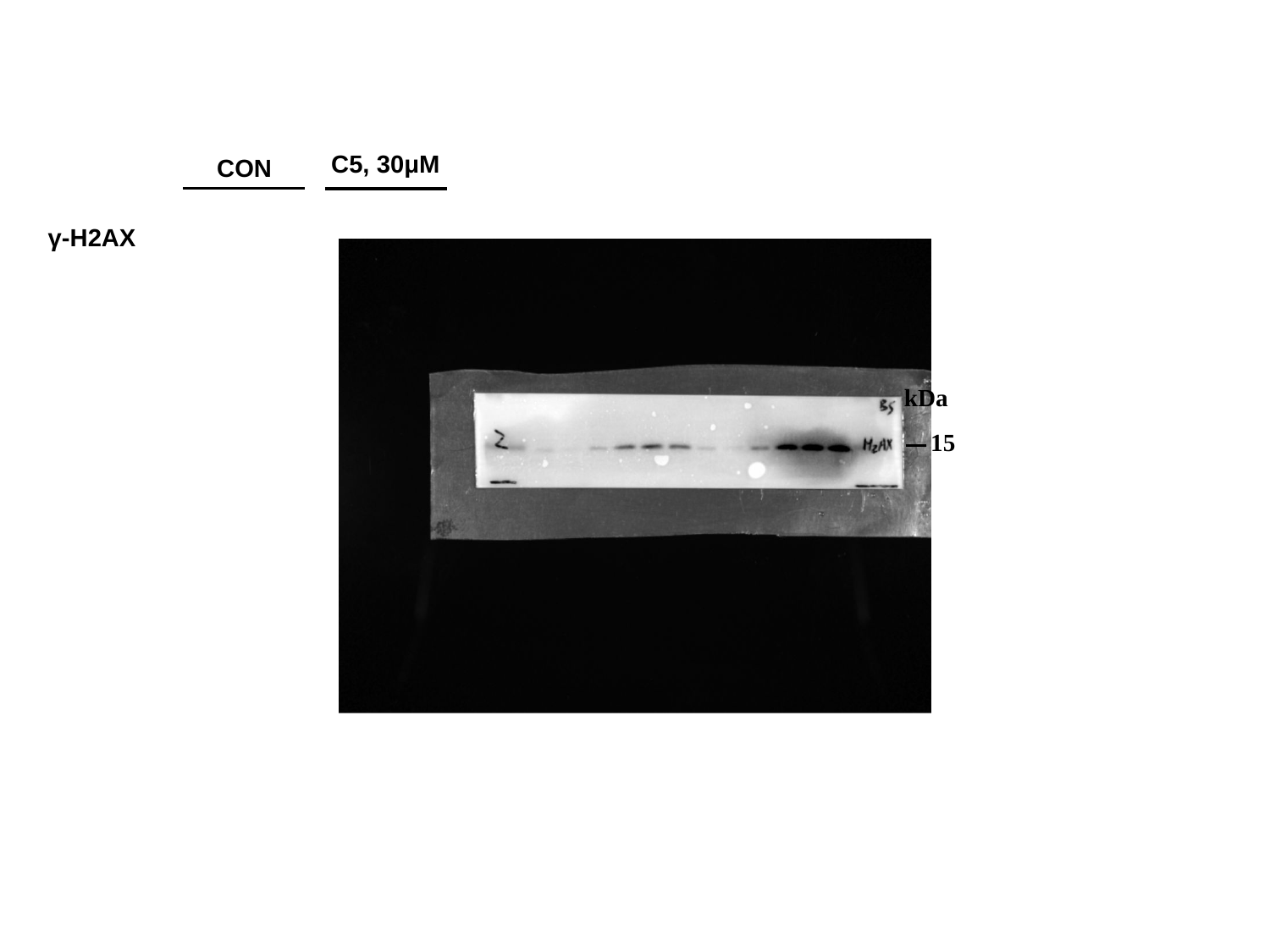

C5, 30μM
CON
γ-H2AX
kDa
15

## Slide 4
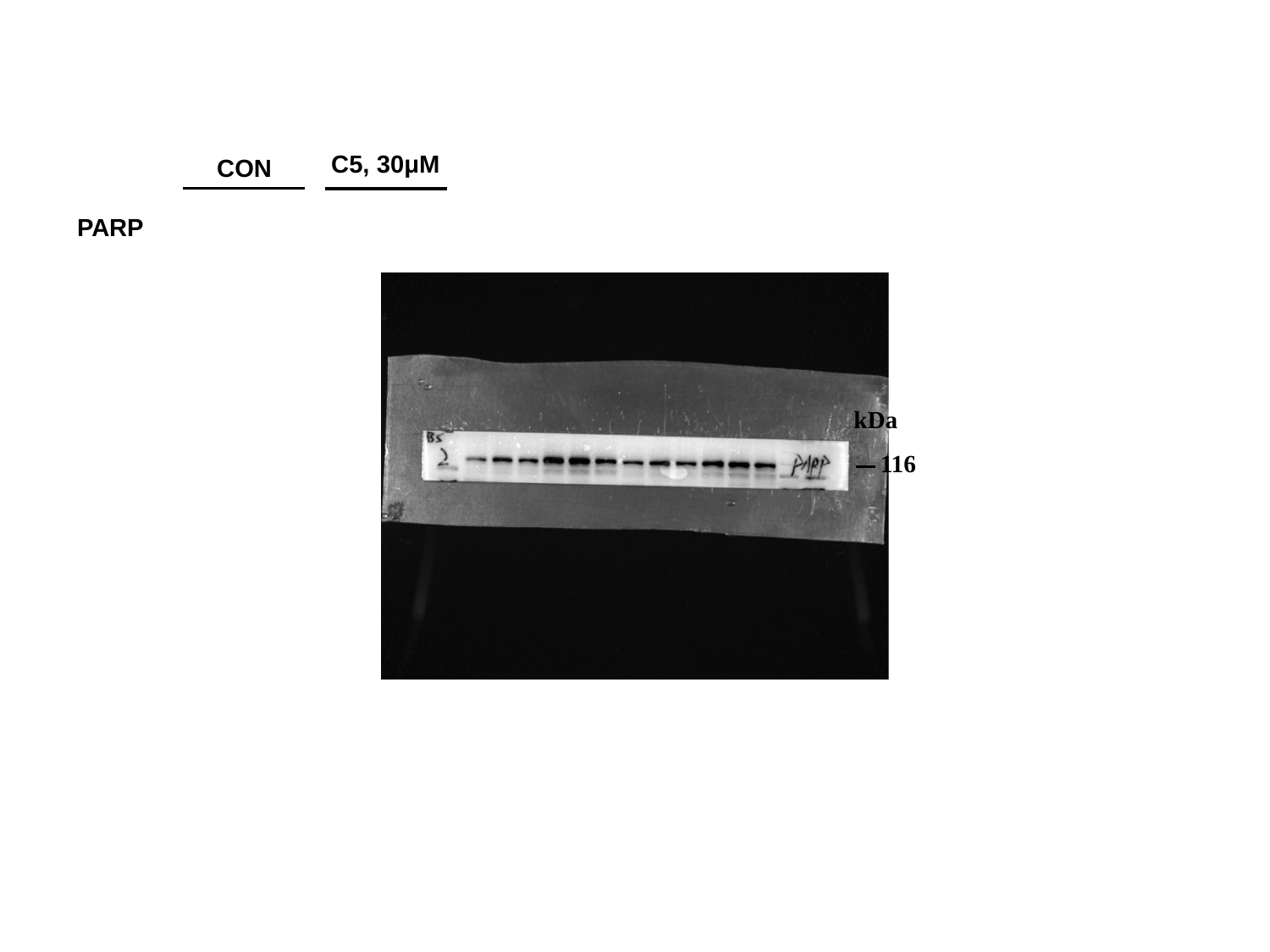

C5, 30μM
CON
PARP
kDa
116

## Slide 5
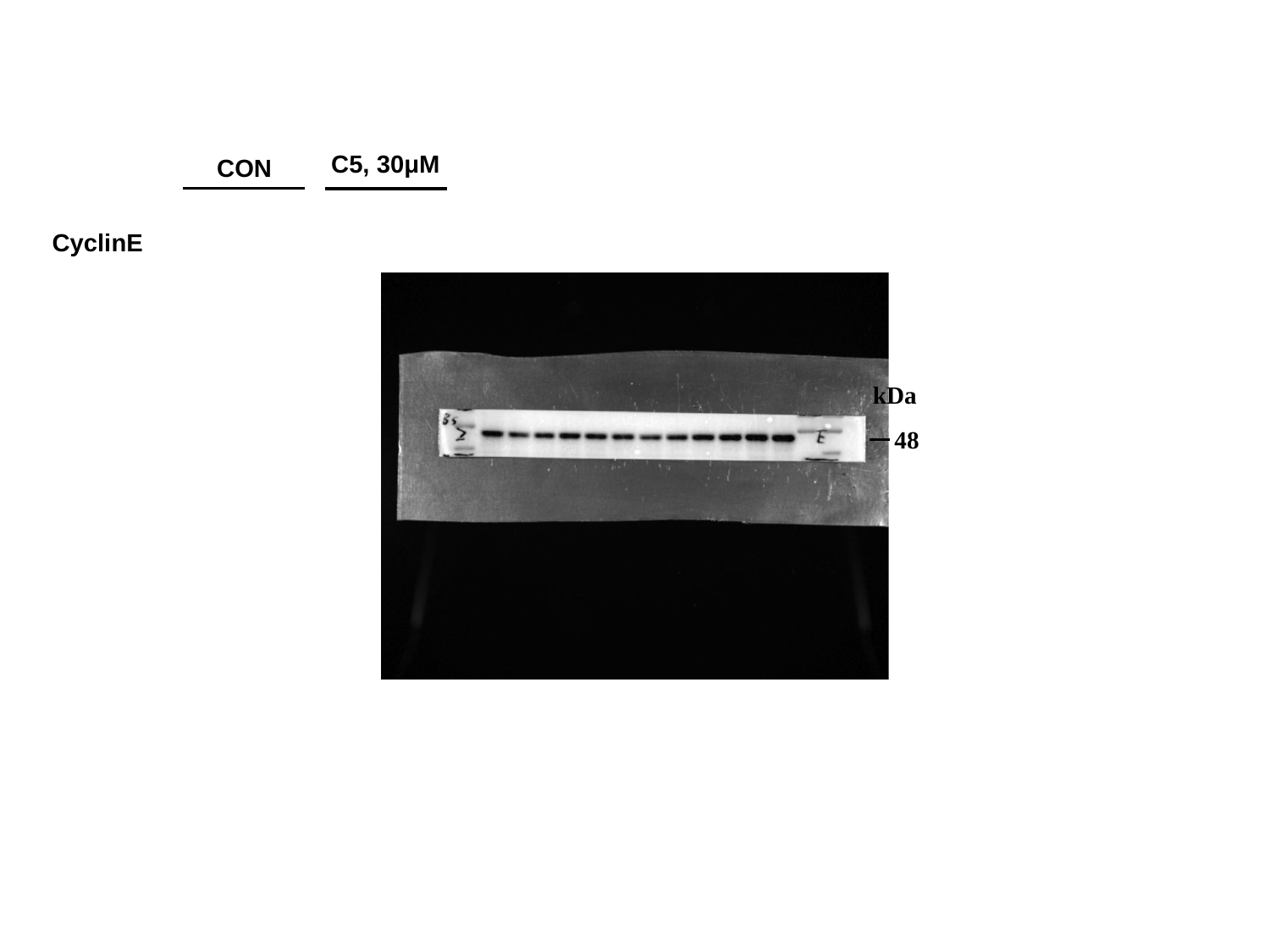

C5, 30μM
CON
CyclinE
kDa
48

## Slide 6
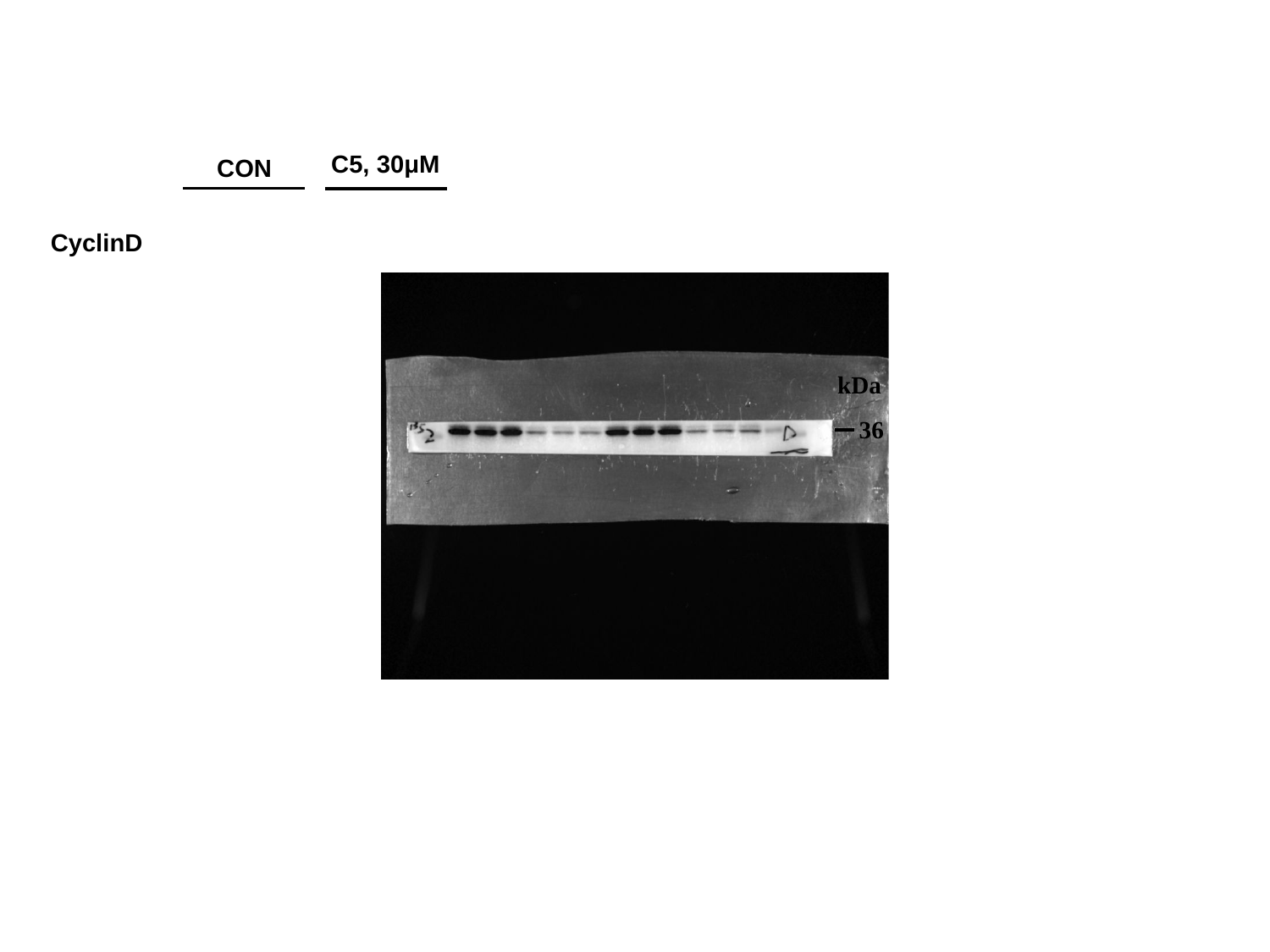

C5, 30μM
CON
CyclinD
kDa
36

## Slide 7
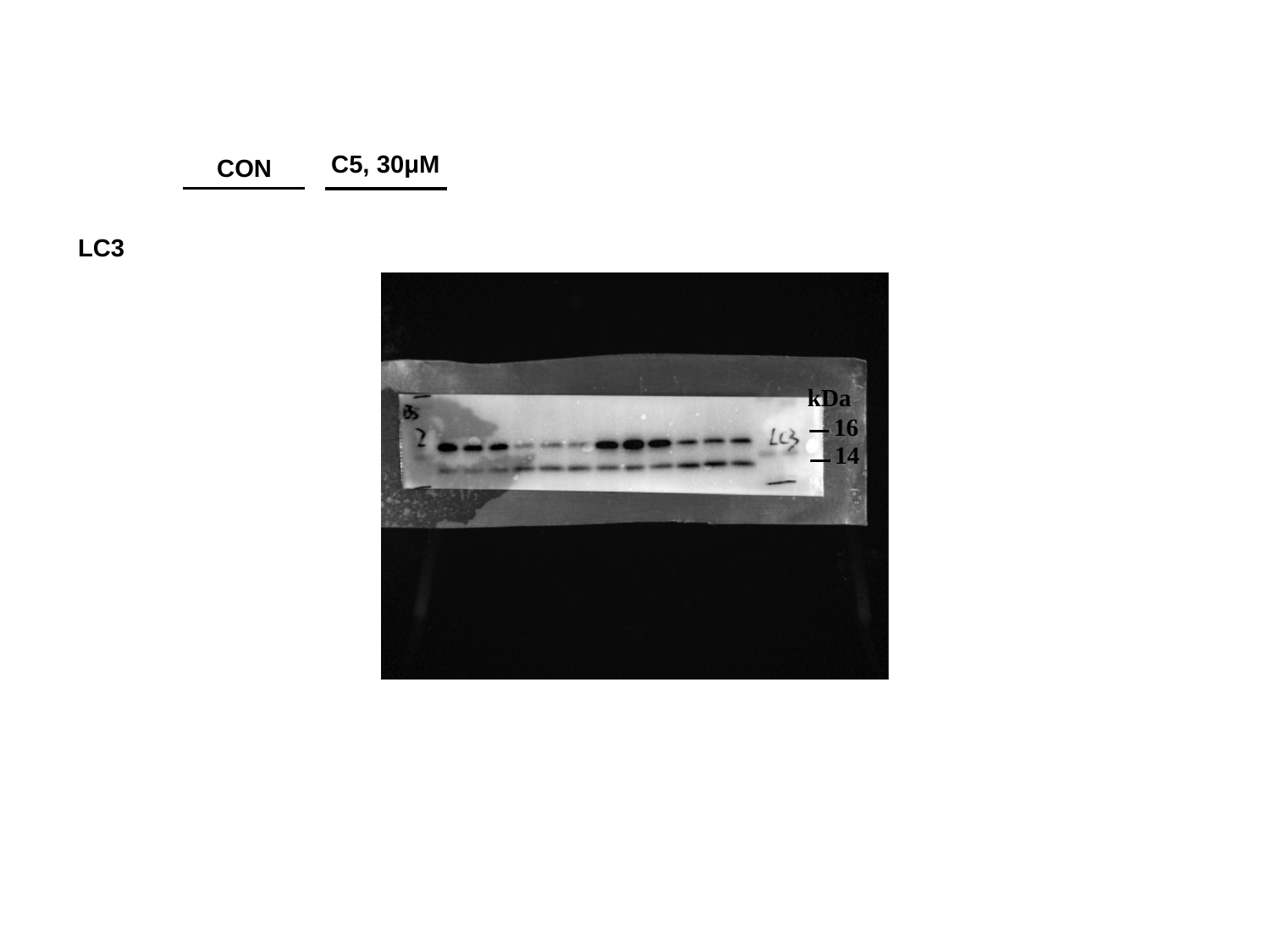

C5, 30μM
CON
LC3
kDa
16
14

## Slide 8
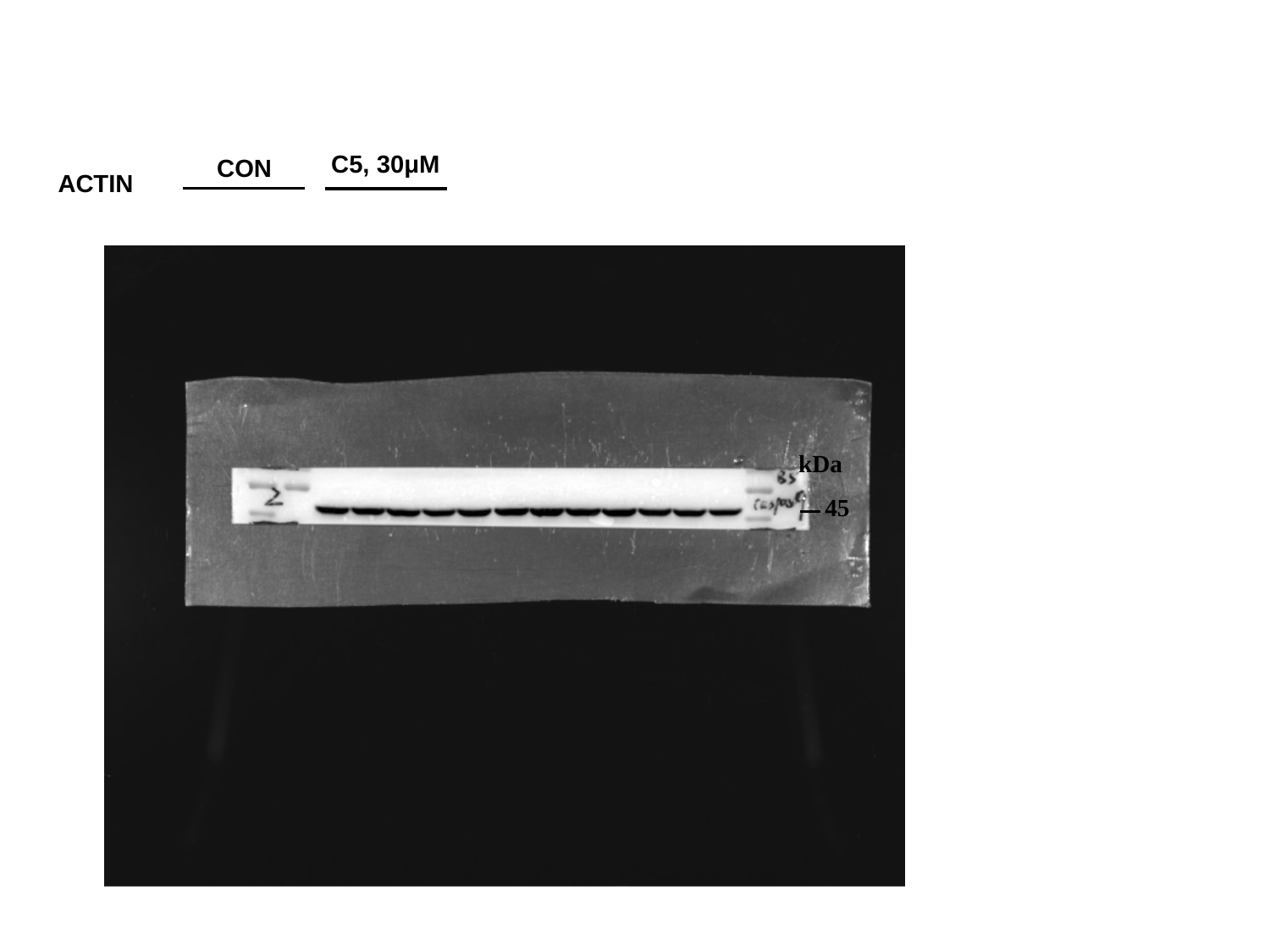

C5, 30μM
CON
ACTIN
kDa
45

## Slide 9
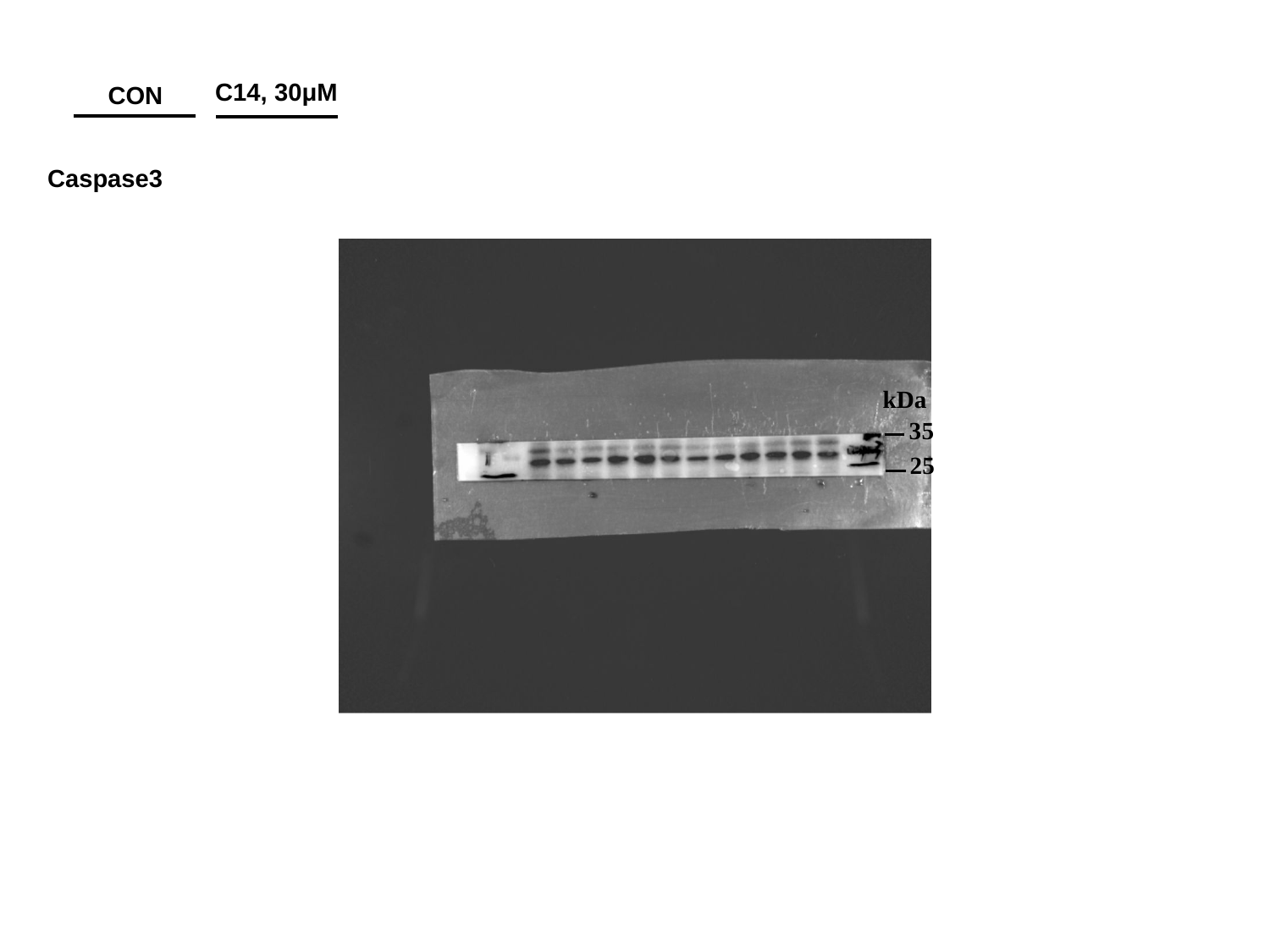

C14, 30μM
CON
Caspase3
kDa
35
25

## Slide 10
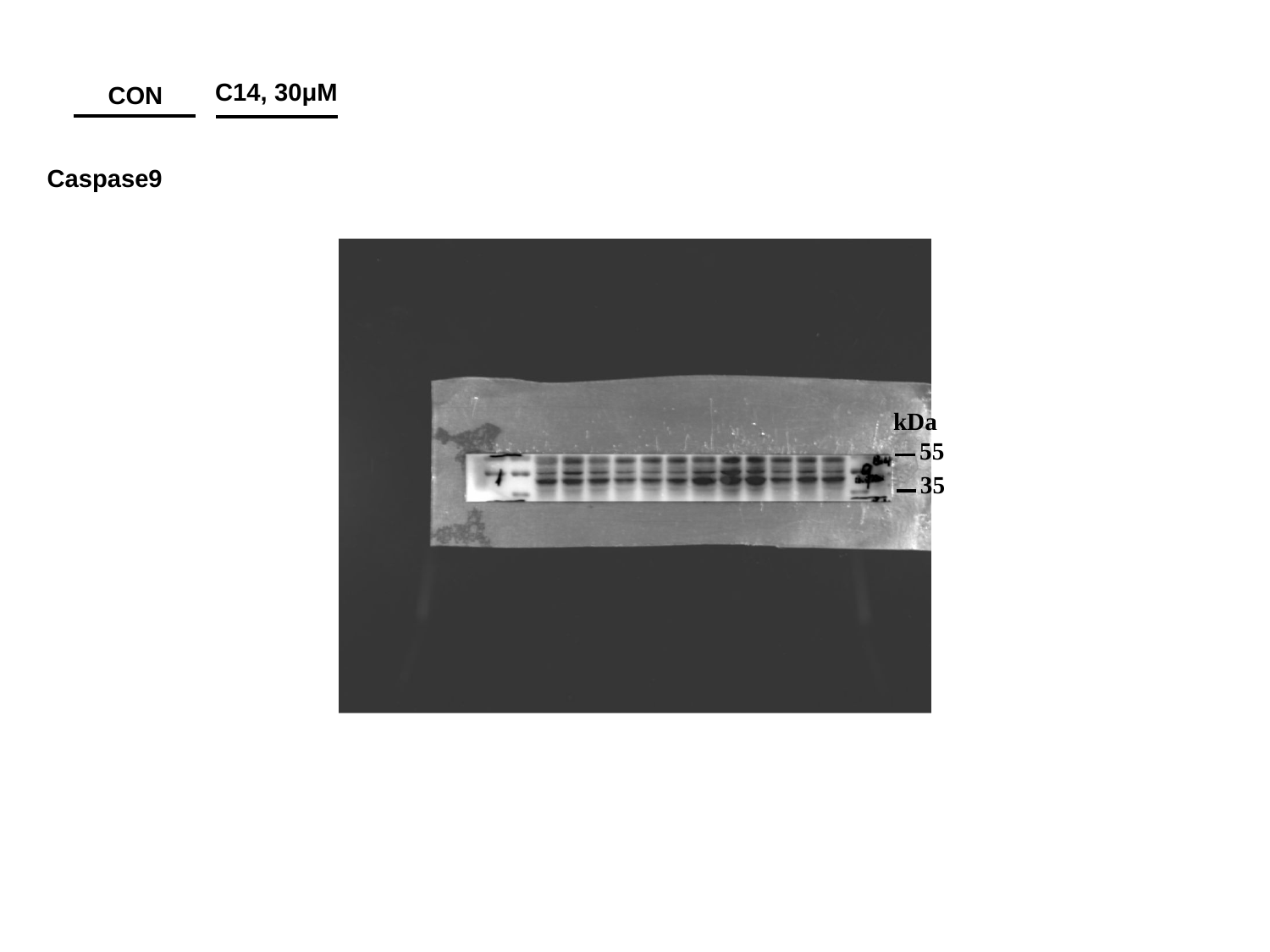

C14, 30μM
CON
Caspase9
kDa
55
35

## Slide 11
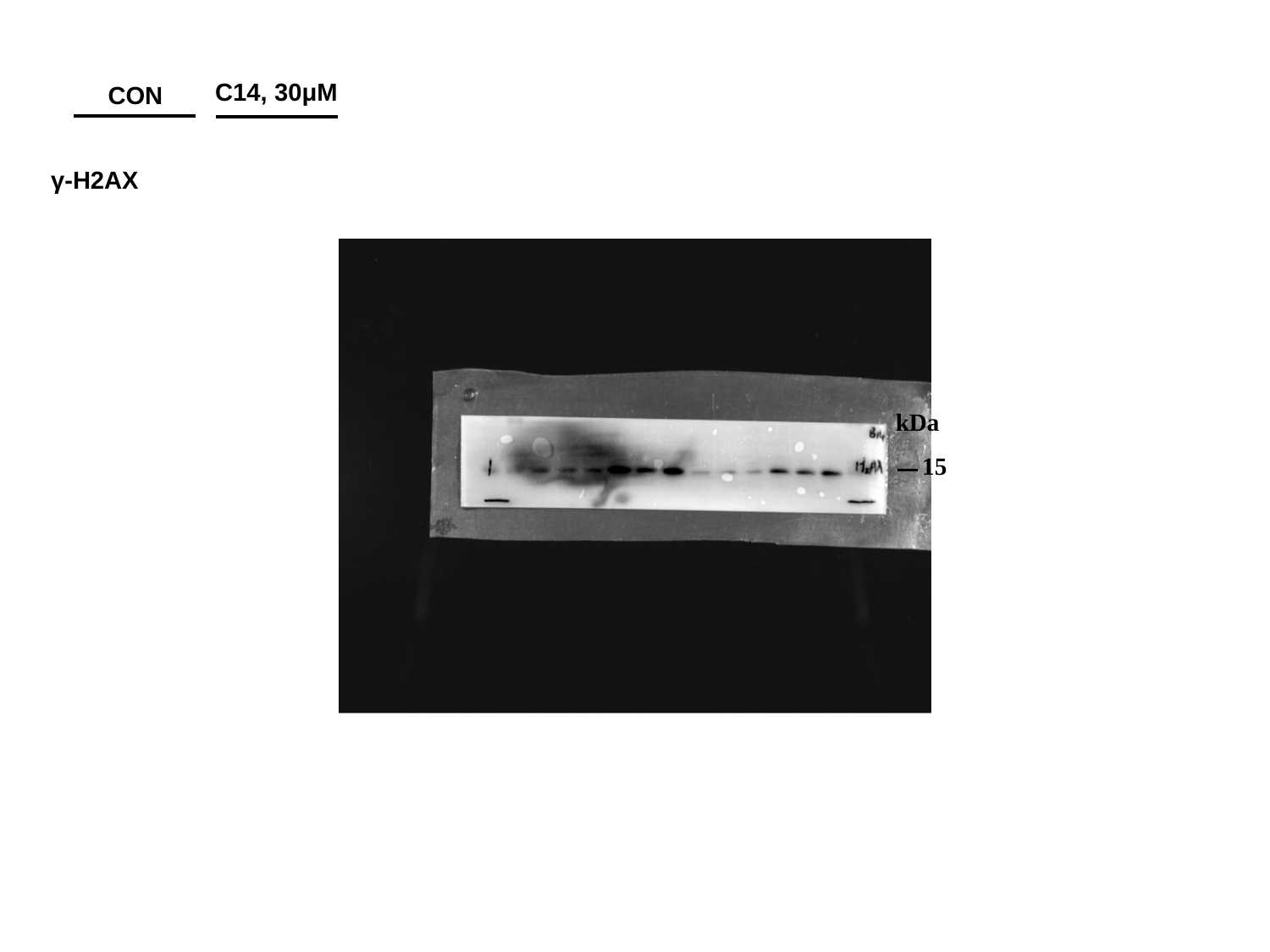

C14, 30μM
CON
γ-H2AX
kDa
15

## Slide 12
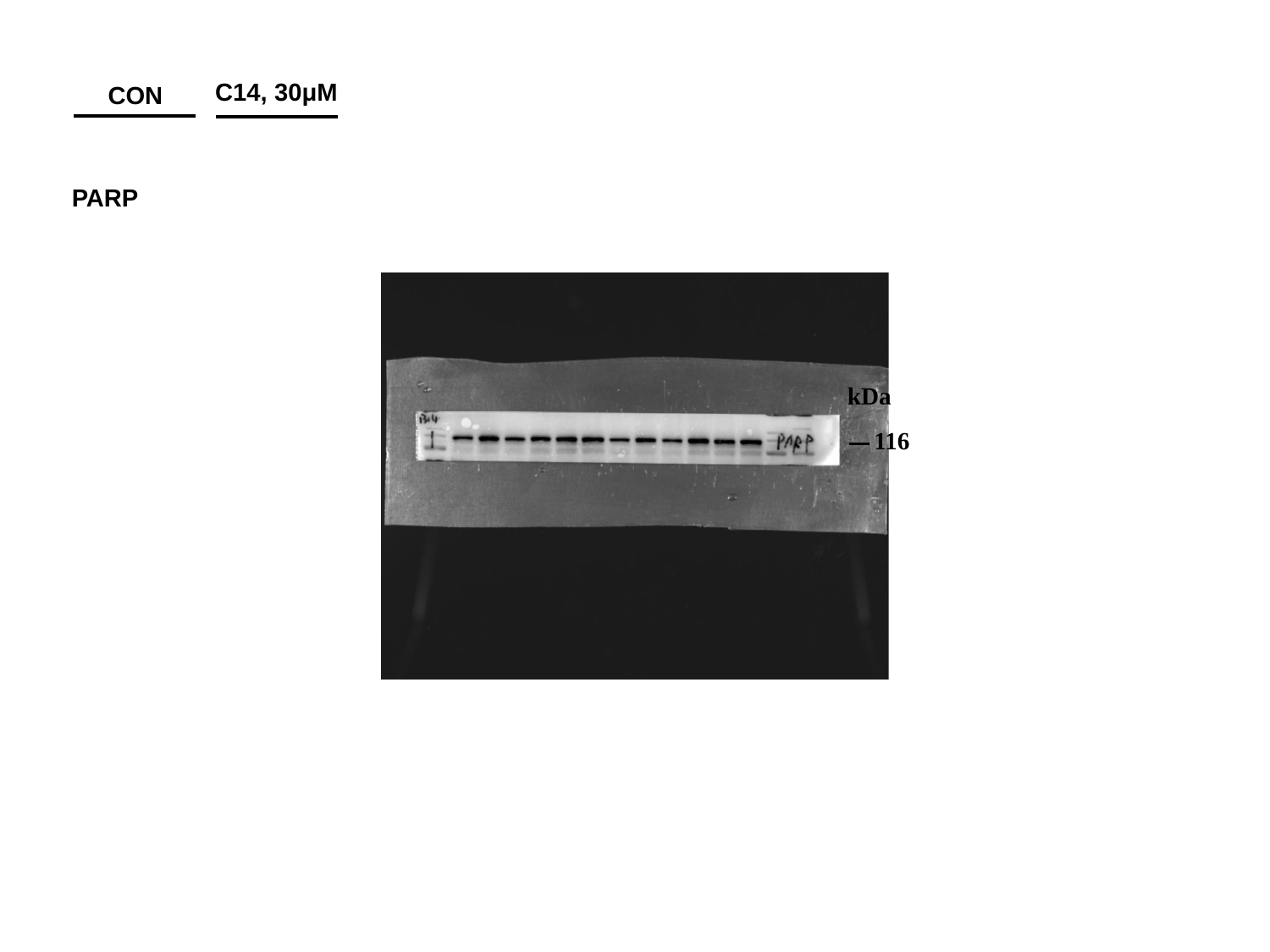

C14, 30μM
CON
PARP
kDa
116

## Slide 13
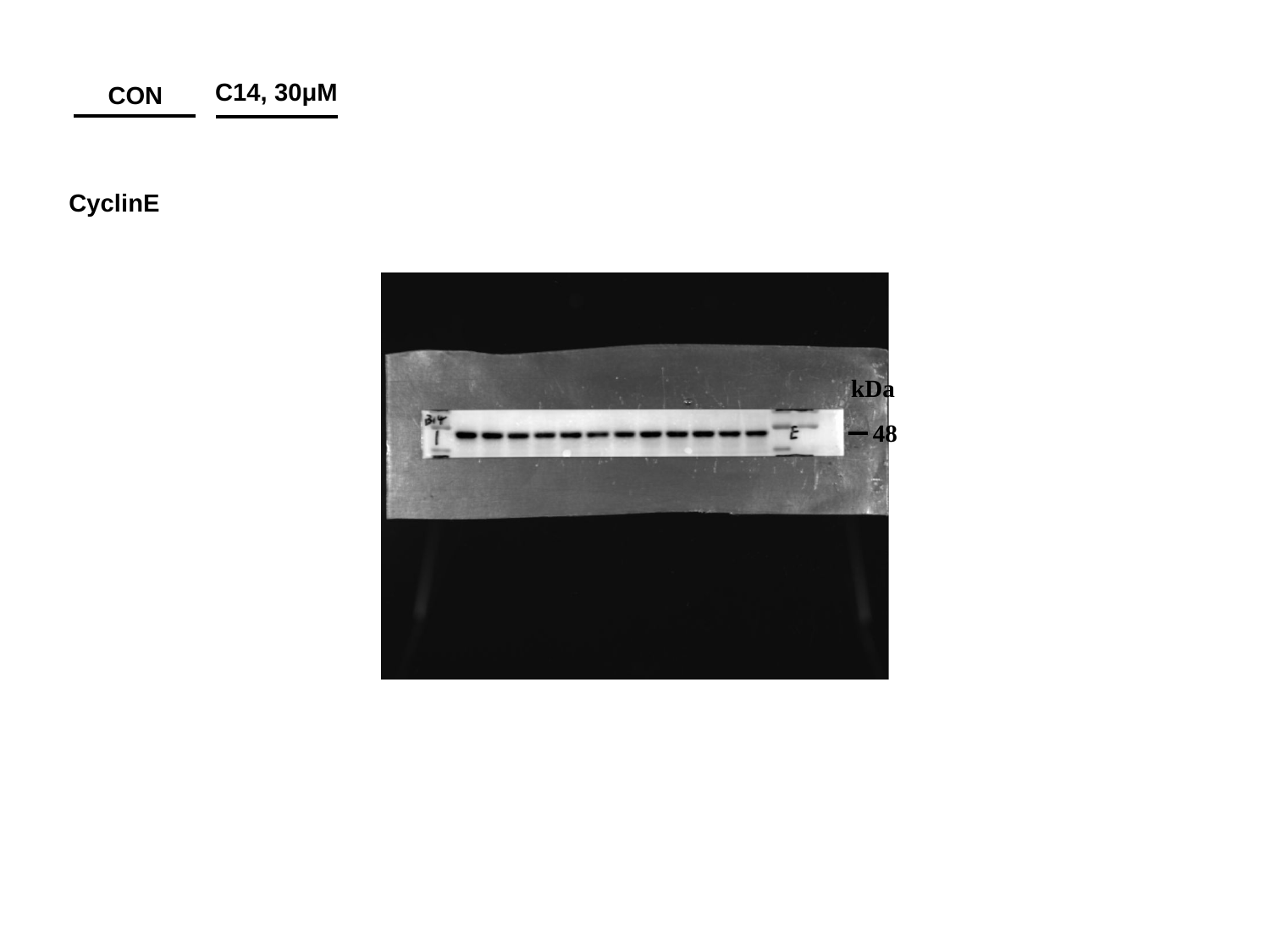

C14, 30μM
CON
CyclinE
kDa
48

## Slide 14
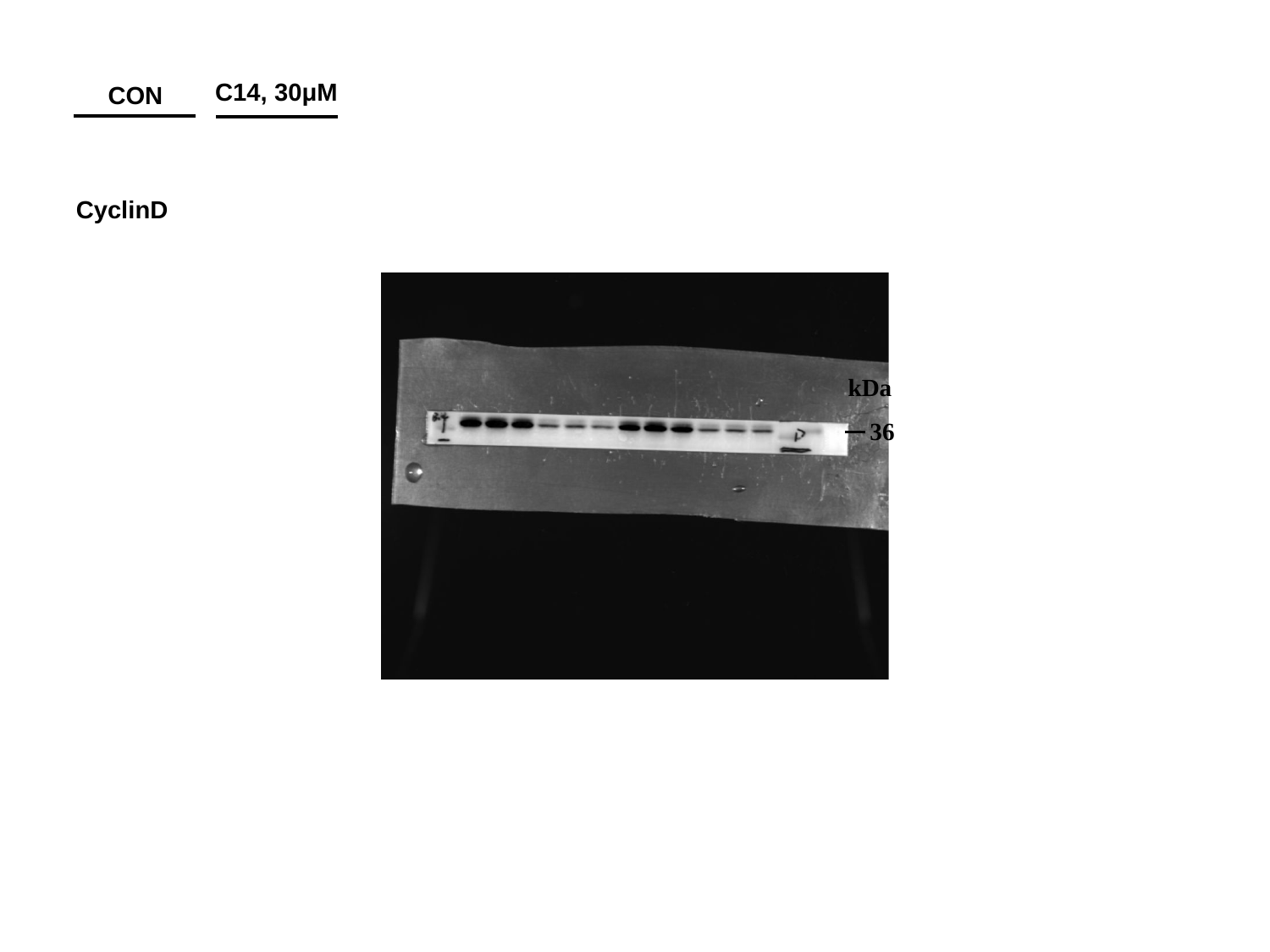

C14, 30μM
CON
CyclinD
kDa
36

## Slide 15
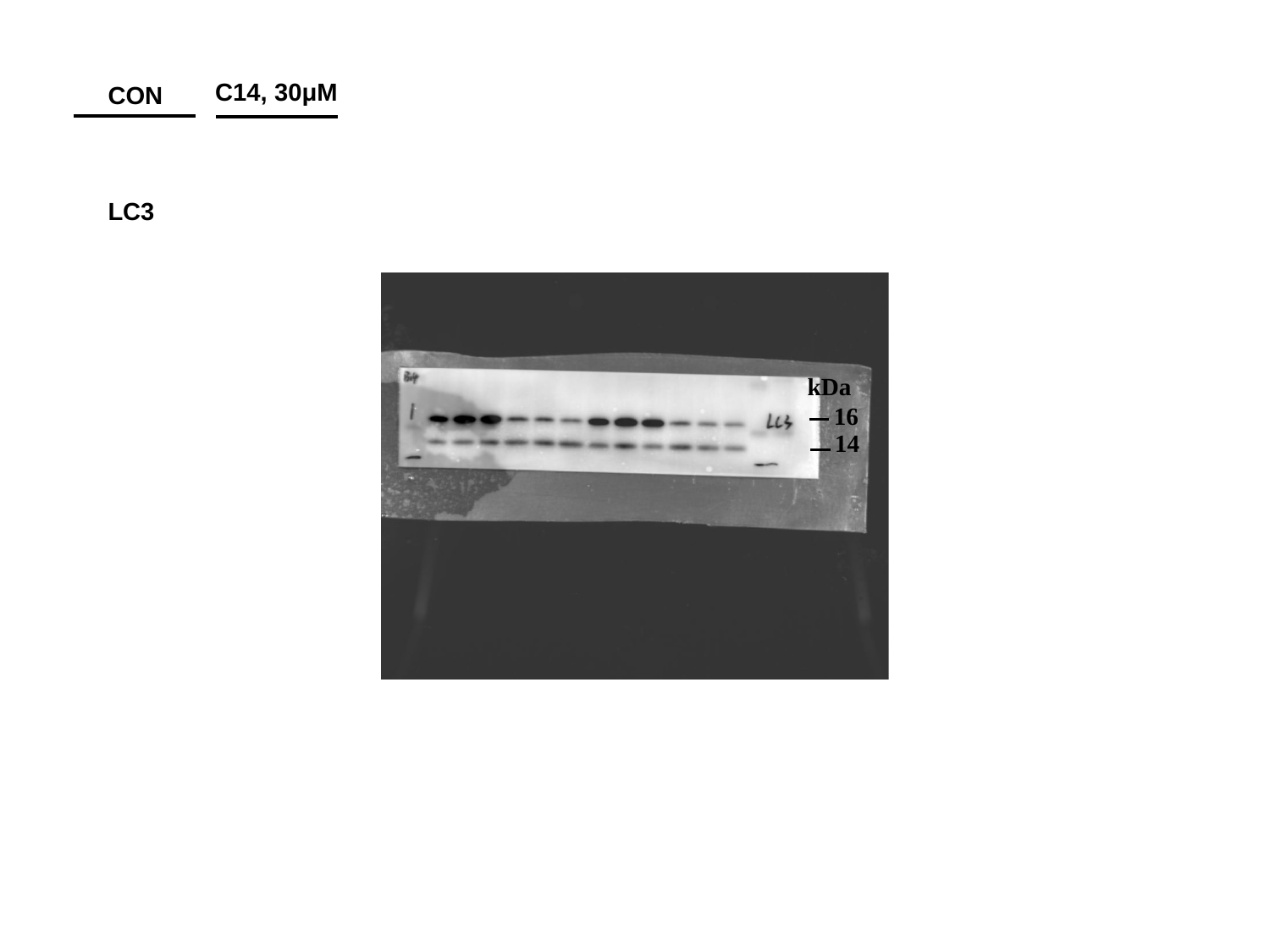

C14, 30μM
CON
LC3
kDa
16
14

## Slide 16
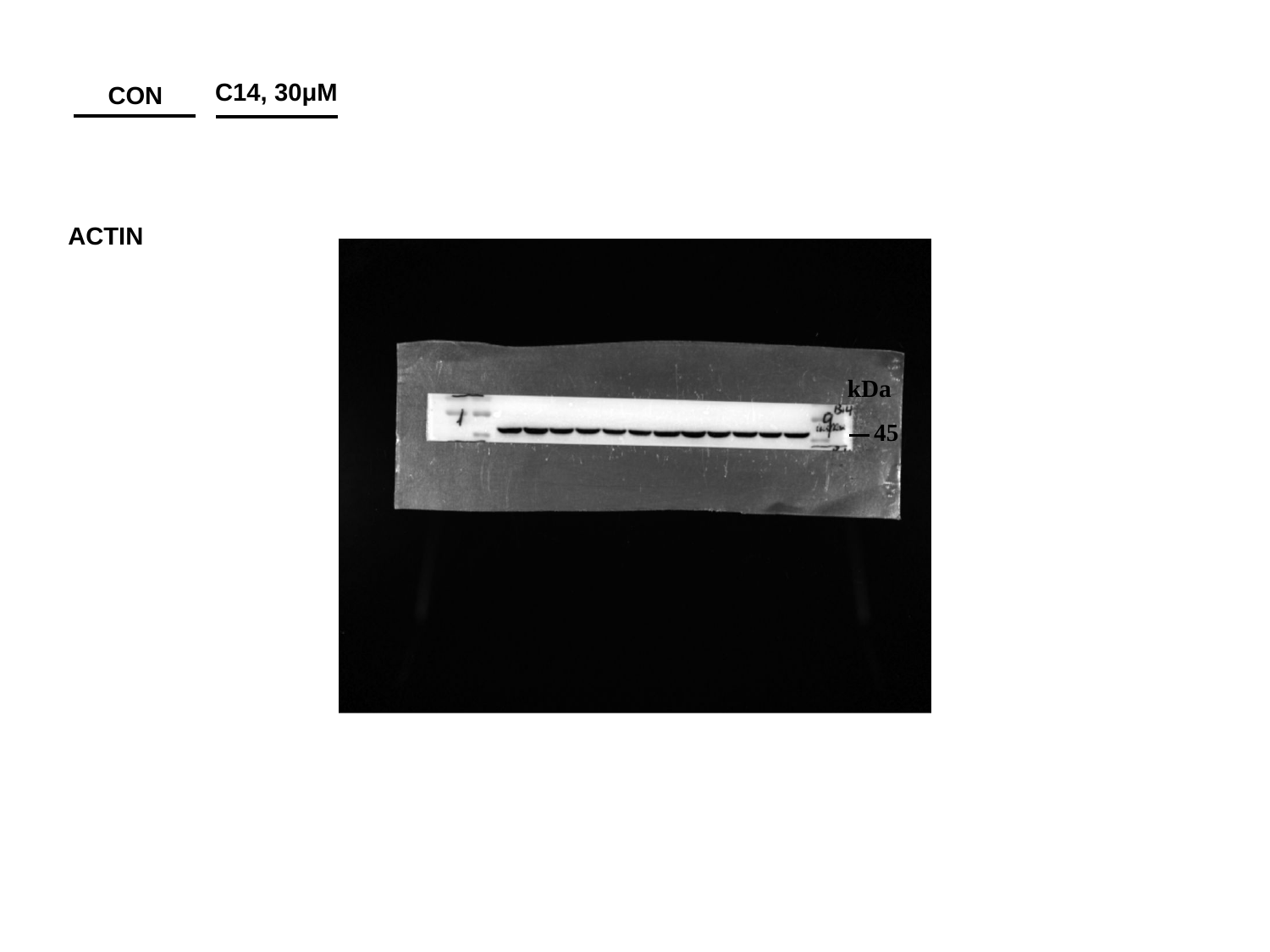

C14, 30μM
CON
ACTIN
kDa
45

## Slide 17
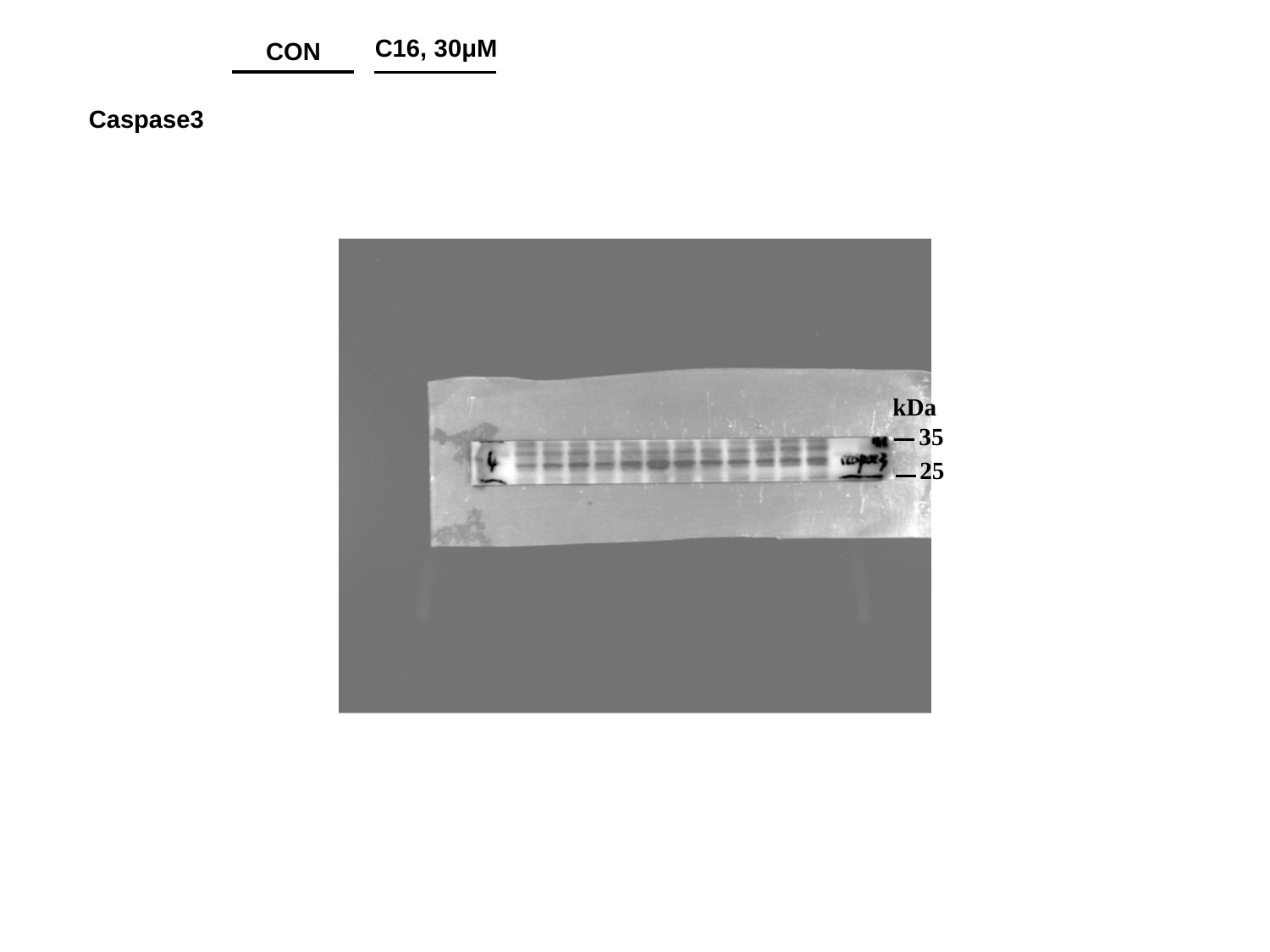

C16, 30μM
CON
Caspase3
kDa
35
25

## Slide 18
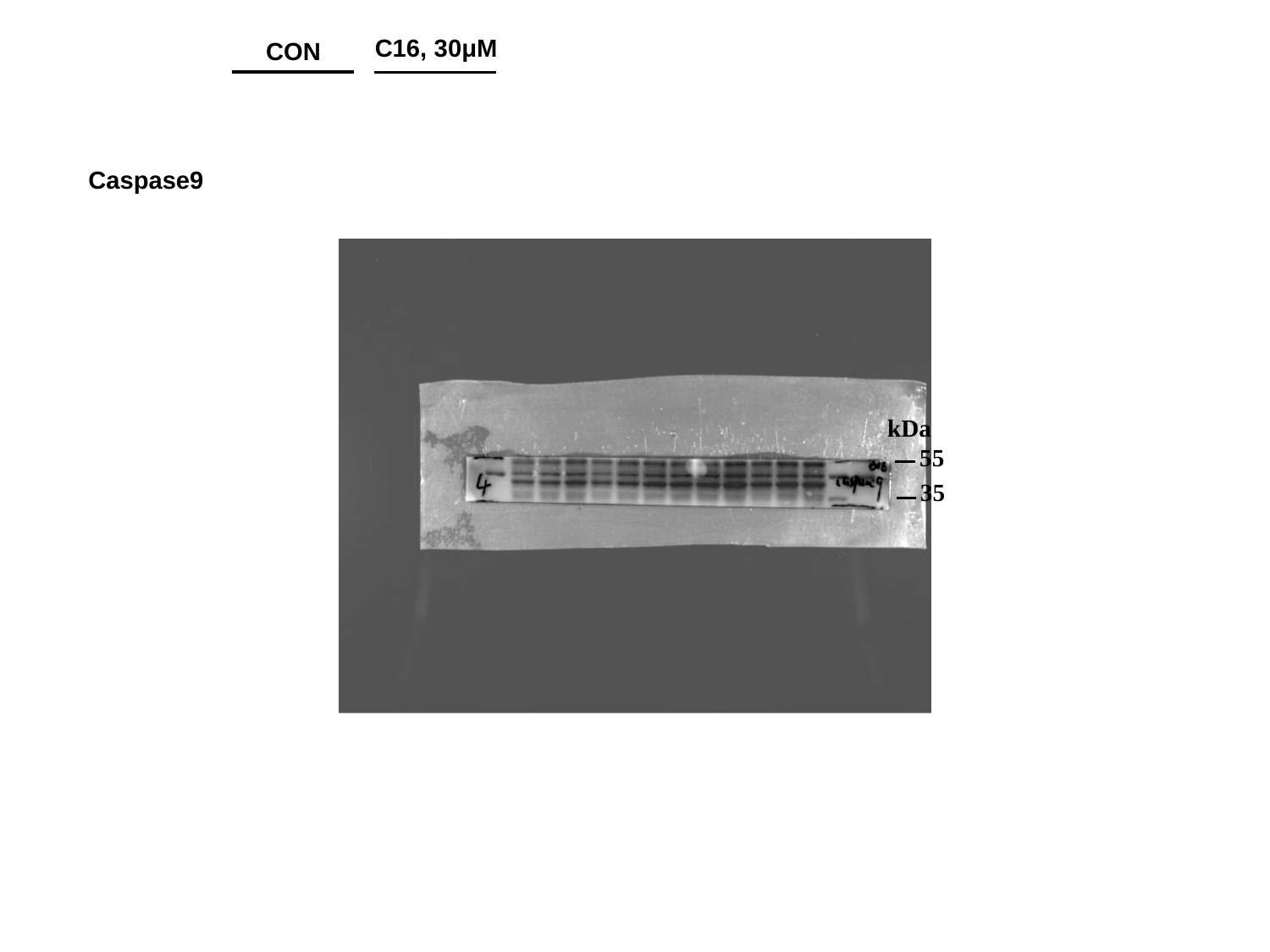

C16, 30μM
CON
Caspase9
kDa
55
35

## Slide 19
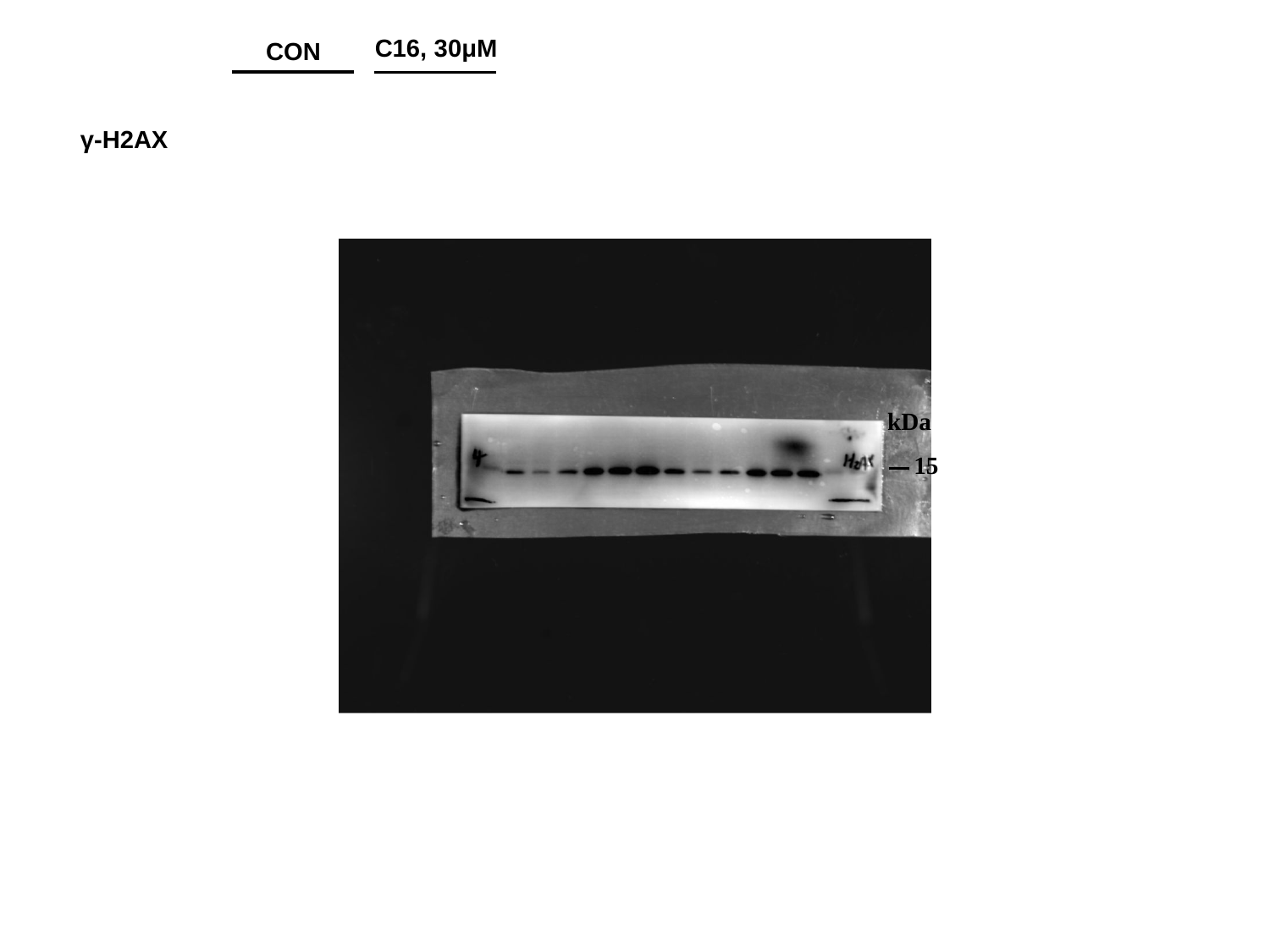

C16, 30μM
CON
γ-H2AX
kDa
15

## Slide 20
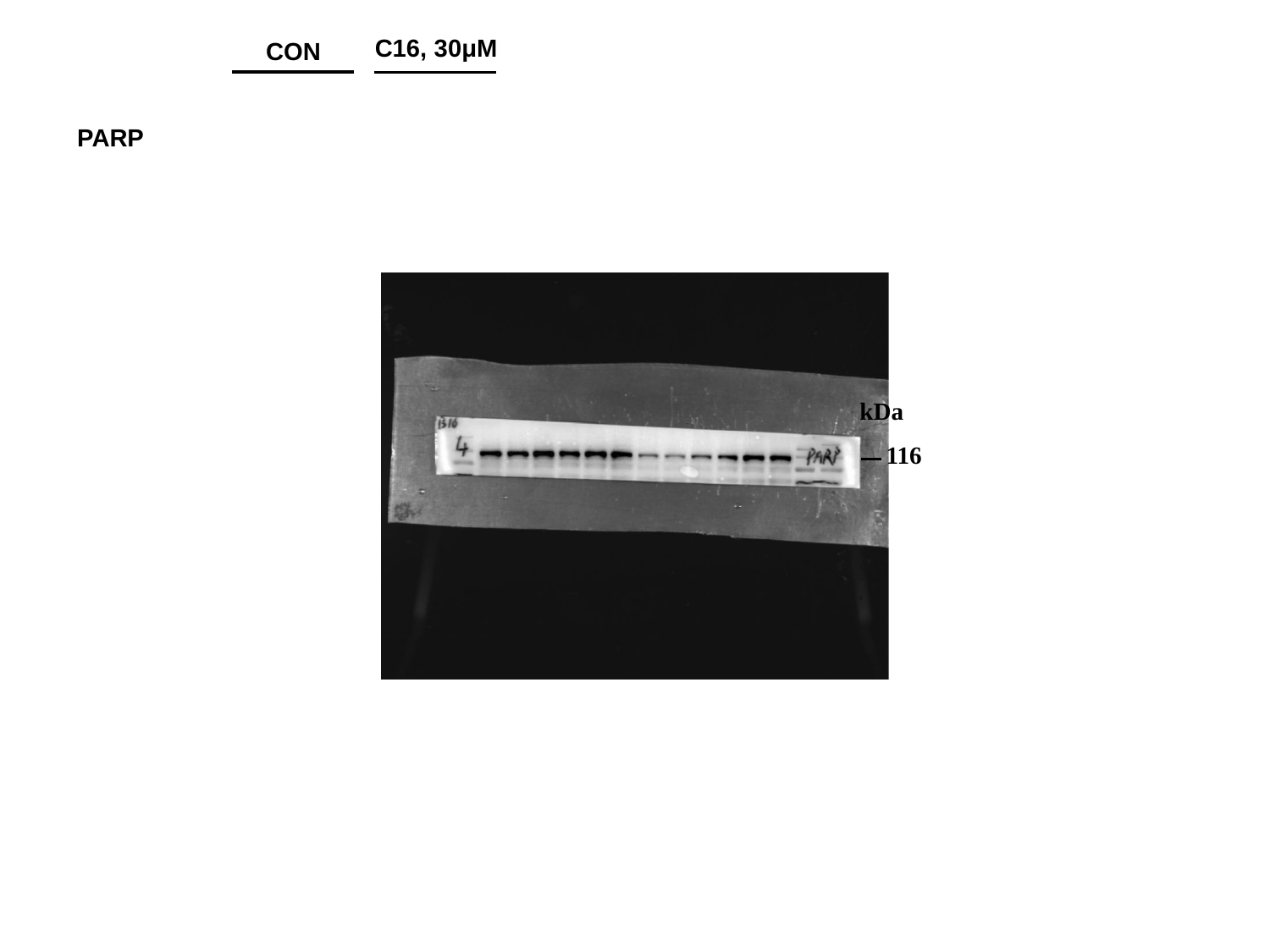

C16, 30μM
CON
PARP
kDa
116

## Slide 21
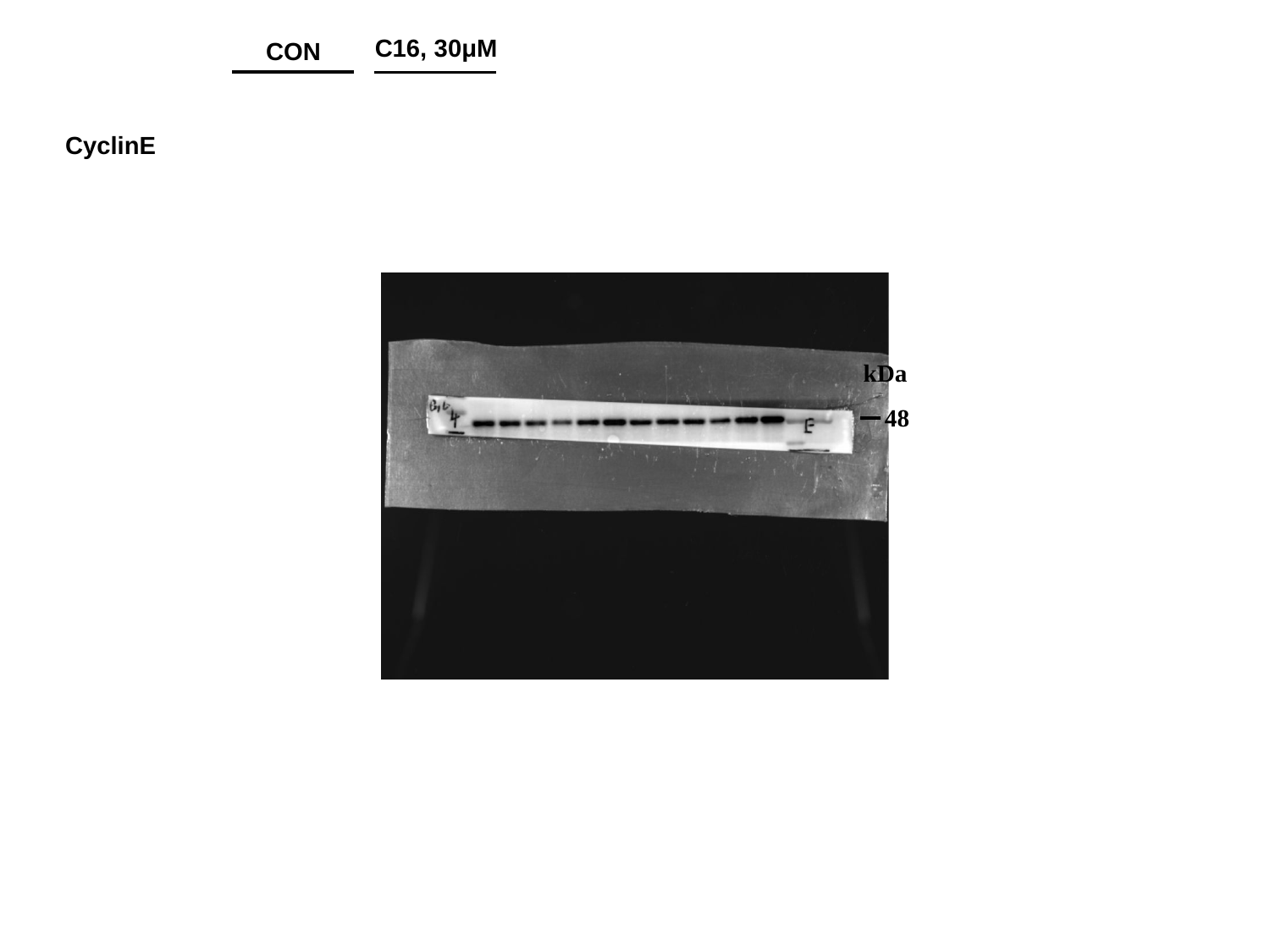

C16, 30μM
CON
CyclinE
kDa
48

## Slide 22
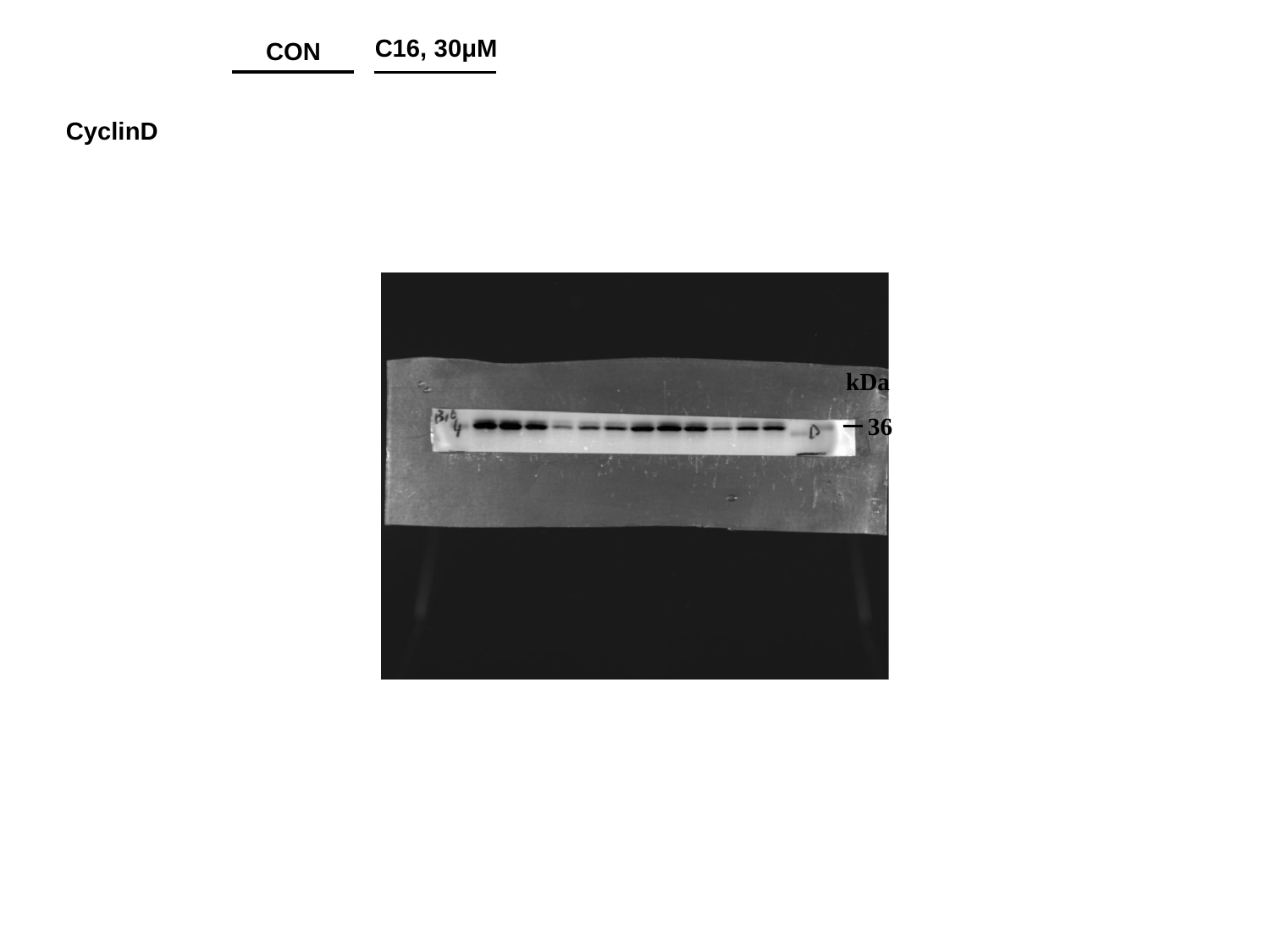

C16, 30μM
CON
CyclinD
kDa
36

## Slide 23
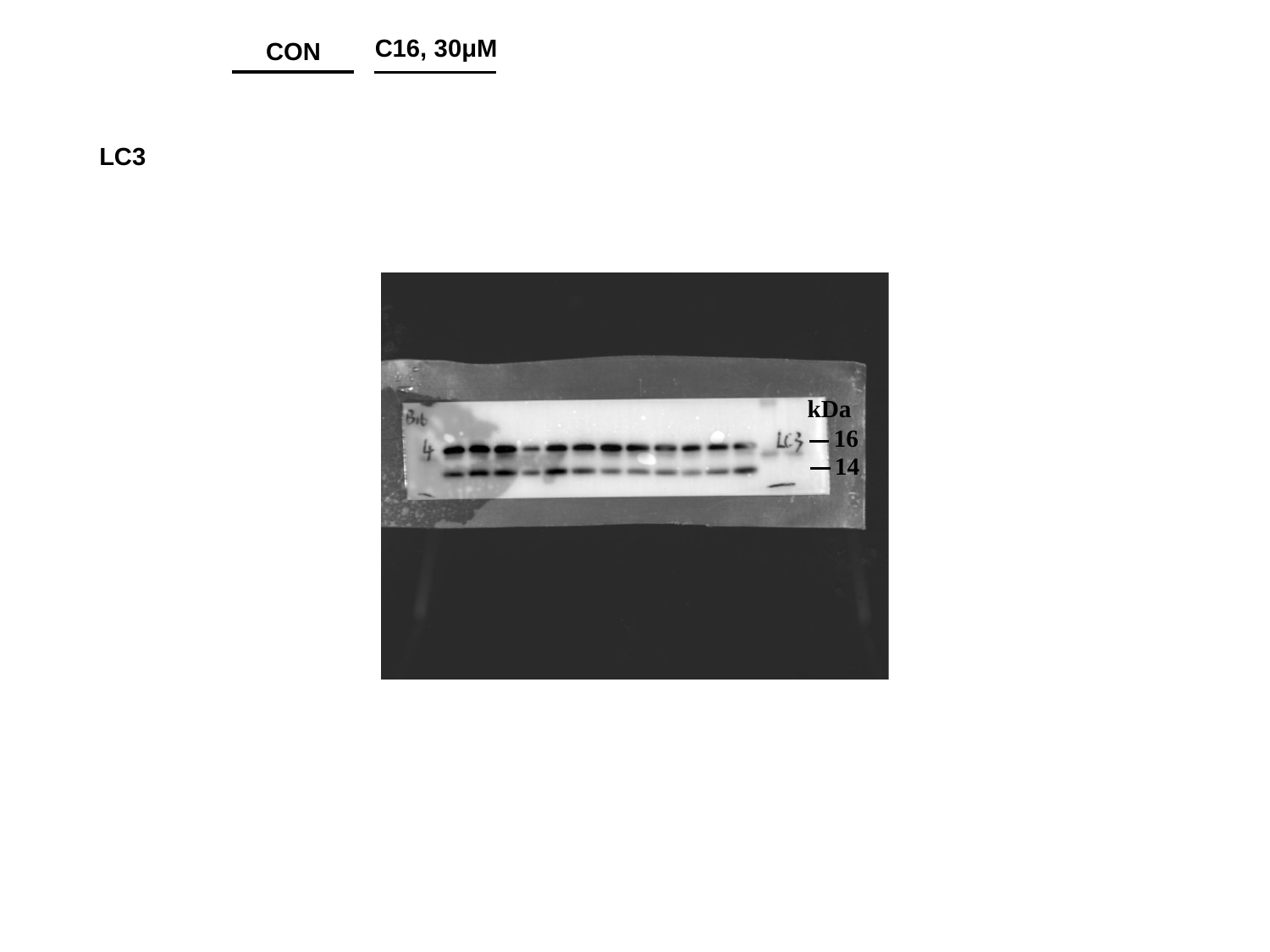

C16, 30μM
CON
LC3
kDa
16
14

## Slide 24
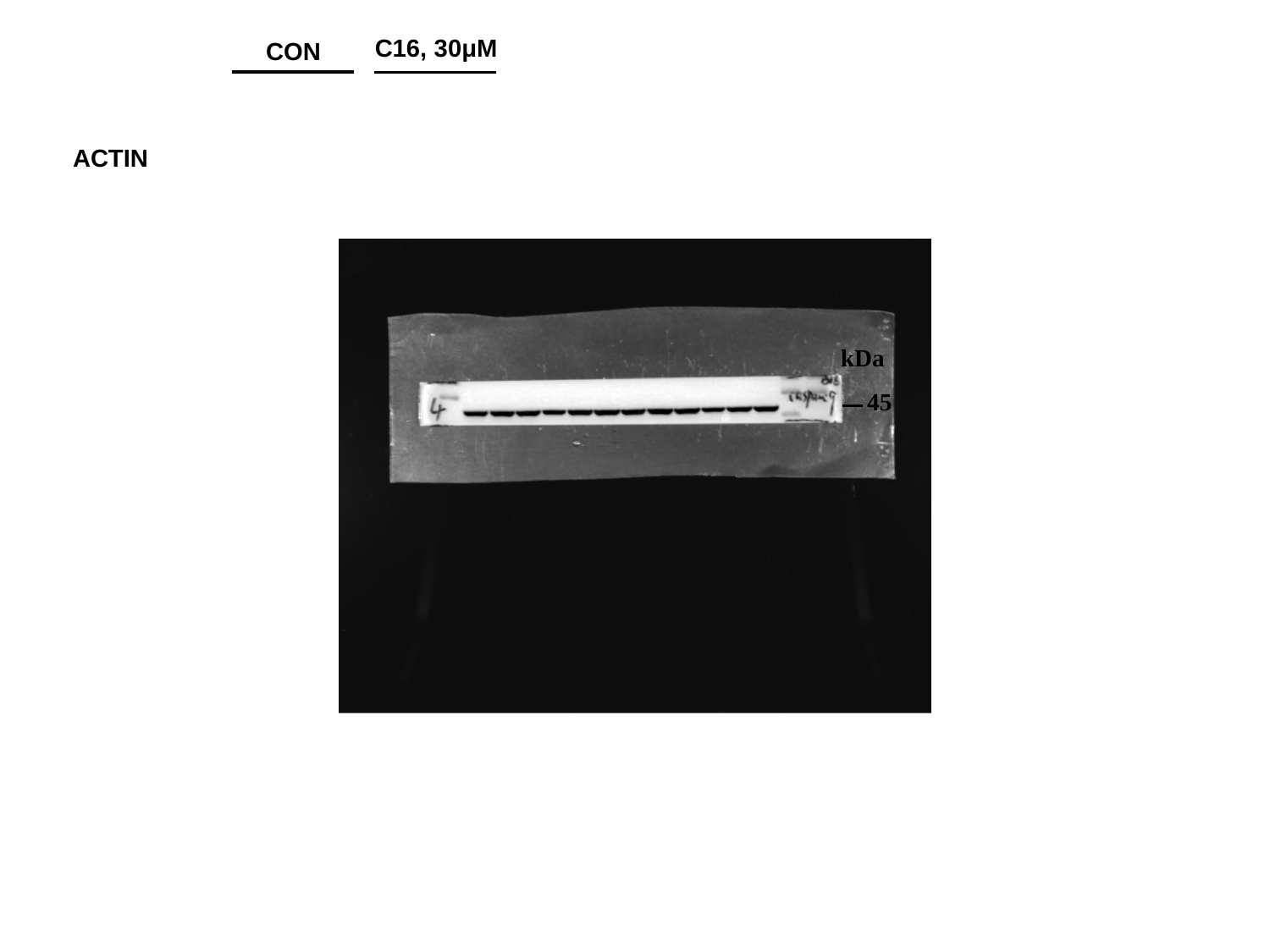

C16, 30μM
CON
ACTIN
kDa
45

## Slide 25
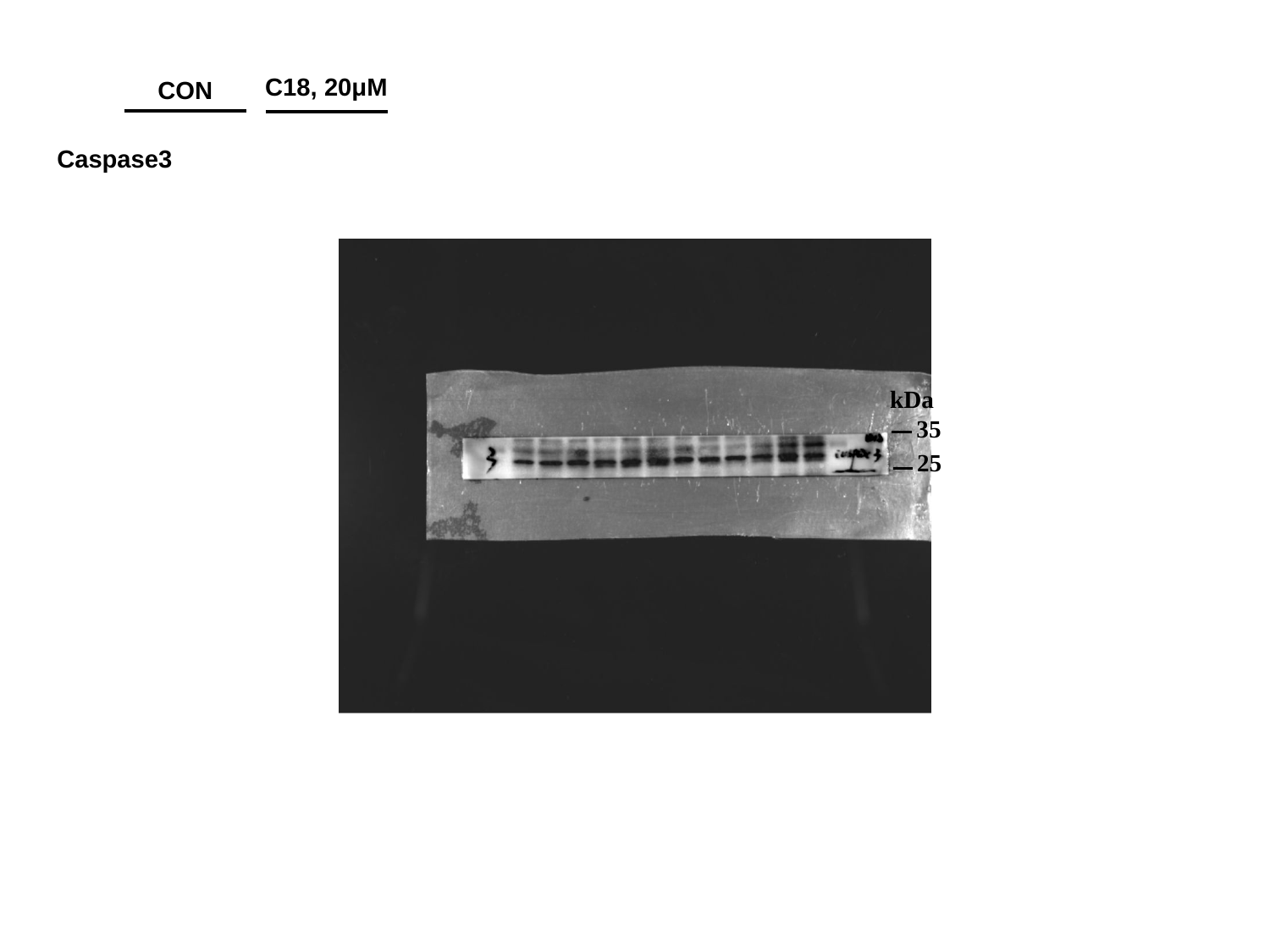

C18, 20μM
CON
Caspase3
kDa
35
25

## Slide 26
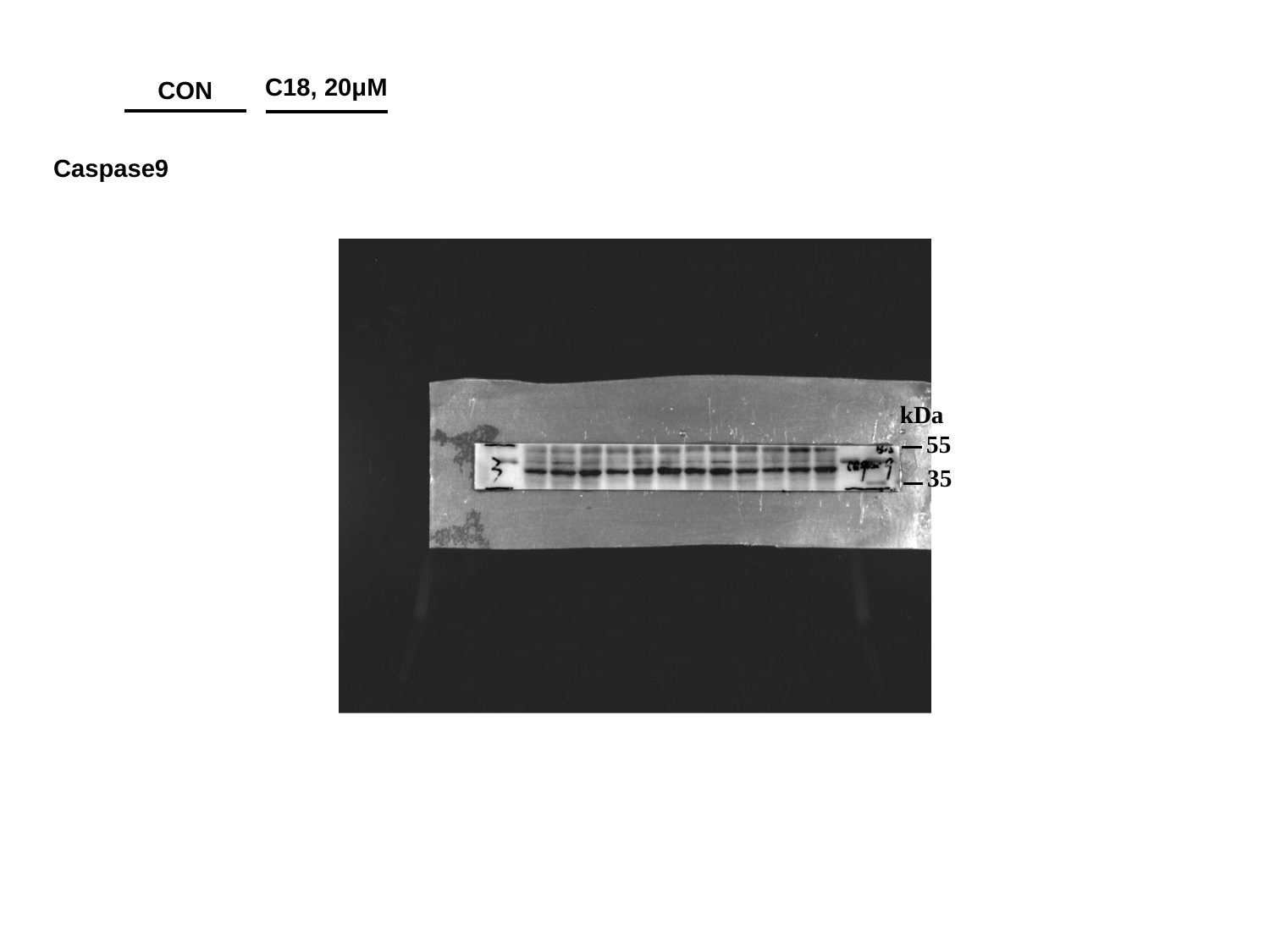

C18, 20μM
CON
Caspase9
kDa
55
35

## Slide 27
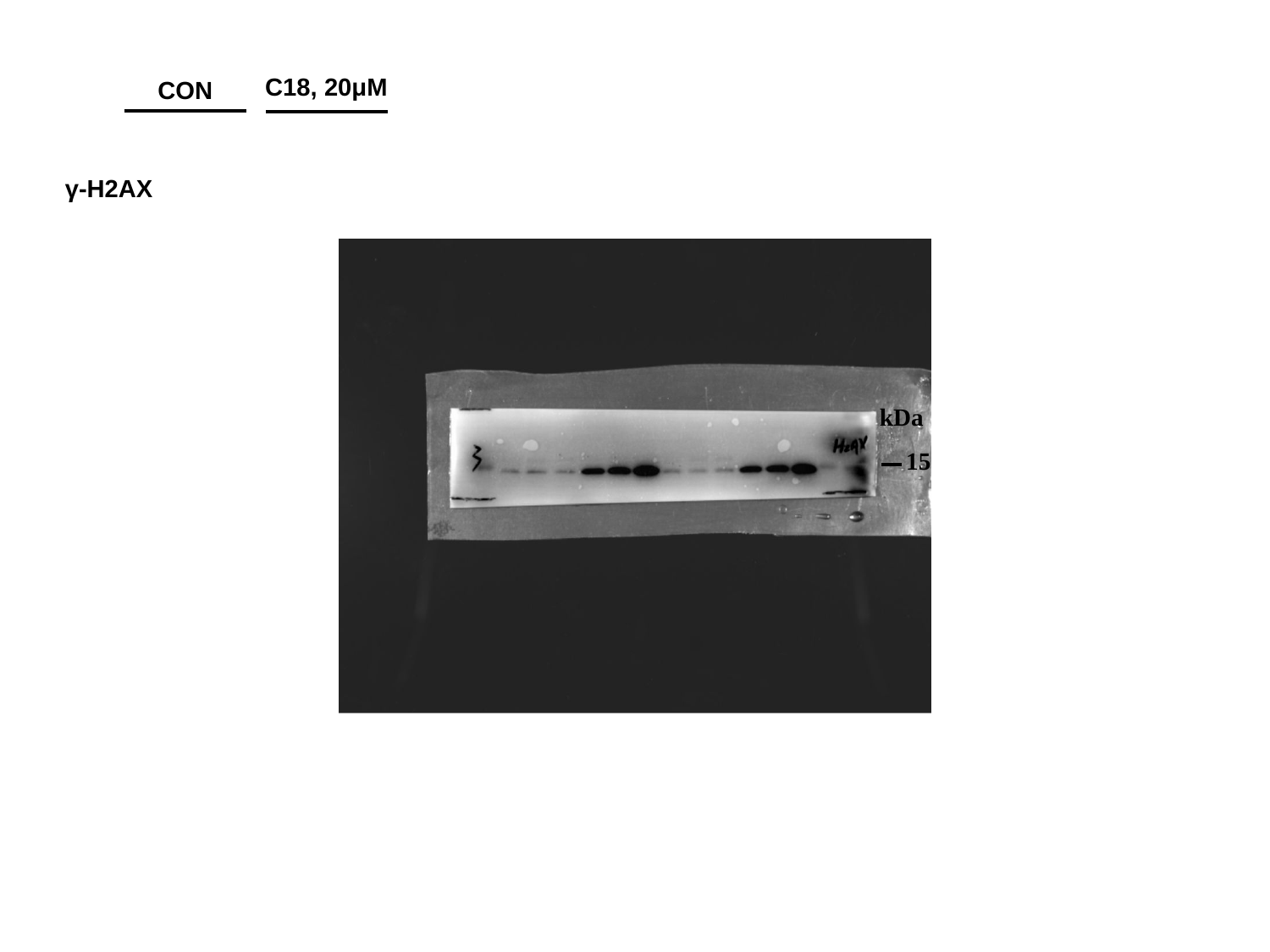

C18, 20μM
CON
γ-H2AX
kDa
15

## Slide 28
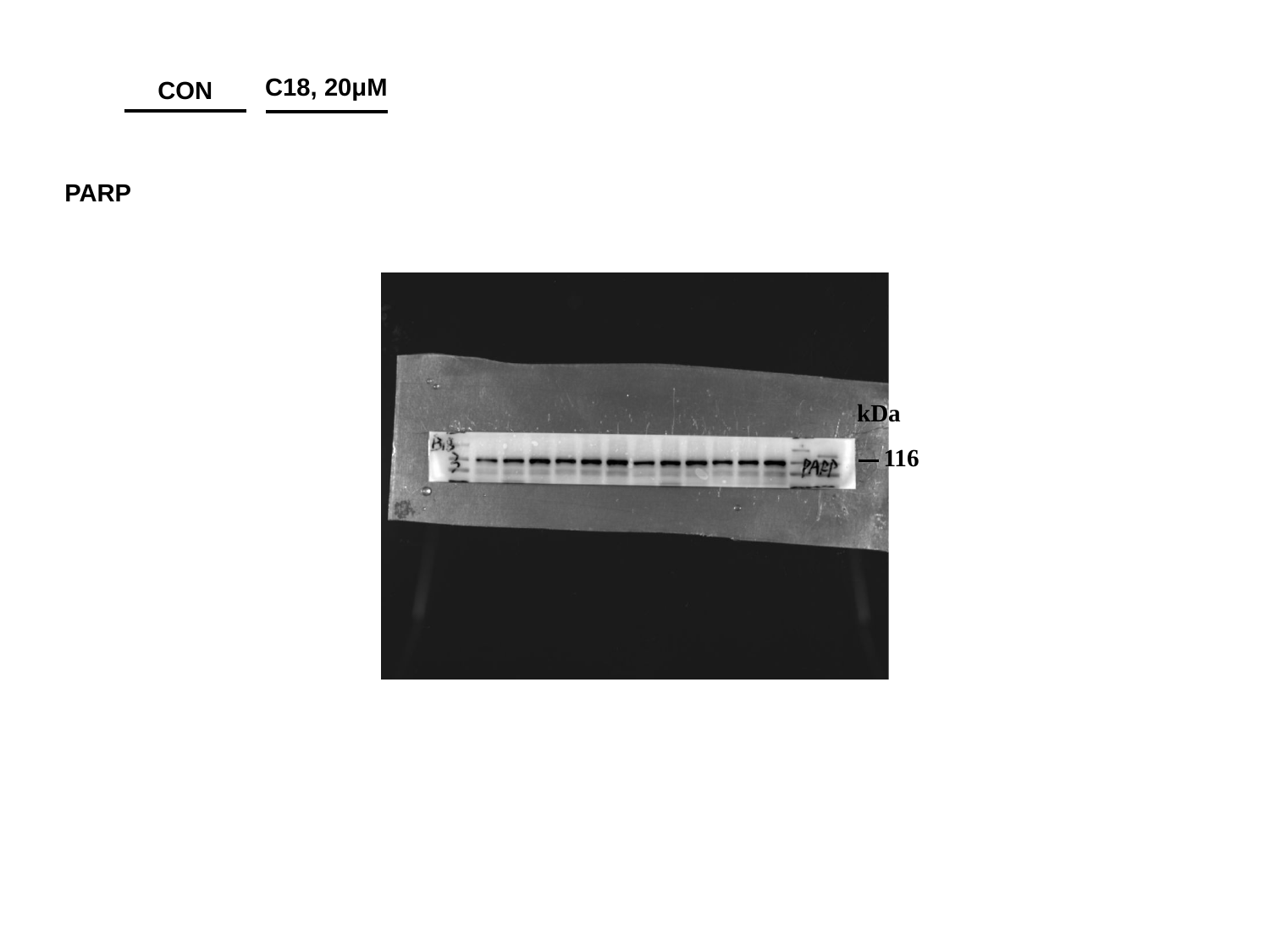

C18, 20μM
CON
PARP
kDa
116

## Slide 29
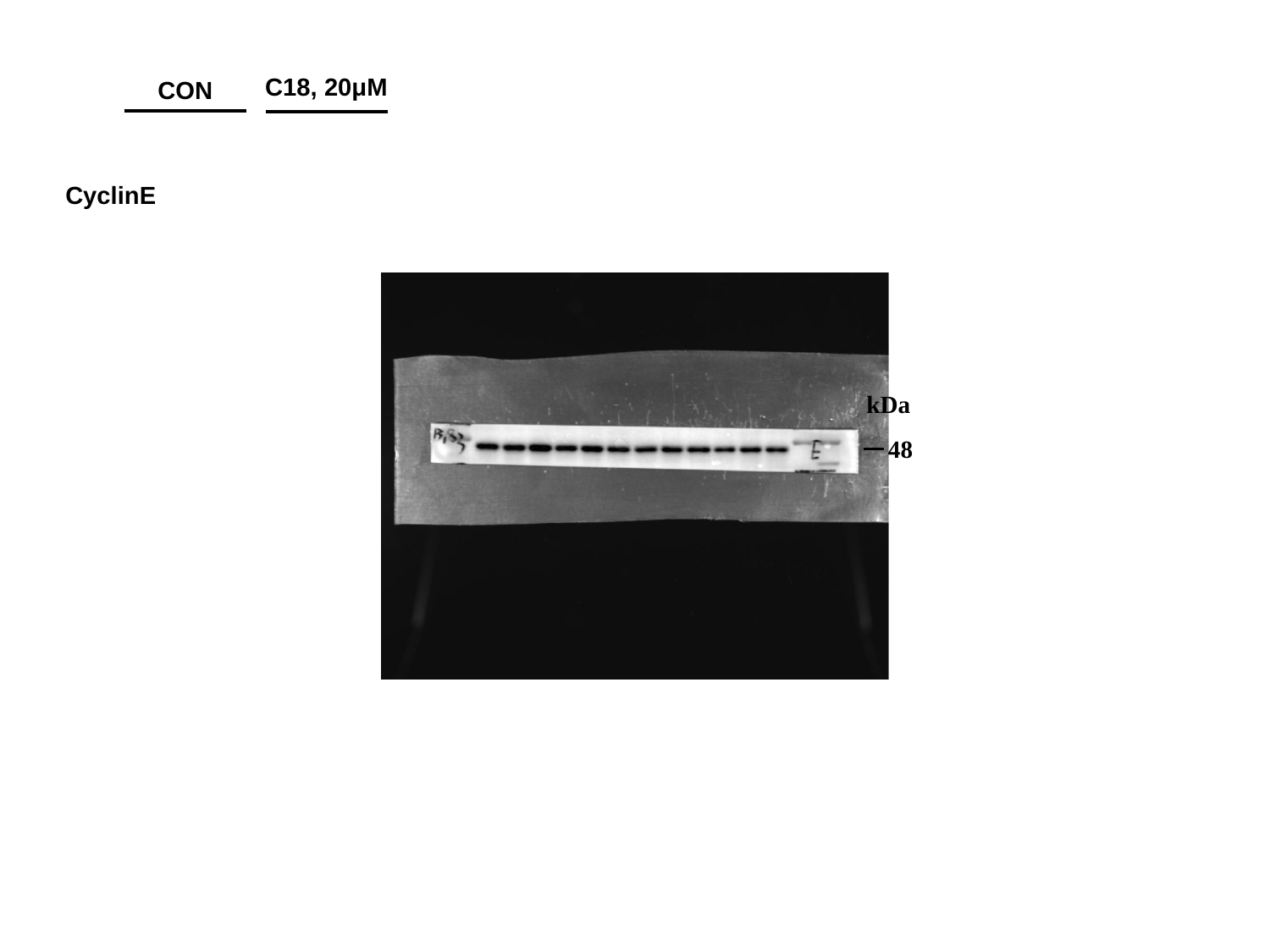

C18, 20μM
CON
CyclinE
kDa
48

## Slide 30
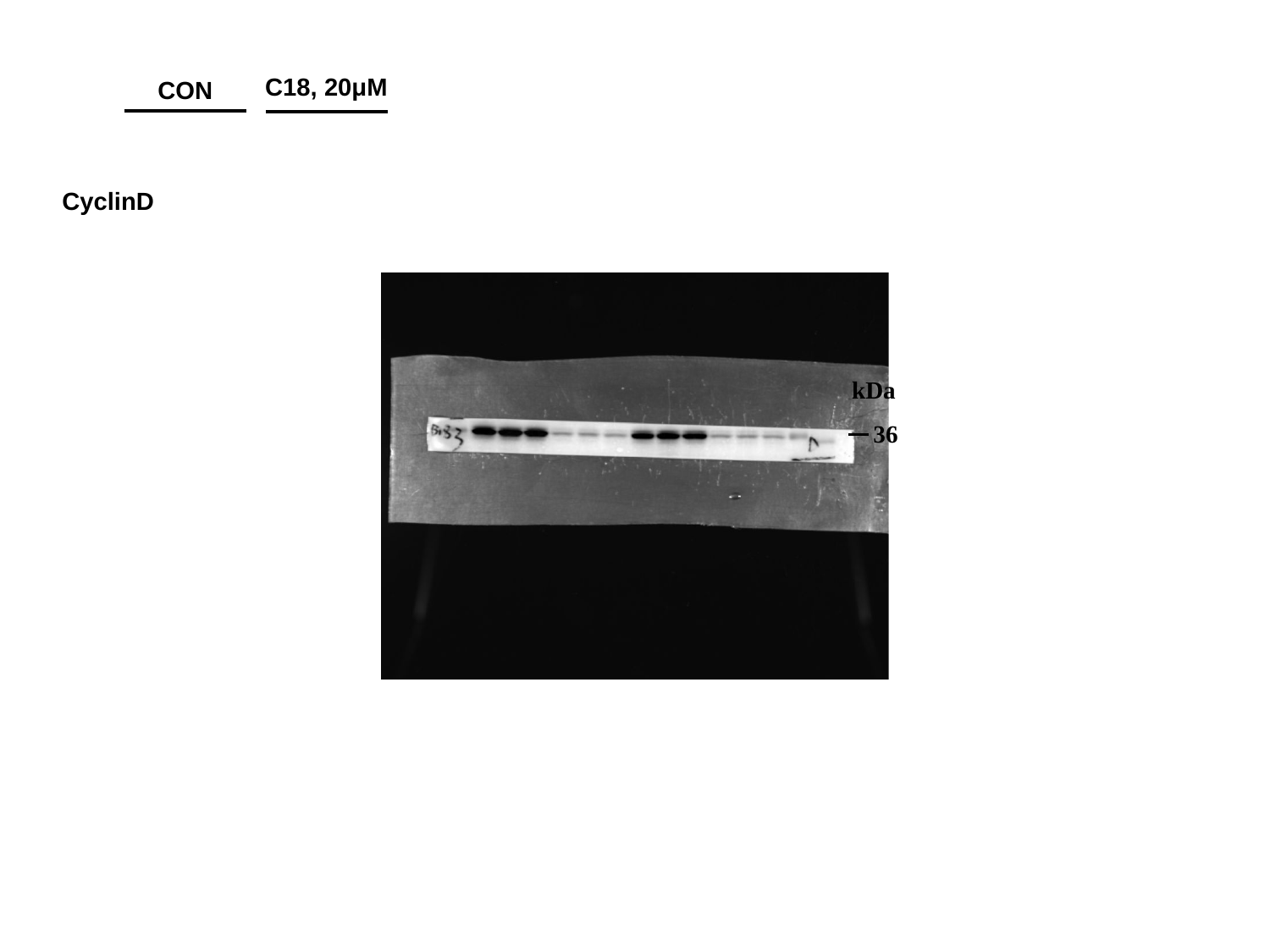

C18, 20μM
CON
CyclinD
kDa
36

## Slide 31
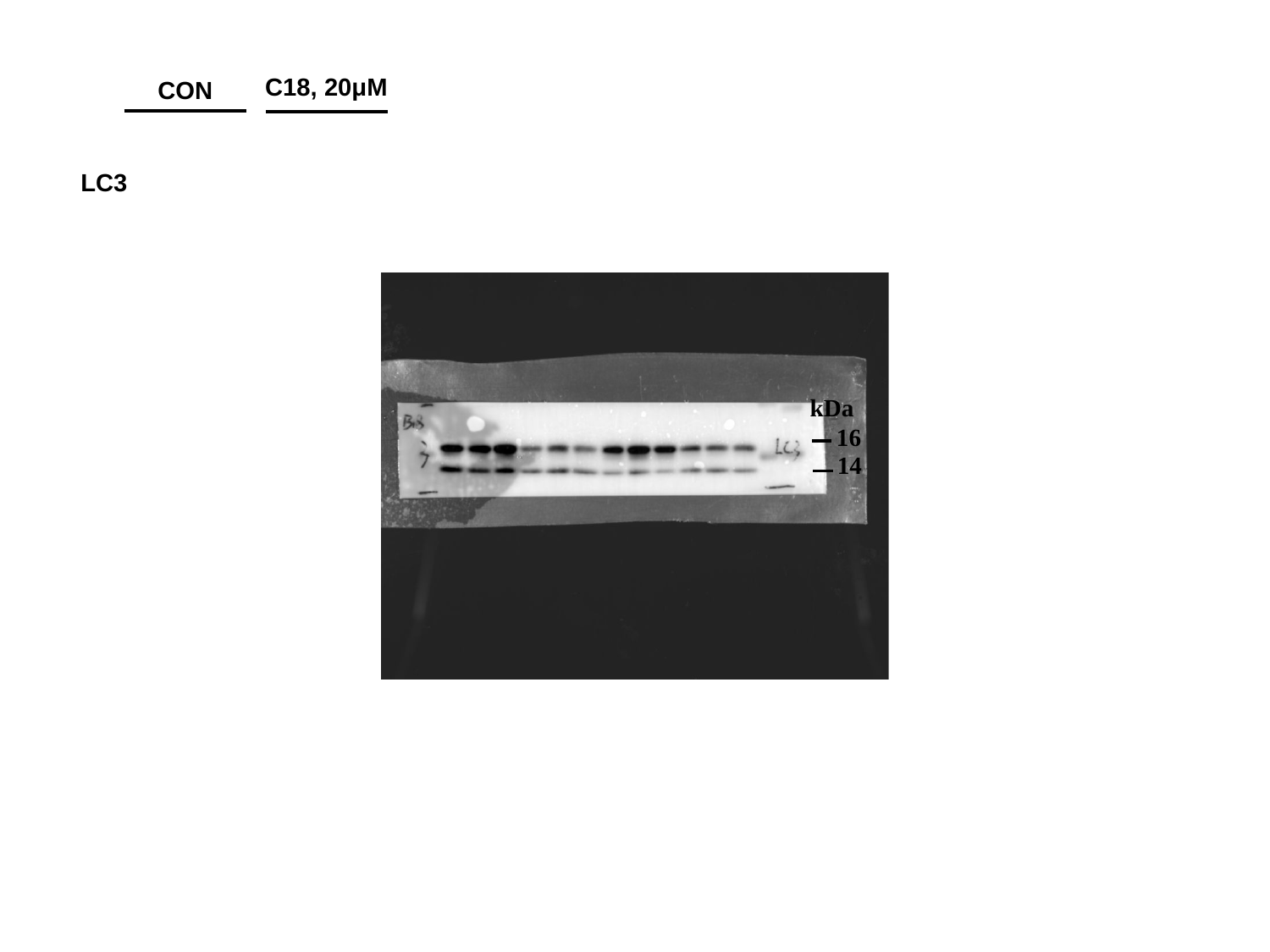

C18, 20μM
CON
LC3
kDa
16
14

## Slide 32
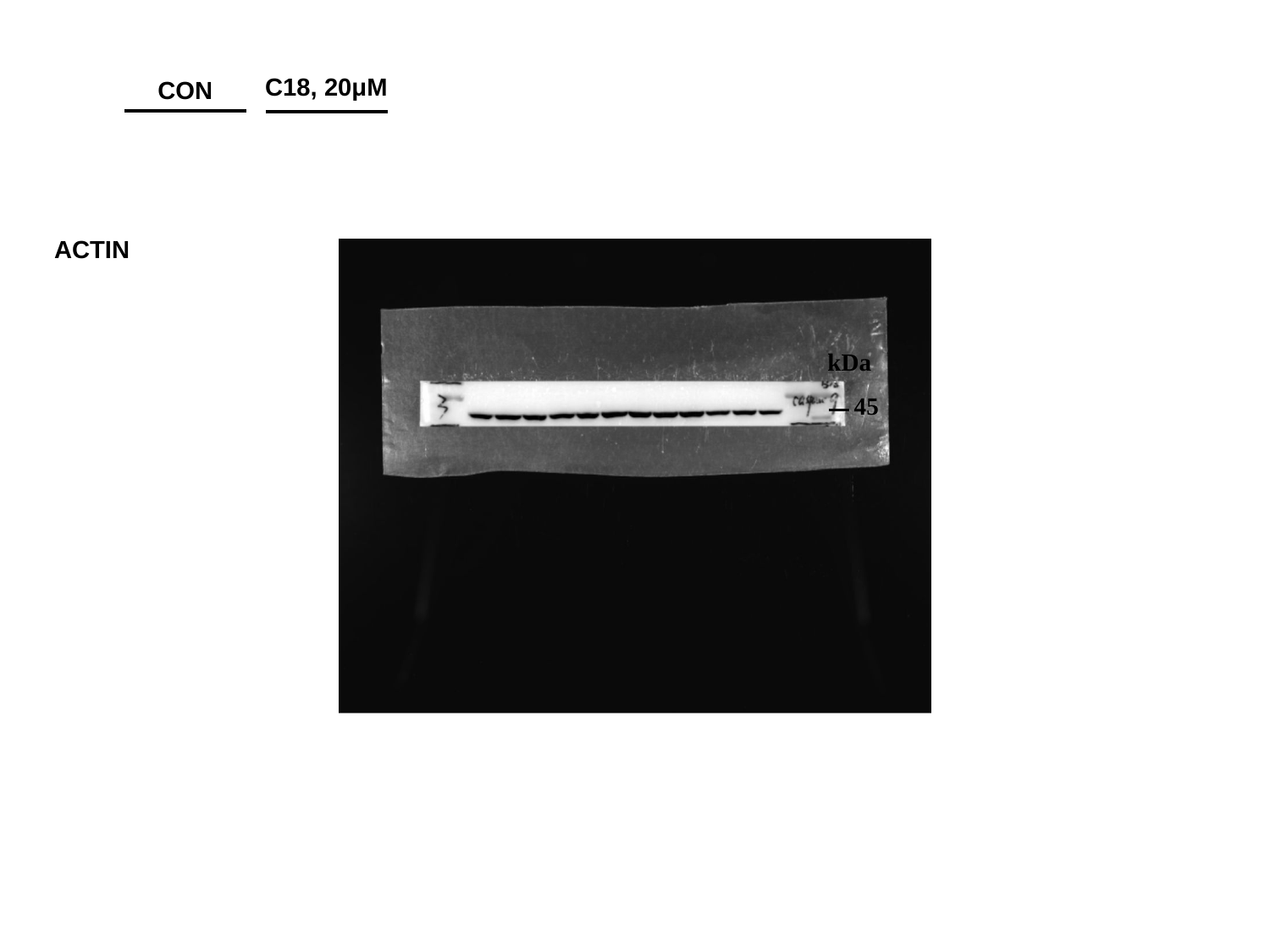

C18, 20μM
CON
ACTIN
kDa
45
